# Large-scale exome sequence analysis identifies sex- and age-specific determinants of obesity

**DOI:** 10.1016/j.xgen.2023.100362

**Published:** 2023-08-02

**Authors:** Lena R. Kaisinger, Katherine A. Kentistou, Stasa Stankovic, Eugene J. Gardner, Felix R. Day, Yajie Zhao, Alexander Mörseburg, Christopher J. Carnie, Guido Zagnoli-Vieira, Fabio Puddu, Stephen P. Jackson, Stephen O’Rahilly, I. Sadaf Farooqi, Laura Dearden, Lucas C. Pantaleão, Susan E. Ozanne, Ken K. Ong, John R.B. Perry

**Affiliations:** 1 MRC Epidemiology Unit, Wellcome-MRC Institute of Metabolic Science, University of Cambridge, Cambridge CB2 0QQ, UK; 2 MRC Metabolic Diseases Unit, Wellcome-MRC Institute of Metabolic Science, University of Cambridge, Cambridge CB2 0QQ, UK; 3 Wellcome Trust/Cancer Research UK Gurdon Institute, Department of Biochemistry, University of Cambridge, Tennis Court Road, Cambridge CB2 1QR, UK; 4 Cancer Research UK Cambridge Institute, Li Ka Shing Building, University of Cambridge, Robinson Way, Cambridge CB2 0RE, UK

**Keywords:** obesity, exome-wide association study, UK Biobank, DNA damage, rare variant, GWAS, type 2 diabetes

## Abstract

Obesity contributes substantially to the global burden of disease and has a significant heritable component. Recent large-scale exome sequencing studies identified several genes in which rare, protein-coding variants have large effects on adult body mass index (BMI). Here we extended such work by performing sex-stratified associations in the UK Biobank study (N∼420,000). We identified genes in which rare heterozygous loss-of-function increases adult BMI in women (*DIDO1, PTPRG,* and *SLC12A5*) and in men (*SLTM*), with effect sizes up to ∼8 kg/m^2^. This is complemented by analyses implicating rare variants in *OBSCN* and *MADD* for recalled childhood adiposity. The known functions of these genes, as well as findings of common variant genome-wide pathway enrichment analyses, suggest a role for neuron death, apoptosis, and DNA damage response mechanisms in the susceptibility to obesity across the life-course. These findings highlight the importance of considering sex-specific and life-course effects in the genetic regulation of obesity.

## Highlights

• Large-scale obesity exome sequence association study conducted in the UK Biobank
• Female-specific associations with rare variant burden in *DIDO1*, *PTPRG,* and *SLC12A5*
• Childhood-specific associations with rare variant burden in *MADD* and *OBSCN*
• Common variant analyses identify adult-onset obesity effects

## Graphical abstract

**Figure:**
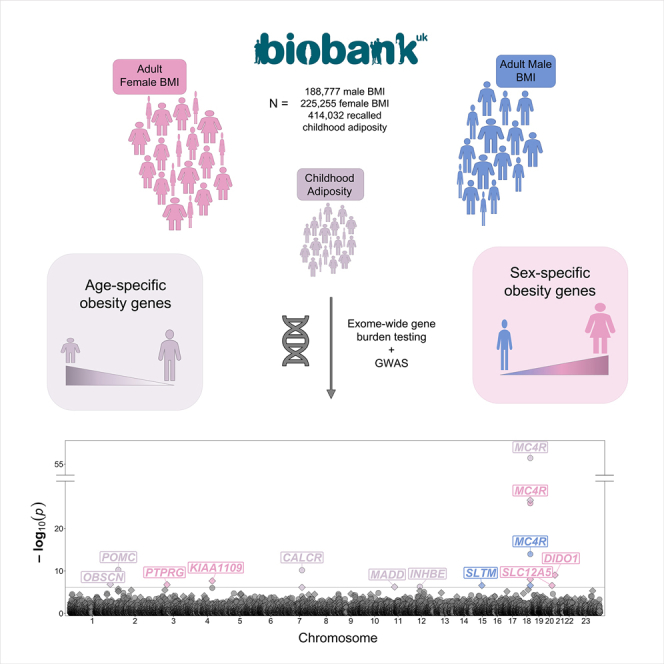

## Teaser

Kaisinger et al. queried the genomes of ∼420,000 individuals and identified genes associated with sex- and age-specific obesity risk. They highlight putative roles for DNA damage response mechanisms in obesity across the life-course, in addition to highlighting a pattern of adult-onset genetic effects.

## Introduction

Obesity is a global issue affecting over 650 million adults and 124 million children and adolescents.^1^ It is associated with increased mortality and morbidity as well as numerous comorbidities, such as cardiovascular disease and type 2 diabetes (T2D) and represents an enormous health burden. Obesity prevalence is greater in women than in men,^2^ and women tend to have more body fat that is preferentially stored as subcutaneous fat in lower body depots, whereas men are more prone to visceral fat accumulation in the abdominal region.^3^ These sex differences in adiposity affect risks for several obesity-related comorbidities, such as hypertension and T2D.^4^ Yet, sex-specific analysis in research is uncommon, with most genetic studies adjusting for sex rather than analyzing data separately for men and women.

It is estimated that around 40%–70% of inter-individual variability in body mass index (BMI) can be attributed to genetic factors.^5^^,^^6^ Very large population-based studies (N ∼700K) have identified over 900 genetic loci associated with BMI in adults.^7^ Most of those genetic variants, although common, are located in non-coding regions, and collectively explain only ∼6% of the population variance in adult BMI.^7^ The recent advent of whole-exome sequencing (WES) in large population-based studies^8^ has enabled assessment of rare coding variants in disease and related traits. The largest WES analysis for BMI to date comprised ∼620,000 adults^9^ and identified rare variants in 16 genes associated with adult BMI, including rare loss-of-function variants in *GPR75*, where 1:2,500 are heterozygous carriers and these have 1.8 kg/m^2^ lower BMI and half the odds of obesity compared with non-carriers.

The genetic determinants of childhood adiposity are less well studied due to a relative paucity of data in large-scale childhood cohorts. However, childhood obesity has an important impact on child health, and individuals who develop obesity in childhood generally tend to remain obese as adults.^10^ Studies of childhood BMI (combined sample size ∼56K) reported that many loci for adult BMI also operate in early life.^11^^,^^12^ Furthermore, some loci exhibit stronger effects on adiposity in childhood, with less or even null effect in adulthood.^11^ Across all these studies, the identified loci implicate brain-expressed genes, many acting on the leptin-melanocortin pathway, where rare heterozygous or homozygous loss-of-function of key genes are reported causes of monogenic obesity manifesting with hyperphagia in early childhood.^13^^,^^14^^,^^15^ Furthermore, large-scale genetic studies of pubertal timing, an event closely coupled with childhood adiposity status, have also identified loci and biological mechanisms influencing early growth and development.^16^^,^^17^^,^^18^^,^^19^

Here, we explored two further approaches to identify genes that regulate susceptibility to obesity: rare coding variants (1) with sex-specific effects on adult BMI, or (2) associated with childhood adiposity, using a childhood body adiposity trait that was subjectively recalled in adults (sample size ∼400K) from the UK Biobank study, and was recently reported to show high genetic correlation (r_g_ = 0.85) with objectively measured childhood BMI.^20^ Sex-specific associations with body size and metabolic disease have been described for common genetic variation,^21^^,^^22^ yet few examples exist for rarer variants, which offer greater opportunity to directly implicate causal genes. Likewise, common variant genome-wide association studies (GWASs) have been performed for recalled childhood adiposity, yet no similar study exists for rarer variants. To address this, we undertook a dual exome-wide association study (ExWAS) approach using data from up to 419,692 individuals from the UK Biobank study.

## Results

### Rare variants associated with sex-stratified adult BMI

To identify rare coding variants that exhibit sex-specific effects on adult adiposity, we performed ExWAS for adult BMI (kg/m^2^) separately in 191,864 men and 227,828 women from the UK Biobank study. Gene burden tests were performed by collapsing rare variants (minor allele frequency [MAF] < 0.1%) in individual genes according to two overlapping predicted functional categories: (1) high-confidence protein truncating variants (PTVs) and (2) PTV plus missense variants with a combined annotation dependent depletion (CADD)^23^ score ≥25 (termed “damaging variants,” DMG).The absence of significant signals (Figure S1) and inflation of test statistics (Table S1) across different allele count ranges for synonymous variant burden tests provided reassurance that our association testing models were well calibrated.

**Figure 1:**
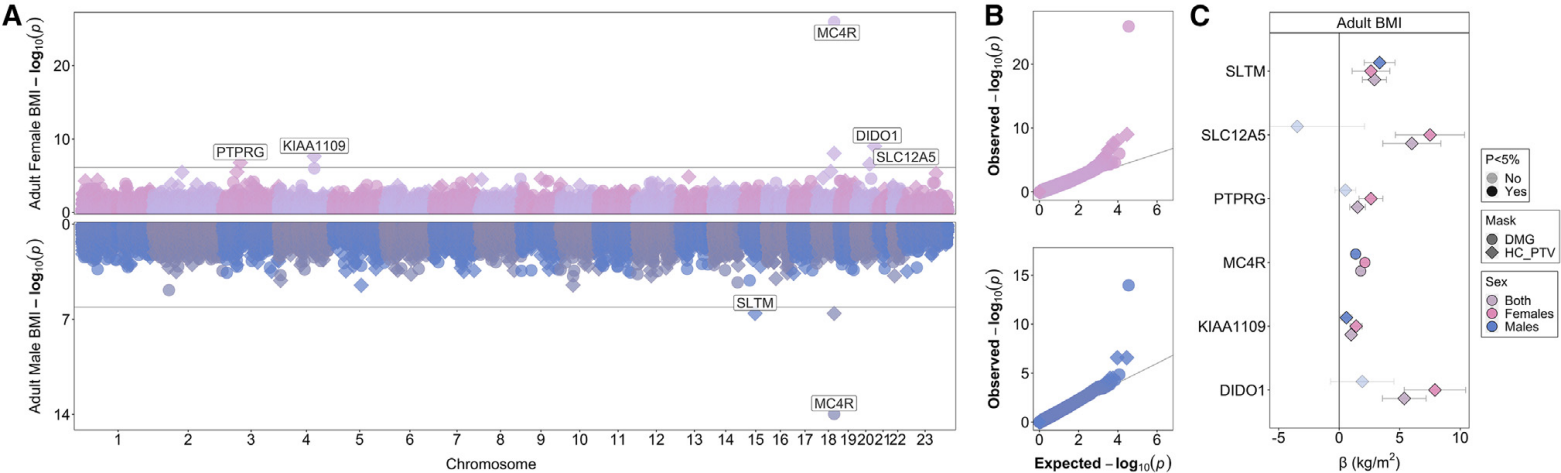
Gene burden associations of rare variants with adult BMI by sex (A) Miami plot showing significantly associated genes (Bonferroni corrected p < 7.76 × 10^−7^) separately in women (upper) and men (lower). (B) QQ plot of the same data. (C) Effect estimates and 95% confidence intervals for each identified gene. For further details, see Tables S2 and S3.

Five genes were associated with BMI in females (*DIDO1*, *KIAA1109*, *MC4R*, *PTPRG,* and *SLC12A5*) and two genes were associated with BMI in males (*MC4R* and *SLTM*) at exome-wide significance (p < 7.76 × 10^−7^; 0.05/64,396 tests (32,536 and 31,860 gene burden tests in females and males, respectively)) (Figures 1 and S2, Tables S2 and S3). Two of these genes, *MC4R* and *KIAA1109*, were reported in previous sex-combined ExWAS for BMI,^9^ and showed exome-wide significant or subthreshold associations with BMI in both sexes, as did *SLTM* (men: beta = 3.34 kg/m^2^/allele, p = 2.7 × 10^−7^, n = 38 PTV carriers; women: beta = 2.6, p = 9.5 × 10^−4^, n = 37 PTV carriers; P_sex-heterogeneity_ = 0.48).

**Figure 2:**
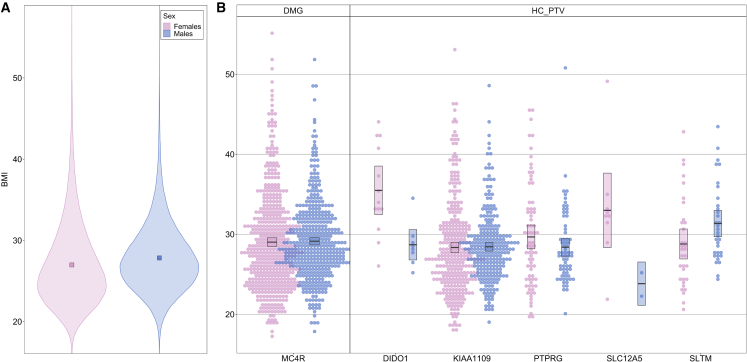
Distributions of adult BMI by sex (A) In all UK Biobank participants; (B) among carriers of rare variants (DMG, damaging; PTV, protein truncating) in genes associated with sex-stratified BMI. Mean and 95% CI for each group are indicated by horizontal bars and boxes. Summarized group data can be found in Table S22.

Rare protein-coding variants in the remaining three genes, identified for BMI in females (*DIDO1*, *PTPRG,* and *SLC12A5*), have not previously been implicated in adiposity and appear to have female-specific effects, with not even nominal association with BMI in males; in females: *DIDO1* (beta = 7.91 kg/m^2^, p = 9.5 × 10^−10^, n = 14 PTV carriers, P_sex-heterogeneity_ = 1.2 × 10^−3^), *PTPRG* (beta = 2.62 kg/m^2^, p = 1.7 × 10^−7^, n = 92 PTV carriers, P_sex-heterogeneity_ = 1.5 × 10^−3^), and *SLC12A5* (beta = 7.50 kg/m^2^, p = 2.7 × 10^−7^, n = 11 PTV carriers, P_sex-heterogeneity_ = 5.8 × 10^−4^) (Figures 1C, 2A, 2B, and S2A–S2E, Tables S2 and S3). We performed a number of sensitivity analyses to evaluate how robust these signals were to different analytical approaches (Table S4, STAR Methods). Test statistics were highly concordant for all reported genes, with the exception of *SLC12A5*. Plots along with association results for individual variants in the highlighted genes are shown in Figure S2 and Table S3.

To identify potential mechanisms underlying these observed female-specific effects, we further explored rare variant sex-stratified associations for *DIDO1*, *PTPRG,* and *SLC12A5* with free testosterone, sex-hormone binding globulin (SHBG), and waist-hip-ratio adjusted for BMI (WHRadjBMI). Female carriers of PTVs in *DIDO1* have a stronger association with circulating free testosterone concentrations (beta = 0.51, p = 9.8 × 10^−3^) than their male counterparts (beta = 0.001, p = 0.99, P_sex-heterogeneity_ = 2.7 × 10^−2^) as well as with WHRadjBMI (females: beta = −0.04, p = 1.3 × 10^−2^; males: beta = 0.02, p = 0.29; P_sex-heterogeneity_ = 3.0 × 10^−4^). Conversely, male carriers of PTVs in *PTPRG* have a stronger association with WHRadjBMI (beta = −0.02, p = 2.4 × 10^−3^; P_sex-heterogeneity_ = 8.8 × 10^−3^) than their female counterparts (beta = −0.001, p = 0.92) (Table S5). Women carrying PTVs in *SLC12A5* had higher odds of T2D than non-carriers (odds ratio [OR] 17.1 [4.3–67.5], P_glm_ = 5.2 × 10^−5^) with four of nine having T2D (UK Biobank T2D prevalence in females = 5.6% [12,675/227,363], P_Exact_ = 7.9 × 10^−4^, Table S5). In contrast, we identified only two males (both non-obese and non-diabetic) carrying a PTV in *SLC12A5*. Unlike the *SLC12A5* BMI association, test statistics for this T2D association were consistent across sensitivity analyses (Table S5). None of the female-specific BMI-associated genes showed an association with menopausal status (Table S6).

**Figure 3:**
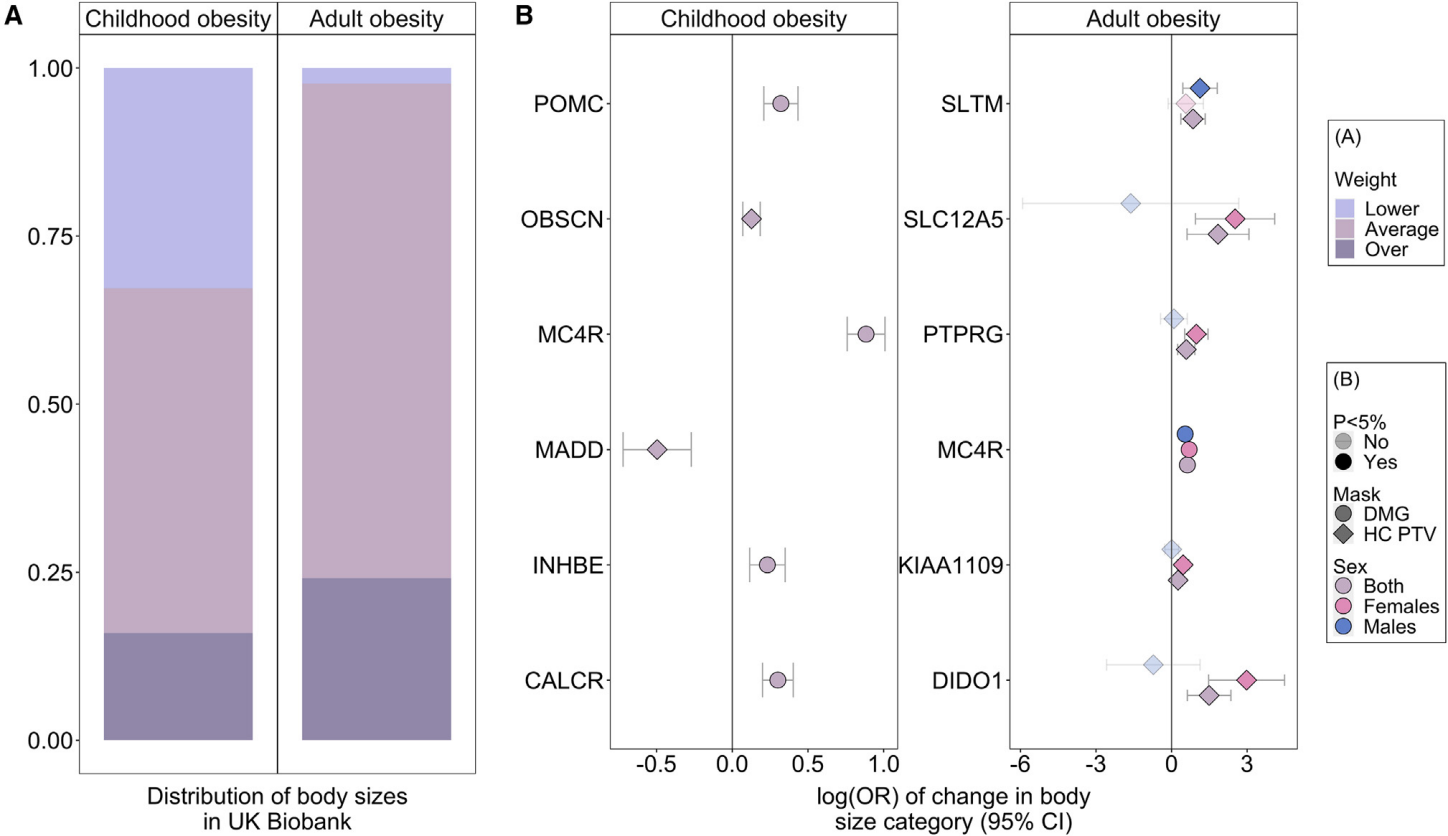
Adult and childhood obesity risk in carriers of rare damaging variants in the exome-identified genes (A) Comparative size at age 10; “Thinner,” “Average,” or “Plumper” was treated as an ordered categorical outcome to indicate childhood obesity. Adult BMI was similarly split into three categories: <20, >20 but <30, and >30. (B) These two categorical outcomes were tested in cumulative link models against carrier status for qualifying rare exome variants. Displayed log(OR) with 95% CIs and underlying data can be found in Table S7.

The prevalence of obesity (BMI >30 kg/m^2^) among carriers of DMG variants in *MC4R* was 39% (228 of 591) in females and 38% (195 of 518) in males, with ORs of 2.01 [1.68–2.41] and 1.71 [1.41–2.08], respectively (Figures 3A and 3C, Table S7). This is substantially lower than previously reported penetrance of *MC4R* variants that cause partial or complete loss-of-function *in vitro*.^13^ By contrast, the prevalence of obesity among female carriers of PTV variants in *DIDO1* and *SLC12A5* was more than 80%, albeit there were relatively fewer carriers (12 of 14 and 9 of 11 carriers were obese, respectively) (P_Heterogeneity_ = 9.9 × 10^−6^ and P_Heterogeneity_ = 2.6 × 10^−4^, respectively) (Table S7).

In the absence of sufficiently large ExWAS replication cohorts, we sought supporting evidence for our identified genes by examining independent common (MAF >0.1%) genetic variant (GWAS) associations with BMI. Four of our six identified ExWAS genes (*DIDO1*, *MC4R*, *SLC12A5,* and *SLTM*) mapped to within 500 kb of a common GWAS signal for sex-combined BMI (Figure S3, Table S8) and *DIDO1* and *MC4R* were also supported by gene-level associations between common non-synonymous variants and BMI (p = 3.8 × 10^−5^ and p = 5.0 × 10^−10^, respectively). Furthermore, the lead GWAS SNP at the *DIDO1* locus (rs6011457, p = 2.4 × 10^−10^) is intronic in *DIDO1*, is correlated with known enhancers for *DIDO1,*^24^ and exhibits a stronger association with BMI in women (p = 3.2 × 10^−8^) than BMI in men (p = 4.3 × 10^−3^, P_het_ = 0.029). At the *SLTM* locus, we observed colocalization between common variant associations for BMI and *SLTM* expression (H4 posterior probability = 0.975, see STAR Methods), where variants that decrease *SLTM* expression increase BMI, which is directionally concordant with the rare variant association (Table S8).

### Rare variants associated with childhood adiposity

We next undertook an ExWAS for childhood adiposity in 414,032 European genetic-ancestry adult UK Biobank study participants using the variable “comparative body size at age 10” (SAC10), which comprises responses to the question: “*When you were 10 years old, compared to average would you describe yourself as thinner, plumper, or about average?*” Although this is a recalled and non-quantitative indicator of childhood adiposity, it is reported to show strong genetic correlation with objectively measured childhood BMI (r_g_ = 0.85).^20^ We confirmed this in data from a larger childhood sample (r_g_ = 0.94, N = 35,668),^25^ and thus consider it to represent a robust trait for genetic analysis of childhood adiposity.

**Figure 4:**
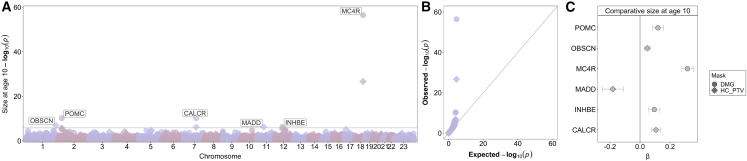
Gene burden associations of rare variants with comparative size at age 10 (A) Manhattan plot showing significantly associated genes (Bonferroni corrected p < 1.47 × 10^−6^). (B) QQ plot of the same data. (C) Effect estimates and 95% confidence intervals for each identified gene. For further details, see Tables S2 and S3.

**Figure 5:**
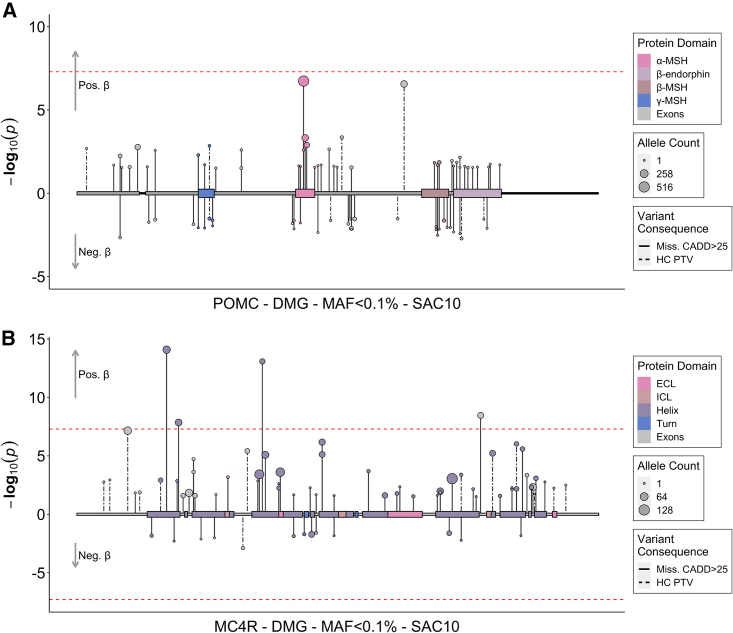
Exome associations between the functional domains of *POMC, MC4R* and SAC10 in the UK Biobank Included variants in the *POMC* (A) and *MC4R* (B) genes from our discovery analyses had a minor allele frequency (MAF) smaller than 0.1% and were annotated to be either high-confidence protein truncating variants or missense variants with a high CADD score (≥25). Each variant is presented as an individual line extending to its association p value (−log10), in the direction indicating the direction of effect on SAC10 in carriers of the alternate allele, while the point size indicates the comparative number of carriers of each variant (i.e., allele count), as indicated in the figure legend. Domain-level association statistics can be found in Table S9.

In a sex-combined ExWAS, six genes were associated with SAC10 (*CALCR*, *INHBE*, *MADD*, *MC4R*, *OBSCN,* and *POMC*) at exome-wide significance (p < 1.47 × 10^−6^, 0.05/34,127 tests) (Figures 4, 5, and S4, Tables S2 and S3). Two of these genes have been reported as disrupted in individuals with severe early-onset obesity^13^^,^^14^: *MC4R* (beta = 0.32, p = 3.7 × 10^−57^, N = 1,102 DMG carriers; OR 2.42 [2.14–2.74]) and *POMC* (beta = 0.12, p = 5.6 × 10^−11^, n = 1,303 DMG carriers (OR 1.38 [1.23–1.54]) (Figures 3A, 3B, 4C, and 5). Overall gene-level associations appeared to be driven by variants within specific sub-domains, for *POMC* by variants that encode the α-MSH peptide, and for *MC4R* by variants within its intramembrane domains and particularly helix 1 and 4 (Figure 5, Table S9). We also observed concordant associations with previously reported gain- and loss-of-function variants in *MC4R*^26^ as well as with gain-of-function variants in *POMC*^27^ (Tables S9 and S10).

Two further genes have previously been implicated in adiposity phenotypes: *CALCR* (beta = 0.11, p = 6.7 × 10^−11^, n = 1,636 DMG carriers; OR 1.35 [1.22–1.50]) was reported in an ExWAS for adult BMI^9^ and *INHBE* (beta = 0.10, p = 5.0 × 10^−7^, n = 1,199 DMG carriers; OR 1.26 [1.12–1.42]) was reported in an ExWAS for WHRadjBMI^28^(Figures 3A, 3B, and 4C).

Rare variants in the two remaining genes associated with SAC10 have not previously been implicated in childhood adiposity or body size: *MADD* (beta = −0.18, p = 5.9 × 10^−7^, n = 327 PTV carriers) and *OBSCN* (beta = 0.05, p = 1.4 × 10^−7^, n = 4954 PTV carriers) (Figure 4C). Of the 4,954 individuals with a PTV in *OBSCN*, we identified one homozygous and 25 putative compound heterozygous individuals, who together had higher odds of being plumper as a child compared with non-carriers (OR = 2.45 [1.20–4.97], p = 0.013), which is substantially higher than the odds of heterozygous carriers compared with non-carriers (OR = 1.13 [1.07–1.20], p = 3.0 × 10^−5^) (Tables S11 and S12). *OBSCN* encodes one of three giant sarcomeric signaling proteins and is predominantly expressed in skeletal muscle^29^ where it plays a role in the organization of myofibrils during assembly.^30^ Biallelic loss-of-function variants have been identified in young and predominantly physically active individuals with rhabdomyolysis.^31^ We additionally observed an association for heterozygous *OBSCN* mutations with greater measured hand-grip strength (0.58 kg ± 0.01, p = 3.2 × 10^−9^, n = 5,006 PTV carriers, Table S5), which might suggest a predominant effect on early muscle fiber development rather than adiposity.

We sought supporting evidence for our identified SAC10 ExWAS genes by assessing common genetic variant associations with SAC10 in the UK Biobank. Five of the six genes identified by ExWAS (*CALCR*, *INHBE*, *MADD*, *MC4R,* and *POMC*) map to within 500 kb of a common GWAS signal for SAC10 (Figure S5, Table S8). Furthermore, common non-synonymous variants in four of these genes (*CALCR*, *MADD*, *MC4R,* and *POMC*) showed gene-level associations with SAC10 (Table S8).

### Comparison of rare variant associations between childhood adiposity and adult BMI

**Figure 6:**
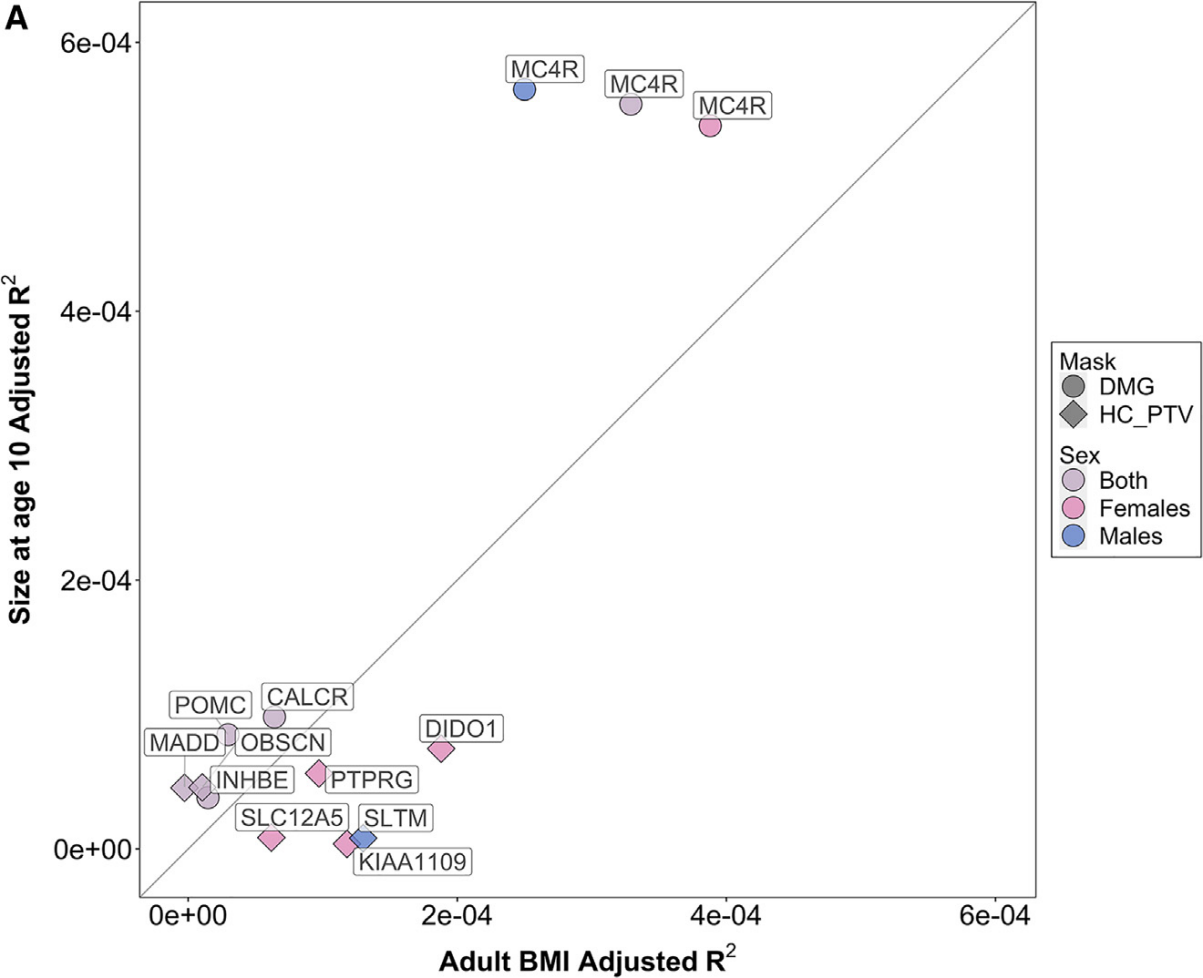
Comparison of rare variant gene-level effects on adult BMI and comparative size at age 10 For each identified exome gene, the adjusted R^2^ for carrier status of qualifying rare exome variants against residual variance in the outcome phenotype after adjusting for covariates. For each gene, the “discovery” trait-sex combinations are shown. Underlying data can be found in Table S13.

Previous work reported substantial overlap in common variant associations between childhood and adult BMI,^12^^,^^25^^,^^32^ consistent with the strong tracking of childhood overweight into adulthood^10^ with all monogenic forms of obesity reported to date already manifesting in early childhood and persisting to adult life.^33^ We observed that rare variants in eight genes show concordant effects between SAC10 and adult BMI: two genes (*MC4R* and *CALCR*) are associated at exome-wide significance with both traits; and six genes (*INHBE*, *POMC, PTPRG, KIAA1109, OBSCN,* and *DIDO1*) show concordant effects across childhood and adult phenotypes with at least nominal significance (Table S2). Four of these genes (*CALCR, INHBE, MC4R,* and *POMC*) show apparent stronger effects on childhood adiposity (despite its weaker mode of assessment) than on adult BMI (Figure 6, Table S13).

One gene, *MADD*, identified for SAC10, appears to have specific effects on childhood adiposity with not even nominal association with adult BMI in either sex (Figure 6, Tables S2 and S13). *MADD* is also the only gene we identified in which loss-of-function confers lower adiposity. *MADD* is proximal to a reported common variant signal for fasting glucose^34^; that lead GWAS variant (rs7944584-A) is moderately correlated (R^2^ = 0.28) with the genome-wide significant common variant for SAC10 in our analysis (Figure S5C, Table S8) and is also an expression quantitative trait loci (eQTL) for *MADD* in several tissues.^35^ This eQTL association is consistent with the PTV association—the allele associated with lower *MADD* expression is associated with lower SAC10 and lower fasting glucose levels.

Conversely, two genes identified for adult BMI (*SLC12A5* and *SLTM*) appear to have adult-specific effects on adiposity (Figure 6, Tables S2 and S13). In a further subgroup analysis, their effects on adult BMI were not further modified by age at BMI measurement (Table S14).

Overall, apart from *OBSCN*, we observed no more than one or two individuals with homozygous or possible compound heterozygous rare PTV or DMG variants in any identified genes (Table S11). Therefore, the observed effect estimates reflect the effects in heterozygous variant carriers.

### Exploring DNA damage response processes in adiposity regulation

Several of the genes identified above (*MADD*, *DIDO1,* and *SLTM*) have been implicated in apoptosis,^36^^,^^37^^,^^38^ with *DIDO1* and *SLTM* also being linked to DNA damage.^39^^,^^40^ We explored further evidence for DNA damage response (DDR) processes in susceptibility to obesity by performing common variant genome-wide pathway enrichment analyses for SAC10 and adult BMI (STAR Methods). We observed enrichment for adult BMI (P_min_ = 3.0 × 10^−3^), but not SAC10, for two established DDR gene sets (“*Gene Ontology DNA repair*” and “*Gene Ontology Cellular response to DDR stimulus*”) and with a third custom-curated DDR gene set (Table S15). Furthermore, 38 genes in these DDR gene sets could be annotated as the nearest gene to a common variant signal for adult BMI (Table S16). Notable examples include *BRCA1* and *TP53,* which encode key DNA damage repair and checkpoint proteins^41^^,^^42^; *ALKBH3*, *ASCC3*, *FTO,* and *MGMT,* which are involved in the repair of DNA alkylation damage^43^^,^^44^^,^^45^; and *PRMT6*, *HUWE1,* and *NTHL1,* which are involved base excision repair.^46^^,^^47^^,^^48^ Genes encoding components of the Fanconi anemia pathway (such as *FANCD2*) have also been shown as critical for the regulation of adiposity, as well as genes involved in the cellular response to DNA damage via programmed cell death mechanisms (*BAD*, *BCL2*, and *RBBP6*).^49^^,^^50^^,^^51^^,^^52^

As DDR is implicated in biological aging,^53^ we tested whether DDR processes might be specific, or more relevant, to adult rather than childhood adiposity. To test this, we identified 843 common variant genome-wide significant signals for adult BMI and 349 GWAS signals for SAC10 in the UK Biobank. Of these, 114 signals were categorized as “adult-specific” (no effect on childhood adiposity-related traits) and 15 signals as “childhood-specific” (no association with adult BMI). The remaining 753 of 882 (85%) independent signals with complete look-up data were classified as “life-course-acting” (both childhood and adult effects) (Tables S16 and S17, STAR Methods).

We next mapped each GWAS signal to its closest gene, linking the 114 adult-acting signals to 112 genes, the 15 childhood-specific signals to 16 genes and the 753 life-course-acting signals to 708 genes (Tables S16 and S17, STAR Methods). We used these gene lists to perform gene-centric pathway analyses using STRING.^54^ No DDR pathway was significantly enriched among either the “adult-specific” or “childhood-specific” gene sets, whereas the “life-course-acting” genes showed enrichment for DDR and apoptosis processes, especially neuron death (*Wiki: “DNA damage response (only ATM dependent)”* (false discovery rate [FDR] = 0.011); *GO:BP: “Apoptotic process”* (FDR = 0.022) *GO:BP “Regulation of neuron death”* [FDR = 0.003]) (Table S18). The observed DDR effect could therefore not be attributable to a metabolic senescence phenotype that only begins in later life.

## Discussion

Here, we identify several genes in which rare, heterozygous loss-of-function confers a large effect on adult BMI either in men or women separately or affects recalled childhood adiposity. These findings highlight putative roles for DDR mechanisms in the etiology of obesity across the life-course, in addition to highlighting an intriguing pattern of adult-onset effects for some common and rare variants.

Our sex-stratified analysis of adult BMI identified rare loss-of-function variants in *DIDO1* and *SLC12A5*, which in this study confer higher risks of obesity than variants in the known monogenic causes of obesity, *MC4R* and *POMC*. However, it is unclear why their effects are specific to females. While rare variants in *DIDO1* also influenced free testosterone concentrations and/or WHRadjBMI specifically in females, these associations were weaker than those with BMI. *SLC12A5* encodes the potassium-chloride co-transporter, KCC2, which is highly expressed in the brain and moderately expressed in the pancreas,^55^ where it modulates calcium-dependent insulin secretion.^56^ Consistent with our observed sex-specific associations, female (but not male) mice heterozygous for *Slc12a5* gene deletions are reported to display impaired glucose tolerance^57^ (Table S19). However, the very low carrier count in males, which could be explained by strong selective constraint at *SLC12A5* (pLI = 1, o/e = 0.05 [0.02–0.14]) as assessed by gnomAD^58^ and which could indicate a deleterious effect on early life survival, makes it difficult to confidently conclude on an effect of rare loss-of-function variants in *SLC12A5* in males. We note that although the mouse model and common variant association at this locus are supportive for the *SLC12A5* rare variant association, strength of significance was inconsistent across a range of sensitivity analyses.

In our age-stratified analyses of SAC10 and adult BMI, most rare and common variants appear to influence obesity risk across the life-course. Eight of the 11 genes highlighted by ExWAS and 85% of the common genetic signals showed associations with both child and adult adiposity traits. Rare variants in only one gene, *MADD*, showed childhood-specific associations. *MADD* encodes an MAPK-activating protein^59^ with highest expression in the brain.^60^ Homozygous or compound heterozygous mutations in *MADD* underlie a multisystemic disorder (developmental delay with endocrine, exocrine, autonomic, and hematologic abnormalities [DEEAH syndrome]), characterized by poor weight gain, hypoglycaemia, and growth retardation.^61^^,^^62^ We found no association between *MADD* rare variant carrier status with any adult trait.

Rare loss-of-function variants in *MC4R* and *POMC* appear to have larger effects on adiposity in childhood than in adulthood. Rare functionally disrupting mutations in these genes are monogenic causes of severe early-onset obesity associated with uncontrolled appetite. Some case reports describe some attenuation with age in the hyperphagia that is typical of *MC4R* carriers.^63^ This could be explained by the previously reported physiological reduction in *POMC* expression with age,^64^^,^^65^ which might weaken the effect of loss-of-function variants. Alternatively, affected individuals might gradually develop more effective strategies to resist their appetitive drive to excess food intake and weight gain.

Emerging evidence indicates that the accretion of senescent cells is linked to metabolic disorders. Several cross-sectional studies have consistently related higher BMI to greater levels of DNA damage, chromosomal instability, and reduced DDR capacity,^66^^,^^67^^,^^68^^,^^69^ but with the hypothesis that obesity may induce DNA damage and limit DDR processes causing inflammation and oxidative stress. For example, previous research identified genetic determinants that predispose to obesity and also promote DNA damage.^70^^,^^71^ By contrast, our findings of selected genes highlighted by rare variants and of biological pathways enriched for common variant associations highlight neuron death, apoptosis, and DDR in the susceptibility to obesity risk across the life-course, rather than only being a downstream consequence.

DNA repair has been recognized as important in the regulation of adipocyte metabolism and senescence,^72^^,^^73^ with DNA damage in obese adipocytes thought to trigger p53-dependent signals, altering of adipocyte metabolism, and secretory function leading to adipose tissue senescence, inflammation, dysfunction, and insulin resistance. The elimination of these senescent adipocytes has been shown to alleviate adipose tissue inflammation and improve insulin resistance.^73^ Our findings suggest that disturbed DDR capacity previously associated with aging-related health outcomes could represent a potential marker of broader genomic instability and disease susceptibility, including obesity-related health outcomes. We found that DDR processes influence adiposity across the life-course, from childhood to adults, rather than increasing with age or being specific to late-onset adiposity. However, we acknowledge that most common variant signals for adiposity were categorized as “life-course acting” and we were likely underpowered to show effects on adult-onset adiposity.

One mechanism by which *DIDO1* variants may increase adiposity is by influencing cell cycle progression, and thus in enabling neuronal cell proliferation. The hypothalamus integrates signals from the periphery, and cells continue to proliferate in the adult hypothalamus to maintain energy homeostasis and enable metabolic flexibility.^74^ Local mitotic blockade in rodents leads to increased food intake, body weight, and adiposity.^75^ Furthermore, neurogenesis in the mouse hypothalamic arcuate nucleus is blocked in diet-induced obesity,^76^ suggesting that reduced cell proliferation might contribute to the impaired control of energy balance that leads to obesity. *DIDO1* has anti-apoptotic functions and is necessary for cell proliferation and survival in many types of cancer cells.^77^^,^^78^ Furthermore, *Dido1* regulates self-renewal of mouse embryonic stem cells.^79^ N-terminal truncation of DIDO3, the most widely expressed DIDO1 isoform, leads to aneuploidy, centrosome amplification, centromere-localized breaks, and chromosomal instability.^80^^,^^81^ Similarly, homozygous deletion of exon 16 of DIDO3 induces defects in RNA transcriptional termination, which contributes to genomic instability, DNA damage, and replication stress.^39^ Another gene product, SLTM, has been reported to localize to sites of DNA damage^40^ and has closely related family members with known DNA repair functions,^82^ suggesting it might also function in DDR and DNA repair pathways.

MADD acts as both an RAB3 guanine nucleotide exchange factor (GEF), and an RAB3 effector playing a role in formation and trafficking of synaptic vesicles. MADD-deficient fibroblasts display impaired exocytosis and increased susceptibility to activation of apoptosis pathways.^62^ As seen for *MADD*, *Dido* loss-of-function mice have neuro-developmental alterations.^83^ Previous studies have shown that genetic alterations leading to disrupted development in key regions of the brain required for energy homeostasis, such as the hypothalamus, are causative of obesity in humans.^84^ The neuro-developmental abnormalities reported in *Dido1* mutant mice may be related to the reported role of *Dido1* in regulating cilium length.^83^ Defects in genes required for ciliary function have been shown to cause obesity in humans and rodents.^85^ Interestingly, compound heterozygous mutations in *KIAA1109,* highlighted in our analysis for adult BMI in both sexes, have also been reported to affect cilia structural dynamics.^86^

### Limitations of study

We acknowledge several limitations of our study. Independent replication was restricted by the limited availability of similar large WES studies, although common variant associations at *CALCR, DIDO1, INHBE, MADD MC4R, POMC, SLC12A5,* and *SLTM* provide some confirmation that these genes are involved in adiposity etiology. Furthermore, these analyses were restricted to individuals of European ancestry, so their relevance to other populations is unclear. Last, our observation regarding a potential role of DNA damage in obesity etiology should be viewed as hypothesis generating, and we recognize that experimental studies will be required to confirm its biological relevance.

In conclusion, these findings highlight the importance of considering sex-specific and life-course effects in the genetic regulation of obesity. Our findings suggest that apoptosis and DDR, possibly through reduced neuron proliferation and greater neuron death, may contribute to obesity risk across the life-course. Further studies examining the roles of *MADD* and *DIDO1* in neuronal cells, both neurons and glial cells, may help to understand these mechanisms.

## STAR★Methods

### Key resources table

**Table:** 

REAGENT or RESOURCE	SOURCE	IDENTIFIER
**Deposited data**		

UK Biobank Data returns (to be submitted on publication)	UK Biobank	Application: 9905
UK Biobank phenotypic data	UK Biobank	Application: 9905
UK Biobank whole exome sequence data (450,000 release)	UK Biobank	Data field: 23148
Open Targets Genetics Platform	https://genetics.opentargets.org/	N/A
PhenoScanner	http://www.phenoscanner.medschl.cam.ac.uk/	N/A
UniProt	https://www.uniprot.org/	N/A
GPCRdb	https://gpcrdb.org/	N/A
IMPC (detailed in Table S19)	https://www.mousephenotype.org/	Accessed November 2022

**Software and algorithms**		

BOLT-LMM	https://alkesgroup.broadinstitute.org/BOLT-LMM/BOLT-LMM_manual.html	v2.3.6
STAAR	https://github.com/xihaoli/STAAR	v0.9.6
bcftools	https://github.com/samtools/bcftools	v1.14
MRC-Epid WES pipeline	https://github.com/mrcepid-rap/	N/A
plink	https://www.cog-genomics.org/plink/	v1.90b6.18
Variant Effect Predictor (VEP)	https://www.ensembl.org/info/docs/tools/vep/index.html	v104
LDSC	https://github.com/bulik/ldsc	v1.0.1
R	https://www.r-project.org/	v4.2.1
coloc R package	https://cran.r-project.org/web/packages/coloc/index.html	v5.1.0
sandwich R package	https://cran.r-project.org/web/packages/sandwich/index.html	v3.0-2
ordinal R package	https://cran.r-project.org/web/packages/ordinal/index.html	v2019.12–10
ggplot2 R package	https://cran.r-project.org/web/packages/ggplot2/index.html	v3.3.6
MAGMA	https://ctg.cncr.nl/software/magma	v1.09
GCTA	https://yanglab.westlake.edu.cn/software/gcta/#Overview	N/A
Locus zoom	https://locuszoom.org/	v1.4
plink	https://zzz.bwh.harvard.edu/plink/	v1.90.b6.18
STRING	https://string-db.org/	v11.5

### Resource availability

#### Lead contact

Further information and requests for resources should be directed to and will be fulfilled by the lead contact, John R.B. Perry (john.perry@mrc-epid.cam.ac.uk).

#### Materials availability

No materials were generated in this study.

### Method details

#### Exome-wide gene burden associations with BMI and SAC10

To identify genes associated with sex-stratified adult adiposity, we performed an ExWAS using WES data derived from 419,692 European genetic-ancestry UK Biobank participants (191,864 males and 227,828 females).^8^ As our outcome, we used adult BMI (kg/m^2^) from field 21001. Sex in our study was defined using the ‘genetic sex’ parameter by Bycroft et al.,^87^ and provided on UK Biobank field 22001. To identify genes associated with sex-combined childhood adiposity, we performed an ExWAS using WES data derived from 414,032 European genetic-ancestry UK Biobank participants (188,777 males and 225,255 females).^8^ As our outcome, we used SAC10 from field 1687, which is based on the question, “When you were 10 years old, compared to average would you describe yourself as thinner, plumper or about average?” and treated it as a continuous variable (0 = thinner, 1 = average, 2 = plumper). Although this phenotype is a proxy measure of childhood adiposity based on recalled data, it shows a strong genetic correlation with childhood BMI (r_g_ = 0.94)^25^ and only a moderate correlation with adult BMI (r_g_ = 0.55) as calculated with LDSC.^88^

Data processing and quality control were performed as described in Gardner et al.^89^ Individual gene burden tests were performed by collapsing exome variants according to their predicted functional consequence. We defined two functional categories of exome variants with a MAF<0.1% 1) high-confidence protein truncating variants (HC_PTV) and 2) damaging variants (DMG) which contain both high-confidence PTVs and missense variants as defined by a CADD score threshold of ≥25^23^. We defined Protein Truncating Variants (PTVs) as Variant Effect Predictor consequence of stop gained, frameshift, or splice acceptor/donor. To define ‘high-confidence’, we used the LOFTEE algorithm.^58^ We analyzed a maximum of 18,107 protein-coding genes with a minimum of >10 rare allele carriers in any of the tested categories. The burden association tests were conducted using BOLT-LMM.^90^ Our results are statistically well-calibrated as indicated by the absence of significant associations with synonymous variant burden (Figure S1, Table S1).

Sexual dimorphism was ascertained by comparing the association effect sizes between the male- and female-only analyses, as outlined below (where f denotes the female association summary statistics and m denotes the male ones)^91^:

$$z=\frac{\beta_{f}-\beta_{m}}{\sqrt{{se}_{f}^{2}+{se}_{m}^{2}}}$$

Associations were deemed dimorphic if their Bonferroni-corrected P value for the above z-statistic was <0.05 and the association did not reach nominal significance (≥0.05) in the opposite sex.

Carriers of genes highlighted in ExWAS were classified as homozygous carriers if they carried two copies of the same mutation and compound heterozygous if they carried two mutations in the same gene >5 basepairs apart (Table S11).

For all exome-wide significantly associated genes, the following further models were conducted.

##### Sensitivity analyses

Several sensitivity analyses were conducted to corroborate the identified associations. To validate our BOLT-LMM results, we additionally conducted burden association tests using STAAR^92^ as described in Gardner et al.,^89^ testing the same protein-coding transcripts as in our primary analyses. We also used an inverse-rank normalised BMI variable in the above-described BOLT-LMM framework to reduce the positive skew. We validated our associations by using linear models in R in the White-European unrelated subsample of UK Biobank for the equivalent discovery phenotypes and for T2D. To these models, we also applied heteroscedasticity-robust standard error calculations using the sandwich R package (3.0–2), to address case-control imbalances (Table S4). Furthermore, to test whether age at recruitment (field 21022) influenced BMI, we calculated the mean BMI of carriers of genes identified in the BMI ExWAS stratified by age (≥58 years and <58 years, with 58 years being the median age at recruitment for all UK Biobank participants). To determine a difference in means, we used the same formula as above^91^ and used a P value threshold of 0.05 (Table S14).

Finally, to ascertain whether the gene-level associations with DMG variants in *POMC* and MC4R might be driven by variants in known functional domains, we conducted domain-level burden tests (Table S9). To do this, variants were separated into the different functional domains within *POMC* using information from UniProt,^93^ while *MC4R* domains were also annotated using GPCRdb.^94^ Domain-level burden tests with sex-combined SAC10 were then performed using linear models, for domains that included at least 2 variants. We also tested known functionally implicated variants within these two genes (Table S10). To do this we used functionally validated loss- or gain-of function variants in *MC4R* reported by Lotta et al.,^26^ where 31/61 described variants were found in UK Biobank and in *POMC* by Shah et al.,^27^ where 15/1576 variants were found in UK Biobank.

##### Exome lookup in related metabolic traits

The exome-wide significantly associated genes were further tested for associations toward T2D risk, SHBG and free testosterone levels and WHRadjBMI within UK Biobank using BOLT-LMM, as described above (Table S5). For WHRadjBMI, waist-hip ratio was calculated using fields 48 and 49 and BMI from field 21001 from the first available instance where they were all available. For T2D, the phenotype was derived as described in Gardner et al..^89^ Using this trait, we performed logistic regressions in the unrelated white European subsample of UK Biobank to derive odds ratios (in R, v4.2.1). For SHBG, hormone levels were extracted from the first instance data of field 30830 and log-transformed, after removing participants taking hormone-influencing medications, including current reported use of HRT or oral contraception. For free testosterone, testosterone levels were extracted from the first instance data of field 30850 and the Vermeulen method was used in conjunction with data on SHBG, total testosterone levels and albumin (from field 30600) to calculate free testosterone levels. These were then log-transformed, after removing participants taking hormone-influencing medications. Finally, we tested for associations between genes identified in the female-only BMI analysis and a derived binary menopausal status phenotype, as described in Stankovic et al.,^95^ using linear models in the white-European unrelated subsample of the discovery cohort (Table S6). Interactions between menopause- and carrier-status for qualifying variants in these genes were also tested for BMI, using R.

##### Comparison of variance explained in childhood versus adult body size

To understand whether any of the exome-wide significantly associated genes may exert stronger effects in childhood than in adulthood or vice versa, we compared the variance explained across the two traits (BMI and SAC10) by being a carrier of qualifying mutations in any of the identified genes. Using R, BMI and SAC10 were first adjusted for the standard covariates (sex, age, age,^2^ exome-sequencing batch and the first 10 principal components) and the residual trait variance was tested against binary carrier status for each gene. The resulting model adjusted R^2^ was used as a scaled and comparable indication of the effect magnitude across the two outcomes.

##### Ordered logit models of obesity outcomesand carrier status of OBSCN

We conducted cumulative link models using childhood and adult obesity as ordered categorical outcomes, to quantify the relative risk of obesity conferred by carrying qualifying variants in any of the exome-wide significantly associated genes. To do this, we used the three levels of comparative size at age ten; “Thinner”, “Average”, “Plumper” and we similarly split adult BMI into three categories; BMI less than 20, BMI between 20 and 30, and BMI over 30. To estimate the effect of carrier status of *OBSCN* PTVs on SAC10, we used four levels; “homozygous”, “compound heterozygous”, “heterozygous”, and “non-carriers” (Table S12). Analyses were conducted using the “clm” function in the “ordinal” R package (v2019.12–10).

All data manipulations were conducted in R (v4.2.1) and plots were generated using ggplot2 (v3.3.6).

#### Common variant GWAS

##### GWAS signals proximal to the exome-identified genes

Common variant associations at the exome-identified genes were queried using the equivalent common variant GWAS (MAF>0.1%) in UK Biobank (adult BMI, N = 450,706, or SAC10, N = 444,345). Signal selection was performed as follows: genome-wide significant signals (p < 5 × 10^−8^) were initially selected based on proximity, in 1Mb windows. Secondary signals within these windows were then identified using the approximate conditional analysis in GCTA,^96^ using an LD reference panel derived from 25,000 participants of the UK Biobank study. Only secondary signals that were uncorrelated (R^2^<5%) with each other and did not exhibit an overt change in their association statistics between the baseline and conditional models (β changed by less than 20% or p value changed by less than four orders of magnitude) were kept. The lists of primary and secondary signals were further checked for pairwise LD within 10Mb windows, using plink (v1.90b6.18)^97^ and only independent signals (R^2^<5%) were kept, prioritising the distance-based ones in the case of linkage. The subsequent regions were plotted using LocusZoom (v1.4)^98^ and any identified GWAS signals were also queried in a GWAS meta-analysis of T2D.^99^

Signals were then annotated with their closest gene (within 500kb up- or downstream of the signal), using the NCBI RefSeq gene map for GRCh37 (via http://hgdownload.soe.ucsc.edu/goldenPath/hg19/database/). As most GWAS signals are intronic or intergenic, we overlayed these associations with other datasets to understand whether the GWAS variants can be causally linked to changes in the exome-identified genes' regulation. For genes with proximal GWAS signals, we calculated genomic windows of high linkage disequilibrium (LD; R^2^ > 0.8) for each given signal using plink and mapped these to the locations of known enhancers for the target genes, using the activity-by-contact (ABC) enhancer maps.^24^ Any seen overlaps indicate whether the genomic variants associating with the traits of interest directly changed the sequence of enhancers for the genes in question. We also performed colocalization analyses between the GWAS and eQTL data using the ABF function within the R package “coloc” (v5.1.0)^100^ and the cross-tissue meta-analysed GTEx eQTL data (V7, available via https://gtexportal.org and using the fixed-effects summary statistics).^35^ For this, variants within a 500kb window of each gene that were common between the GWAS and eQTL data were used and an H4 posterior probability (the probability of a single, shared causal variant) ≥0.75 was used as a colocalization threshold. Finally, outwith transcriptional changes, we performed a gene-level Multi-marker Analysis of GenoMic Annotation (MAGMA, v1.09) analysis,^101^ to collapse all observed genomic variants within each of the identified genes and calculate aggregate gene-level associations to the phenotypic traits. To do this, we specifically used common (MAF>0.1%) exonic variants within each gene (Table S8).

##### DDR pathway enrichment analyses

To ascertain the signal enrichment in genes related to DDR processes at the genome-wide level, we used the MAGMA gene-level associations as described above. We then collapsed this gene-level data into three pathways; GO cellular response to DNA damage stimulus (GO:0006974), GO DNA repair (GO:0006281) and an expert-curated broad DDR pathway (Table S20) and tested for enrichment against them under the MAGMA gene-set analysis functionality (Table S15).

##### Definition of GWAS signal trajectories

‘Adult-specific’ signals were defined as associated with adult BMI in UKBB with independent confirmation (p < 0.05) in GIANT consortium data^102^ but not associated (p>=0.05) with SAC10 and female pubertal timing (as measured by recalled age at menarche in UK Biobank) (which is sensitive to childhood adiposity^103^) and without a reported stronger association with a related lifestyle (e.g., alcohol consumption) or mental health trait (in PhenoScanner^104^^,^^105^ or Open Target Genetics^106^^,^^107^) (Tables S16 and S21). ‘Childhood-specific’ signals were defined as being associated with SAC10 in UK Biobank with independent confirmation (p < 0.05) in EGG consortium childhood BMI data^25^ and female pubertal timing (as measured by recalled age at menarche in UK Biobank) but not associated with adult BMI in UK Biobank (p>=0.05) (Table S17). Life-course-acting signals were defined as influencing both adult and childhood adiposity as measured adult BMI and SAC10 (p < 0.05). Furthermore, since a large number of BMI and SAC10 signals are expected to be the at the same locus, we only considered SAC10 signals that were independent of any BMI signal (R^2^ < 0.05) calculated as described above. For signals with missing data in the look-up GWAS, we identified proxies using an LD reference panel derived from 25,000 participants of the UK Biobank study (within 1 megabase of the reported signal and R^2^ > 0.6), choosing the variant with the highest R^2^ value.

We performed a gene-centric pathway analyses based on the closest gene for the ‘adult-specific’, ‘childhood-specific’ and ‘life-course-acting’ SNPs using STRING (https://string-db.org/).^54^ We tested for enrichment against all ‘Gene Ontology Biological Process (GO:BP)’ terms as well as KEGG, REACTOME and WikiPathway pathways. Any term with an adjusted p value <5% (Benjamini-Hochberg method) was considered to be statistically significantly (Table S18).

## Data Availability

Rare variant burden testing summary statistics are included in the supplemental information of this paper. Protected UK Biobank participant data will be returned to the UK Biobank resource and be accessible via application number 9905. This paper does not report original code. Any additional information required to reanalyse the data reported in this paper is available from the lead contact upon request.

## Supplemental information

Document S1. Figures S1–S5

Table S1. Genomic inflation factors for BMI ExWAS, related to STAR Methods Genomic inflation factor test statistics across different allele count ranges for synonymous, high-confidence protein truncating and damaging variants burden tests. Headers as follows: Phenotype, trait being tested; Mask, variant collapsing mask; MAC bin, allele count range; N, number of genes included; Lamda, genomic inflation test statistic.

Table S2. ExWAS results for sex-stratified BMI and sex-combined SAC10, related to Figures 1 and 4 ExWAS results for sex-stratified BMI and sex-combined SAC10. Two different masks (HC_PTV = high-confidence protein truncating variants and DMG = damaging variants) were tested. Headers as follows: Gene, gene symbol; Chr, chromosome; Start, start position of gene; End, end position of gene; Mask, variant collapsing mask; Discovery, indicates in which phenotype/mask combination each gene was discovered; BMI, body mass index with “_M” and “_F” indicating whether it was a male- or female-only analysis; SAC10, comparative body size age 10; BETA, effect size estimate; SE, standard error of the effect; P, p value for the association; LCI, lower 95% confidence interval of effect size estimate; UCI, upper 95% confidence interval of effect size estimate; AC, allele count; Sexual dimorphism, comparison of the sex-stratified effect size estimates using a two-sample t test where Z denotes the *Z* score and P_the the corresponding p-value.

Table S3. BMI and SAC10 individual rare variant look-up, related to Figures 1 and 4 Look-up of individual rare variants identified in the exome-wide significant BMI/SAC10 genes. Headers as follows: varID, variant ID; CHROM, chromosome; POS, position; REF, reference allele; ALT, alternative allele; AF, allele frequency; F_MISSING, fraction of individuals with missing genotype at this SNP; AN, allele number; AC, allele count; MANE, matched annotation between NCBI and EBI; ENST, Ensembl transcript ID; ENSG, Ensembl gene ID; SYMBOL, gene symbol; CSQ, mutation class; gnomAD_AF, gnomAD allele frequency; CADD, CADD score; REVEL, REVEL score; SIFT, SIFT classification on whether amino acid substitution affects protein function; PolyPhen, PolyPhen classification on whether amino acid substitution affects protein function; LOFTEE, loss-of-function transcript effect estimator, which identifies and labels loss-of-function variants as low-confidence (LC) or high-confidence (HC); PARSED_CSQ, mutation classification; AA, amino acid change; AApos, amino acid position; MULTI, multi-allelic; INDEL, insertion/deletion; MINOR, minor allele; MAJOR, major allele; BMI, body mass index with “_M” and “_F” indicating whether it was a male- or female-only analysis; SAC10, comparative body size age 10; BOLT_MAF, minor allele frequency as calculated from the BOLT pipeline; BETA, BOLT effect estimate; SE, BOLT standard error of effect estimate; P_BOLT_LMM_INF, BOLT p value estimate for the association; BOLT_AC, BOLT allele count.

Table S4. Sensitivity analyses, related to STAR Methods Sensitivity analyses to corroborate the identified associations in the discovery traits. Look-up of all BMI-associated genes in an analysis (BOLT) of inverse-rank normalized BMI and of all identified gene-trait combinations using STAAR and also linear regression models (excluding related participants), with the additional calculation of heteroscedasticity robust standards errors. Headers as follows: Gene, gene symbol; Mask, discovery variant collapsing mask; Trait, trait being tested; P, p value for the association; Carriers, number of carriers; BETA, effect size estimate; SE, standard error of the effect; N total, total number of participants with phenotypic and genotypic data available.

Table S5. Look-up of SAC10 and BMI genes for associations with other traits, related to STAR Methods Exome-identified SAC-associated genes were queried for associations with T2D, WHR (adjusted for BMI), and hand-grip strength, while BMI-associated genes were queried against sex-combined and stratified T2D, WHR (adjusted for BMI), SHBG, and free testosterone levels. Each outcome was tested using BOLT-LMM. For T2D, we also performed sensitivity analyses by logistic regression (excluding related participants), with the additional calculation of heteroscedasticity robust standards errors. Headers as follows: Gene, gene symbol; Mask, discovery variant collapsing mask; Trait, trait being tested; BETA, effect size estimate; SE, standard error of the effect; P, p value for the association; AC, allele count; N total, total number of participants with phenotypic and genotypic data available; Carriers, number of carriers; OR, exponential of the logOR (BETA) with UCI and LCI denoting the 95% confidence intervals of the OR.

Table S6. Menopause mediation, related to STAR Methods Genes identified in the female-only BMI analysis were queried for associations with binary menopausal status and for interactions between menopause and carrier status with the BMI outcome using linear regression models in the unrelated subsample of the discovery cohort. Headers as in Table S5.

Table S7. Effects of ExWAS-highlighted genes on categorical obesity traits, related to Figure 3 Comparative size at age 10; “Thinner”, “Average”, or “Plumper” was treated as an ordered categorical outcome to indicate "childhood obesity." Adult BMI was similarly split into three categories: <20, >20 but <30, and >30, to indicate "adult obesity." These two categorical outcomes were tested in cumulative link models against carrier status for qualifying rare exome variants, in the White-European unrelated subsample of the UK Biobank. SAC10-identified genes were tested against sex-combined child and adult obesity, while BMI-identified genes were tested against the equivalent sex-stratified traits as well. For each of these analyses headers are as follows: Gene, gene symbol; Mask, discovery variant collapsing mask; Trait, discovery associated trait with "_M" or "_F" indicating whether it was a male- or female-only analysis; AC, allele count; P, p value for the association; N total, total number of participants with phenotypic and genotypic data available; N carriers per level (1/2/3), number of carriers with qualifying variants for the gene-mask combination within each of the three body size categories; BETA (logOR), effect size estimate; SE, standard error of the effect; OR, exponential of the logOR (BETA) with UCI and LCI denoting the 95% confidence intervals of the OR.

Table S8. Common variant look-up for adult BMI or SAC10, T2D, and functional annotations, related to STAR Methods GWAS signals in the adult BMI or SAC10 GWAS were annotated on the basis of proximity to the exome-identified genes. These loci were further annotated with gene-level common coding variant associations, enhancer, and eQTL colocalization information and signals were looked-up in a GWAS of BMI excluding UK Biobank and a GWAS meta-analysis of T2D. Headers as follows: Gene, gene symbol; Trait, discovery associated trait; GWAS signal, rsID for independent signals mapping to gene in the equivalent trait GWAS; Chr, chromosome where signal is located; Pos, genomic position of signal in GRCh37; Distance from TSS/end, distance of GWAS signal to TSS/end of gene; A1, effect allele; A0, other allele; Freq1, effect allele frequency; Beta1, effect size estimate per copy of a1; SE, standard error of beta1; P, p value for the association (followed by p in the female-only and male-only analyses for the BMI signals); Proxy rsID, if GWAS signal was not available in this GWAS what proxy SNP was used; Proxy R2, linkage disequilibrium (LD) R-squared between GWAS signal and proxy rsID; Proxy A1, effect allele for proxy rsID; CodVar MAGMA nSNPs, number of common coding variants within gene that contributed to the gene-level association; P, p value for the gene-level association; ABC enhancers in LD SNP/R2, rsID and R-squared for the SNPs in high LD with GWAS signal that are within an enhancer of gene; ABC-max/tissue, the ABC-max score and the tissue where the enhancer interaction is observed; eQTL coloc (GTEx MA) h3.pp, posterior probability that there are different variants underlying the association pattern seen for levels of gene and variation in the phenotype; h4.pp, posterior probability that the same variants underlie both associations; Direction, if same variants underlying associations, what is the respective direction of effect between the expression and GWAS data.

Table S9. POMC and MC4R domain burden tests, related to Figure 5 The associations between *POMC*, *MC4R,* and SAC10 were further analyzed to ascertain the functional domain composition of the qualifying DMG variants. *POMC* domains were annotated using UniProt, and *MC4R* domains were further annotated using GPCRdb. DMG variants within each of the identified sub-gene domains were collapsed to calculate gene burden associations with SAC10. Functionally characterized variants within both genes were also extracted to perform burden associations. Headers as follows: Gene, gene symbol; Mask, variant collapsing mask; Trait, trait being tested; Carriers, number of carriers; N total, total number of participants with phenotypic and genotypic data available; N variants, number of variants included in mask; BETA, effect size estimate; SE, standard error of the effect; P, p value for the association.

Table S10. POMC and MC4R functional variants, related to STAR Methods Variant-level summary statistics for variants that contributed to the burden association from Table S9. Headers as follows: Function, assigned function of variants as defined in originating paper, under the categories LoF = LoF missense, GoF = GoF missense, LoF_PTV = LoF nonsense or frameshift, GoF & LoF = variant with opposing effects on the two functional pathways assessed, rest as seen in Table S3.

Table S11. Homozygous and compound heterozygous carriers of ExWAS-highlighted genes, related to STAR Methods Breakdown of the number of heterozygous, compound heterozygous, and homozygous carriers of genes highlighted in the ExWAS and their mean BMI/SAC10. Headers as follows: Gene, gene symbol; Trait, trait being tested; Mask, variant collapsing mask; Sex, which sex the look-up was performed in with “M” denoting male, “F” denoting female, and “both” denoting both sexes combined, Heterozygous, Homozygous, Compound heterozygous; N, number of individuals; Mean, average BMI or SAC10; SD, standard deviation of the mean.

Table S12. OBSCN effects on SAC10 by carrier status, related to STAR Methods Effect of carrier status of *OBSCN* high-confidence protein truncating variants on comparative body size age 10. This was assessed using a cumulative link model against carrier status in the White-European unrelated subsample of the UK Biobank. Headers as follows: Model, model being tested with “HOM” denoting homozygous carriers, “CHET” denoting compound heterozygous carriers, “HET” denoting heterozygous carriers and non-carriers; BETA (logOR/OR), effect size estimate; SE, standard error of effect size estimate; P, p value for the association; LCI(logOR/OR), lower 95% confidence interval of logOR/OR estimate; UCI(logOR/OR), upper 95% confidence interval of logOR/OR estimate.

Table S13. Variance in BMI vs. SAC10 explained by rare variant carrier status, related to Figure 6 For each identified exome gene, the variance explained by carrier status of qualifying rare exome variants in the residual variance in the outcome phenotype after adjusting for covariates, as adjusted model r-squared in the “discovery” trait-sex combinations. Headers as follows: Gene, gene symbol; Mask, variant collapsing mask in discovery analyses; Sex, sex used in discovery analyses; N BMI, total number of participants in BMI analysis; adj_rsq_bmi, model adjusted r-squared when testing residualized BMI against carrier status, latter two repeated for SAC10.

Table S14. Rare variant associations with adult BMI stratified by median age, related to STAR Methods Effect of genes identified in ExWAS on BMI stratified by age at recruitment. Headers as follows: Gene, gene symbol; Mean, average BMI; SE, standard error of the mean; Z, *Z* score derived from a two-sample t test comparing effect sizes between the two age groups; P_het, p value corresponding to *Z* score.

Table S15. Pathway enrichment using coding variant gene-level MAGMA associations, related to STAR Methods Selected DDR pathway enrichment in the sex-combined BMI and SAC10 GWAS. Genes included in each pathway can be found in Table S20. Headers as follows: N genes, number of genes included in this pathway with available association data; BETA, effect size estimate for the pathway level association; BETA_STD, semi-standardized BETA; SE, standard error of the effect size estimate (BETA); P, p value for the association.

Table S16. Classification of adult BMI GWAS signals as “adult-specific” or “life-course-acting,” related to STAR Methods Classification of adult BMI GWAS signals as “adult-specific” or “life-course-acting” using data on comparative body size age 10 (SAC10), age at menarche (AAM), and adult BMI from the GIANT consortium excluding UK Biobank data. Each SNP was annotated to its closest gene and appears twice if it was assigned more than one closest gene. Headers as follows: SNP, rsID for independent GWAS signal; CHR, chromosome; BP, base pair; ALLELE1, effect allele; ALLELE0, non-affect allele; A1FREQ, effect allele frequency; BETA, effect size estimate; SE, standard error of effect size estimate; P, p value for the association; Proxy, proxy SNP used if BMI SNP was not present in GIANT data; R2, R2 value for BMI SNP and proxy SNP; Adult-specific, whether a given SNP was classified as being adult-specific with “NA” denoting that SNP information was missing in at least one look-up dataset; Closest gene (±500 kb), closest gene to SNP; start, start position of gene; end, end position of gene; DDR gene, whether gene is appearing in “Gene Ontology DNA repair,” “Gene Ontology cellular response to DDR stimulus,” or a custom pathway curated by S.P.J.

Table S17. Classification of SAC10 GWAS signals as “childhood-specific” or “life-course-acting,” related to STAR Methods Classification of comparative body size age 10 (SAC10) GWAS signals as “childhood-specific” or “life-course-acting” using data on adult BMI (BMI), age at menarche (AAM), and childhood BMI from the EGG consortium. Each SNP was annotated to its closest gene and appears twice if it was assigned more than one closest gene. Headers as in Table S16 except: Proxy, proxy SNP used if SAC10 SNP was not present in EGG data; R2, R2 value for EGG SNP and proxy SNP; Childhood-specific, whether a given SNP was classified as being adult-specific with “NA” denoting that SNP information was missing in at least one look-up dataset; BMI signal, BMI signal linked (r2 >0.05) SAC10 signal; R2, r2 value of linked signals.

Table S18. STRING pathway enrichment pathway analysis of “adult-specific,” “childhood-specific,” and “life-course-acting” genes, related to STAR Methods STRING pathway enrichment pathway analysis of “adult-specific,” “childhood-specific,” and “life-course-acting” genes. No pathways passed the FDR correction for the “adult-specific” set of genes. Headers as follows: Term ID, term description; Term size, total number of proteins annotated with this term; Observed count, how many proteins in network that are annotated with this particular term; Strength, describes how large the enrichment effect is and is calculated as the ratio between (1) the number of proteins in your network that are annotated with a term and (2) the number of proteins that we expect to be annotated with this term in a random network of the same size; FDR, p value corrected for multiple testing using the Benjamini-Hochberg procedure; Matching proteins in network, symbols of all proteins present in your network for a given term.

Table S19. SLC12A5 glucose tolerance mouse data, related to STAR Methods Glucose tolerance data from IMPC, accessed via www.mousephenotype.org in November 2022. Analyzed as AUC (area under the curve) in male and female wild-type and heterozygote animals. Headers as follows: AUC group, name of group with accompanying AUC data; mean, mean AUC per group; SD, standard deviation of mean; N, number of samples analyzed per group; P, p value for the test comparing control and heterozygote animals.

Table S20. DDR pathways tested in MAGMA, related to STAR Methods Genes included in the selected DDR pathways in Table S15.

Table S21. PhEWAS of “adult-specific” BMI GWAS signals, related to STAR Methods PhEWAS of “adult-specific” BMI GWAS signals using Open Target Genetics and Phenoscanner. Headers as follows: SNP, rsID for independent BMI GWAS signal; CHR, chromosome; BP, base pair; Open Target genetics (p < 1 × 10^−5^); phenotypes associated with SNP at p < 1 × 10^−5^; Phenoscanner (p < 1 × 10^−5^), phenotypes associated with SNP at p < 1 × 10^−5^; Proxy, proxy SNP used when main SNP was not present in either database.

Table S22. Mean BMI per gene carrier group, related to Figure 2 Group mean BMI for carriers of qualifying variants in the exome-identified genes. Headers as follows: Gene, gene symbol; Mask, variant collapsing mask; N, number of carriers; Mean, mean BMI of carriers; SE, standard error of mean.

Document S2. Article plus supplemental information
