# Supplementary material for: Large-scale exome sequence analysis identifies sex- and age-specific determinants of obesity
Source: Cell Genom. 2023 Aug 2;3(8):100362. doi: 10.1016/j.xgen.2023.100362 (PMC10435378; doi:10.1016/j.xgen.2023.100362)
Supplement: Document S1. Figures S1–S5 [file mmc1.pdf]

Cell Genomics, Volume *3*

Supplemental information

Large-scale exome sequence analysis identifies sex- and age-specific determinants of obesity

Lena R. Kaisinger, Katherine A. Kentistou, Stasa Stankovic, Eugene J. Gardner, Felix R. Day, Yajie Zhao, Alexander Mörseburg, Christopher J. Carnie, Guido Zagnoli-Vieira, Fabio Puddu, Stephen P. Jackson, Stephen O'Rahilly, I. Sadaf Farooqi, Laura Dearden, Lucas C. Pantaleão, Susan E. Ozanne, Ken K. Ong, and John R.B. Perry

SUPPLEMENTAL INFORMATION

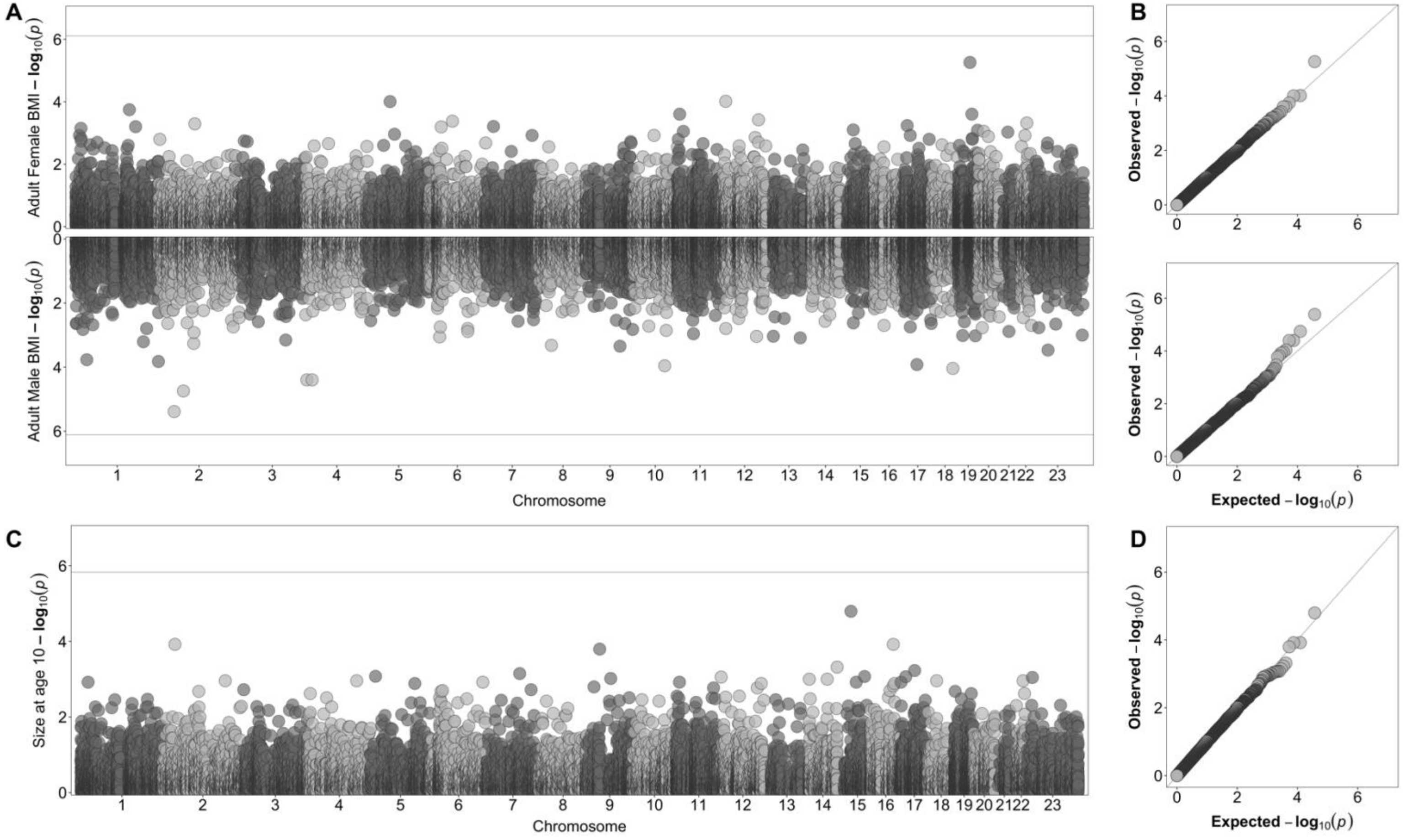

Figure S1 | Gene burden associations for synonymous rare variants with adult BMI and comparative body size at age 10, related to Figure 1 & 4. (A/C) Manhattan plot showing synonymous gene burden results for BMI and SAC10, respectively. The exome-wide significance thresholds used during discovery are indicated by the horizontal lines. (B/D) QQ plot of corresponding data.

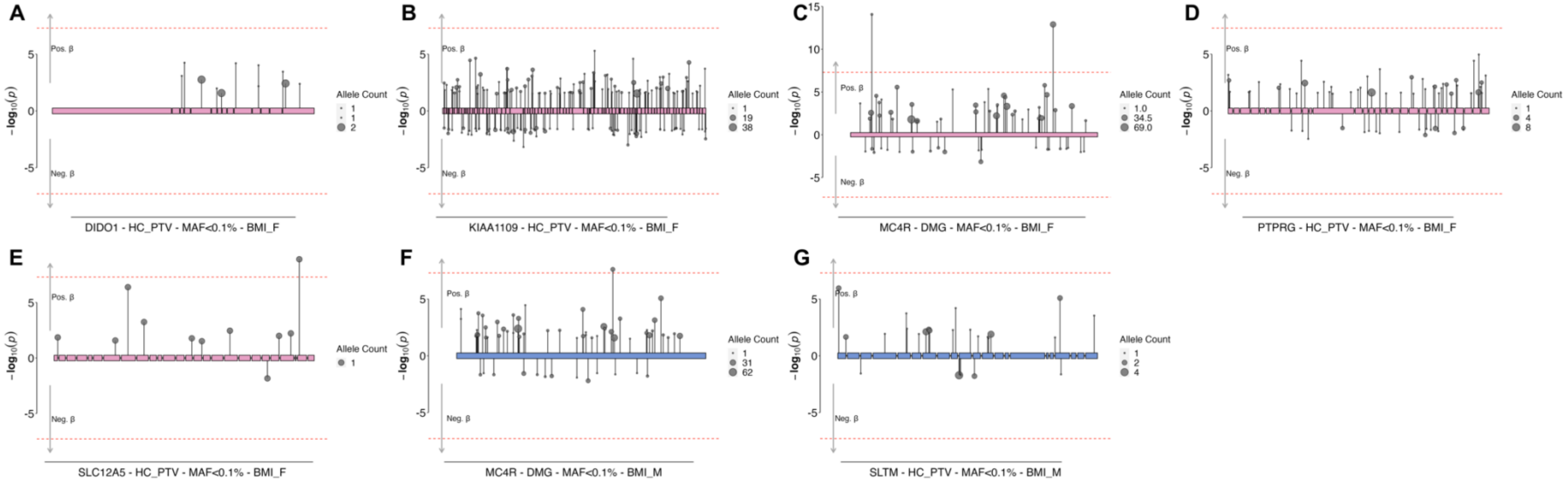

Figure S2 | Exome associations between the identified genes and female or male BMI in UK Biobank, related to Figure 1 & 2. Variant-level associations between variants within *DIDO1* (A), *KIAA1109* (B), *MC4R* (C), *PTPRG* (D), *SLC12A5* (E) and BMI in the women-only analysis and *MC4R* (F), *SLTM* (G) and BMI in the men-only analysis. Included variants from our discovery analyses had a minor allele frequency (MAF) <0.1% and were annotated to either be high-confidence protein truncating variants or missense variants with a high CADD score (≥25). Each variant is presented as an individual line extending to its association p-value (-log10), in the direction indicating the direction of effect on BMI in carriers of the alternate allele, while the point size indicates the number of carriers of each variant (i.e. allele count), as indicated in the figure legend. Extended data can be found in Table S3.

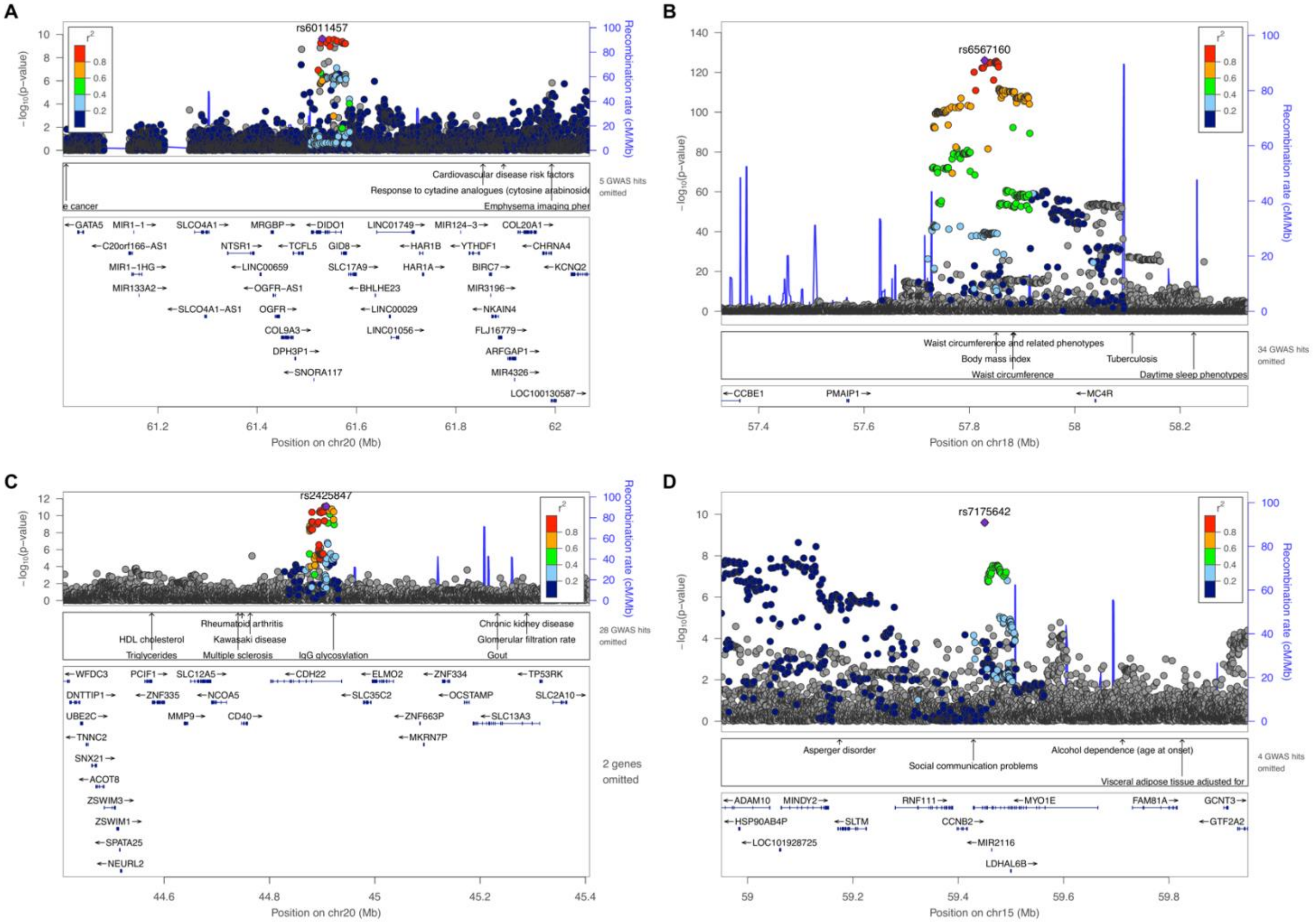

Figure S3 | Common variant associations with adult BMI, related to Figure 1. Locus zoom plots of BMI GWAS signals at the *DIDO1* (A), *MC4R* (B), *SLC12A5* (C) and *SLTM* (D) loci. SNPs are coloured in regard to their level of LD (R^2) towards the lead SNP, with SNPs in grey having an R^2<0.05. Underlying information can be found in Table S8.

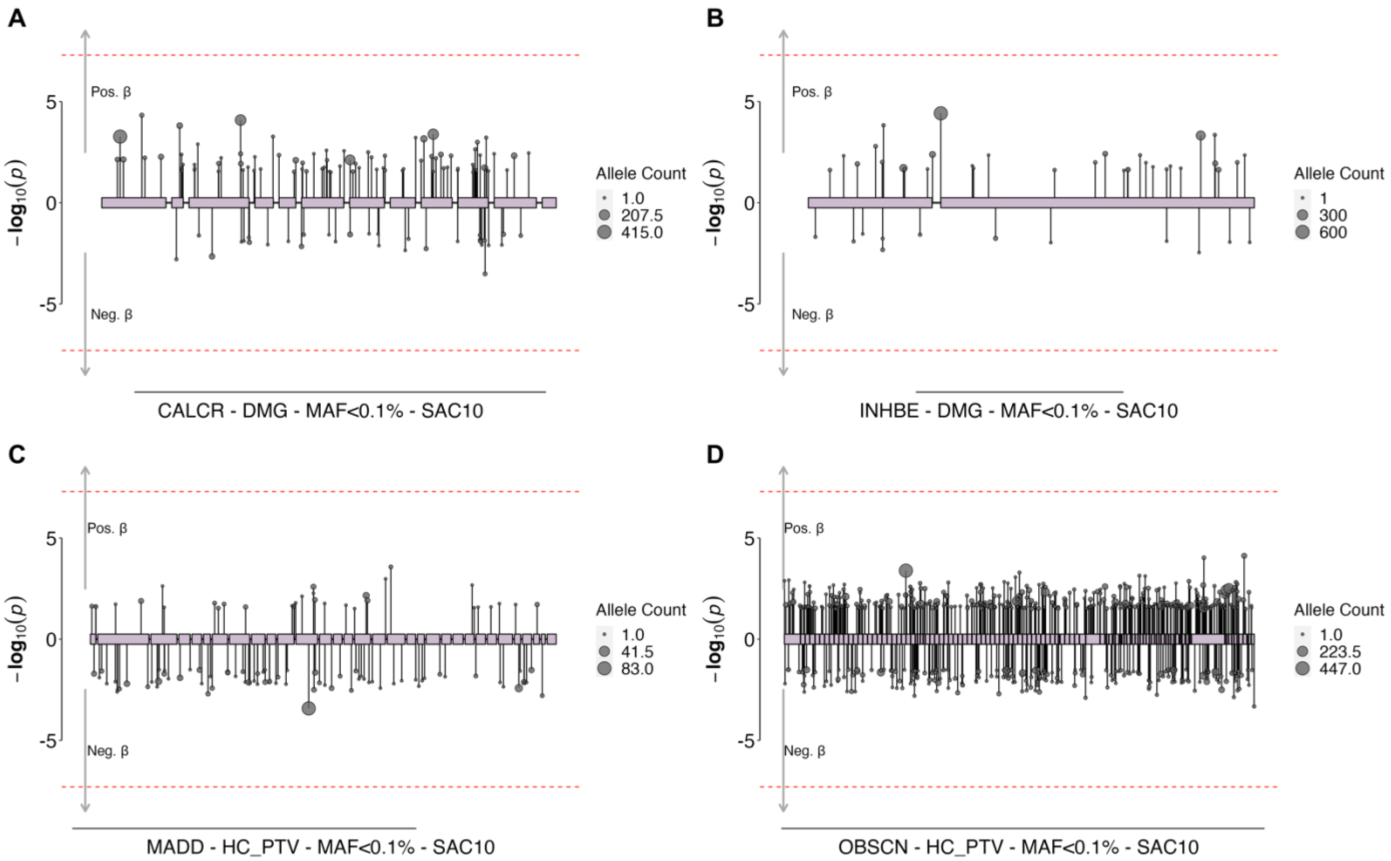

Figure S4 | Exome associations between the identified genes and sex-combined SAC10 in UK Biobank, related to Figure 4. Variant-level associations between variants within *CALCR* (A), *INHBE* (B), *MADD* (C), *OBSCN* (D) and SAC10. Included variants from our discovery analyses had a minor allele frequency (MAF) <0.1% and were annotated to either be high-confidence protein truncating variants or missense variants with a high CADD score (≥25). Each variant is presented as an individual line extending to its association p-value (-log10), in the direction indicating the direction of effect on SAC10 in carriers of the alternate allele, while the point size indicates the number of carriers of each variant (i.e. allele count), as indicated in the figure legend. Extended data can be found in Table S3.

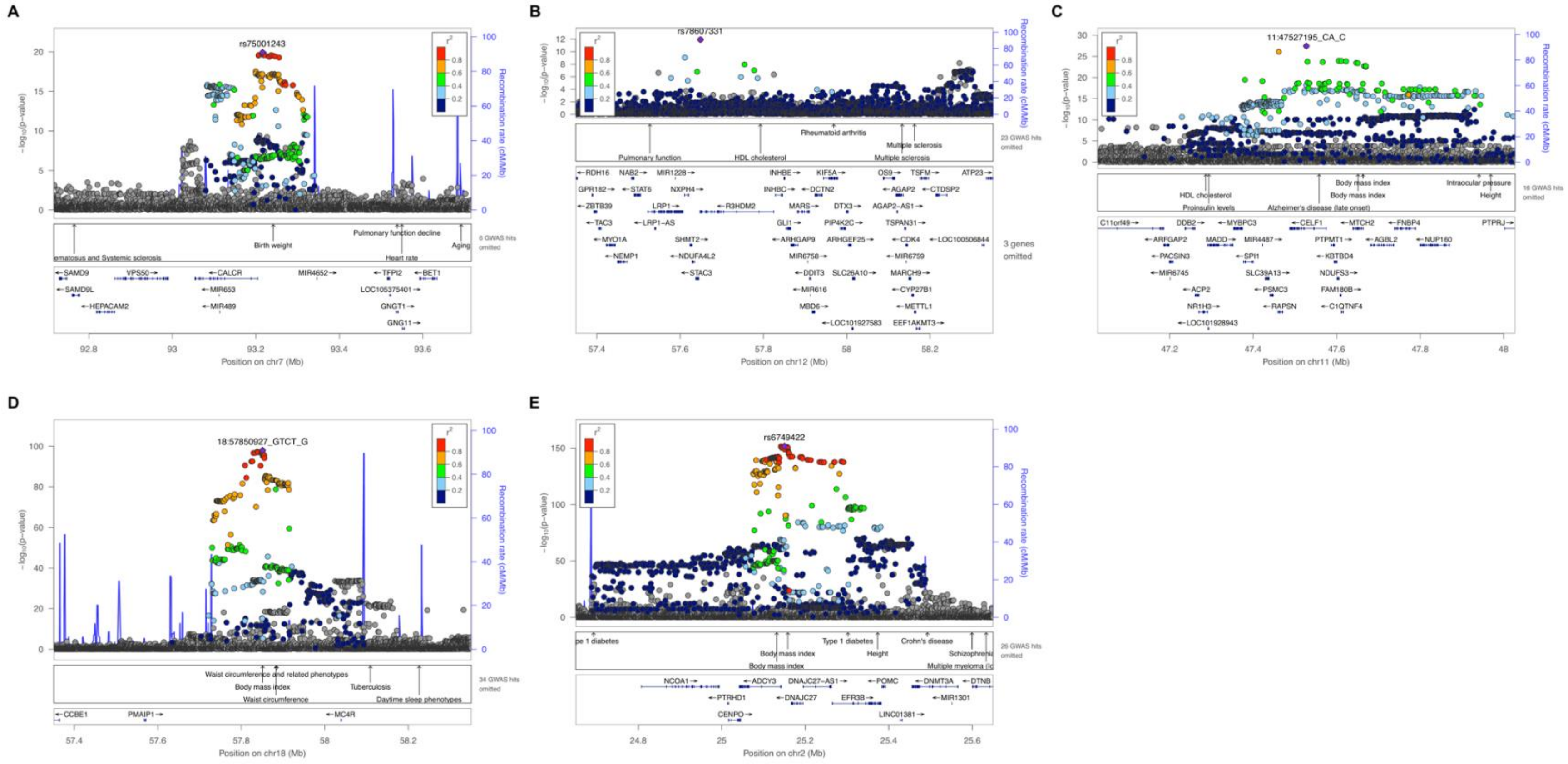

Figure S5 | Common variant associations with comparative body size at age 10, related to Figure 4. Locus zoom plots of the *CALCR* (A), *INHBE* (B), *MADD* (C), *MC4R* (D) and *POMC* (E) loci in the common variant SAC10 GWAS. Underlying data can be found in Table S8.
