# Supplementary material for: Large-scale exome sequence analysis identifies sex- and age-specific determinants of obesity
Source: Cell Genom. 2023 Aug 2;3(8):100362. doi: 10.1016/j.xgen.2023.100362 (PMC10435378; doi:10.1016/j.xgen.2023.100362)
Supplement: Document S2. Article plus supplemental information [file mmc24.pdf]

Cell Genomics

Article

Large-scale exome sequence analysis identifies sex- and age-specific determinants of obesity

Graphical abstract

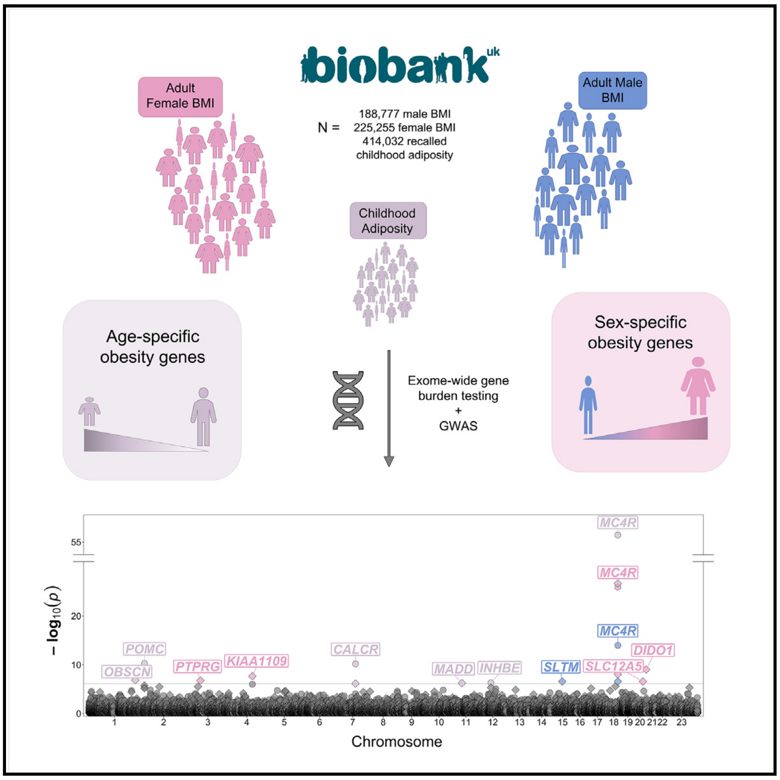

Authors

Lena R. Kaisinger, Katherine A. Kentistou, Stasa Stankovic, ..., Susan E. Ozanne, Ken K. Ong, John R.B. Perry

Correspondence

john.perry@mrc-epid.cam.ac.uk

In brief

Kaisinger et al. queried the genomes of ~420,000 individuals and identified genes associated with sex- and age-specific obesity risk. They highlight putative roles for DNA damage response mechanisms in obesity across the life-course, in addition to highlighting a pattern of adult-onset genetic effects.

Highlights

- Large-scale obesity exome sequence association study conducted in the UK Biobank
- Female-specific associations with rare variant burden in *DIDO1*, *PTPRG,* and *SLC12A5*
- Childhood-specific associations with rare variant burden in *MADD* and *OBSCN*
- Common variant analyses identify adult-onset obesity effects

Kaisinger et al., 2023, Cell Genomics *3*, 100362
August 9, 2023 © 2023 The Authors.
https://doi.org/10.1016/j.xgen.2023.100362

CellPress

Cell Genomics

CellPress OPEN ACCESS

Article

Large-scale exome sequence analysis identifies sex- and age-specific determinants of obesity

Lena R. Kaisinger,[1,6] Katherine A. Kentistou,[1,6] Stasa Stankovic,[1,6] Eugene J. Gardner,[1,5] Felix R. Day,[1] Yajie Zhao,[1] Alexander Mörseburg,[1,2] Christopher J. Carnie,[3,4] Guido Zagnoli-Vieira,[3] Fabio Puddu,[3] Stephen P. Jackson,[3,4] Stephen O'Rahilly,[2] I. Sadaf Farooqi,[2] Laura Dearden,[2] Lucas C. Pantaleão,[2] Susan E. Ozanne,[2] Ken K. Ong,[1,7] and John R.B. Perry[1,2,7,8,*]

[1]MRC Epidemiology Unit, Wellcome-MRC Institute of Metabolic Science, University of Cambridge, Cambridge CB2 0QQ, UK

[2]MRC Metabolic Diseases Unit, Wellcome-MRC Institute of Metabolic Science, University of Cambridge, Cambridge CB2 0QQ, UK

[3]Wellcome Trust/Cancer Research UK Gurdon Institute, Department of Biochemistry, University of Cambridge, Tennis Court Road, Cambridge CB2 1QR, UK

[4]Cancer Research UK Cambridge Institute, Li Ka Shing Building, University of Cambridge, Robinson Way, Cambridge CB2 0RE, UK

[5]Present address: Adrestia Therapeutics, Moneta Building 280, Babraham Research Campus, Cambridge CB22 3AT, UK

[6]These authors contributed equally

[7]Senior author

[8]Lead contact

*Correspondence: john.perry@mrc-epid.cam.ac.uk

https://doi.org/10.1016/j.xgen.2023.100362

SUMMARY

Obesity contributes substantially to the global burden of disease and has a significant heritable component. Recent large-scale exome sequencing studies identified several genes in which rare, protein-coding variants have large effects on adult body mass index (BMI). Here we extended such work by performing sex-stratified associations in the UK Biobank study (N∼420,000). We identified genes in which rare heterozygous loss-of-function increases adult BMI in women (*DIDO1, PTPRG,* and *SLC12A5*) and in men (*SLTM*), with effect sizes up to ∼8 kg/m^2. This is complemented by analyses implicating rare variants in *OBSCN* and *MADD* for recalled childhood adiposity. The known functions of these genes, as well as findings of common variant genome-wide pathway enrichment analyses, suggest a role for neuron death, apoptosis, and DNA damage response mechanisms in the susceptibility to obesity across the life-course. These findings highlight the importance of considering sex-specific and life-course effects in the genetic regulation of obesity.

INTRODUCTION

Obesity is a global issue affecting over 650 million adults and 124 million children and adolescents.[1] It is associated with increased mortality and morbidity as well as numerous comorbidities, such as cardiovascular disease and type 2 diabetes (T2D) and represents an enormous health burden. Obesity prevalence is greater in women than in men,[2] and women tend to have more body fat that is preferentially stored as subcutaneous fat in lower body depots, whereas men are more prone to visceral fat accumulation in the abdominal region.[3] These sex differences in adiposity affect risks for several obesity-related comorbidities, such as hypertension and T2D.[4] Yet, sex-specific analysis in research is uncommon, with most genetic studies adjusting for sex rather than analyzing data separately for men and women.

It is estimated that around 40%–70% of inter-individual variability in body mass index (BMI) can be attributed to genetic factors.[5,6] Very large population-based studies (N ∼700K) have identified over 900 genetic loci associated with BMI in adults.[7] Most of those genetic variants, although common, are located in non-coding regions, and collectively explain only ∼6% of the population variance in adult BMI.[7] The recent advent of whole-exome sequencing (WES) in large population-based studies[8] has enabled assessment of rare coding variants in disease and related traits. The largest WES analysis for BMI to date comprised ∼620,000 adults[9] and identified rare variants in 16 genes associated with adult BMI, including rare loss-of-function variants in *GPR75*, where 1:2,500 are heterozygous carriers and these have 1.8 kg/m^2 lower BMI and half the odds of obesity compared with non-carriers.

The genetic determinants of childhood adiposity are less well studied due to a relative paucity of data in large-scale childhood cohorts. However, childhood obesity has an important impact on child health, and individuals who develop obesity in childhood generally tend to remain obese as adults.[10] Studies of childhood BMI (combined sample size ∼56K) reported that many loci for adult BMI also operate in early life.[11,12] Furthermore, some loci exhibit stronger effects on adiposity in childhood, with less or even null effect in adulthood.[11] Across all these studies, the identified loci implicate brain-expressed genes, many acting on the

© 2023 The Authors.
This is an open access article under the CC BY license (http://creativecommons.org/licenses/by/4.0/).

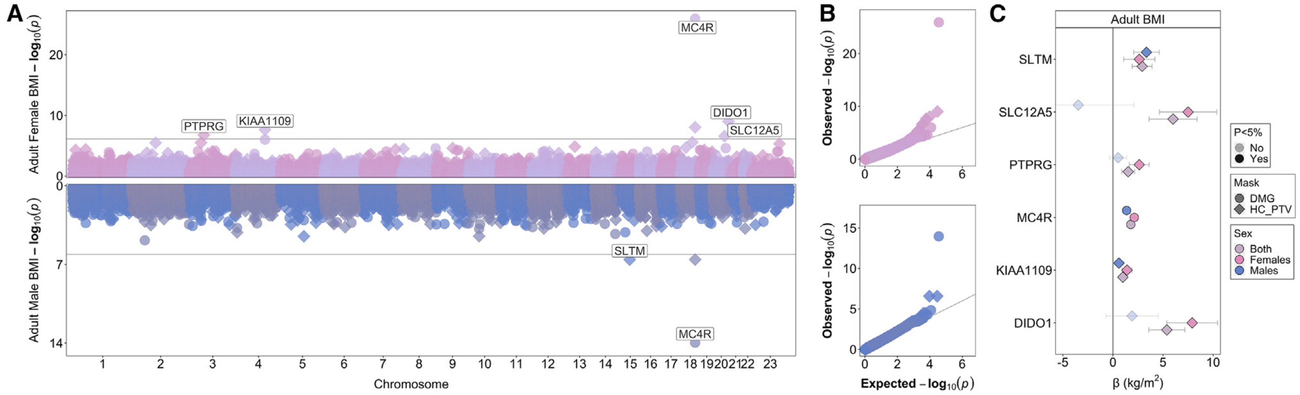

Figure 1. Gene burden associations of rare variants with adult BMI by sex

(A) Miami plot showing significantly associated genes (Bonferroni corrected $p < 7.76 \times 10^{-7}$) separately in women (upper) and men (lower).

(B) QQ plot of the same data.

(C) Effect estimates and 95% confidence intervals for each identified gene. For further details, see Tables S2 and S3.

leptin-melanocortin pathway, where rare heterozygous or homozygous loss-of-function of key genes are reported causes of monogenic obesity manifesting with hyperphagia in early childhood.[13–15] Furthermore, large-scale genetic studies of pubertal timing, an event closely coupled with childhood adiposity status, have also identified loci and biological mechanisms influencing early growth and development.[16–19]

Here, we explored two further approaches to identify genes that regulate susceptibility to obesity: rare coding variants (1) with sex-specific effects on adult BMI, or (2) associated with childhood adiposity, using a childhood body adiposity trait that was subjectively recalled in adults (sample size ~400K) from the UK Biobank study, and was recently reported to show high genetic correlation ($r_g = 0.85$) with objectively measured childhood BMI.[20] Sex-specific associations with body size and metabolic disease have been described for common genetic variation,[21,22] yet few examples exist for rarer variants, which offer greater opportunity to directly implicate causal genes. Likewise, common variant genome-wide association studies (GWASs) have been performed for recalled childhood adiposity, yet no similar study exists for rarer variants. To address this, we undertook a dual exome-wide association study (ExWAS) approach using data from up to 419,692 individuals from the UK Biobank study.

RESULTS

Rare variants associated with sex-stratified adult BMI

To identify rare coding variants that exhibit sex-specific effects on adult adiposity, we performed ExWAS for adult BMI (kg/m^2) separately in 191,864 men and 227,828 women from the UK Biobank study. Gene burden tests were performed by collapsing rare variants (minor allele frequency [MAF] < 0.1%) in individual genes according to two overlapping predicted functional categories: (1) high-confidence protein truncating variants (PTVs) and (2) PTV plus missense variants with a combined annotation dependent depletion (CADD)[23] score ≥25 (termed "damaging variants," DMG).The absence of significant signals (Figure S1) and inflation of test statistics (Table S1) across different allele count ranges for synonymous variant burden tests provided reassurance that our association testing models were well calibrated.

Five genes were associated with BMI in females (*DIDO1*, *KIAA1109*, *MC4R*, *PTPRG,* and *SLC12A5*) and two genes were associated with BMI in males (*MC4R* and *SLTM*) at exome-wide significance ($p < 7.76 \times 10^{-7}$; 0.05/64,396 tests (32,536 and 31,860 gene burden tests in females and males, respectively)) (Figures 1 and S2, Tables S2 and S3). Two of these genes, *MC4R* and *KIAA1109*, were reported in previous sex-combined ExWAS for BMI,[9] and showed exome-wide significant or subthreshold associations with BMI in both sexes, as did *SLTM* (men: beta = 3.34 kg/m^2/allele, $p = 2.7 \times 10^{-7}$, n = 38 PTV carriers; women: beta = 2.6, $p = 9.5 \times 10^{-4}$, n = 37 PTV carriers; $P_{sex\text{-}heterogeneity} = 0.48$).

Rare protein-coding variants in the remaining three genes, identified for BMI in females (*DIDO1*, *PTPRG,* and *SLC12A5*), have not previously been implicated in adiposity and appear to have female-specific effects, with not even nominal association with BMI in males; in females: *DIDO1* (beta = 7.91 kg/m^2, $p = 9.5 \times 10^{-10}$, n = 14 PTV carriers, $P_{sex\text{-}heterogeneity} = 1.2 \times 10^{-3}$), *PTPRG* (beta = 2.62 kg/m^2, $p = 1.7 \times 10^{-7}$, n = 92 PTV carriers, $P_{sex\text{-}heterogeneity} = 1.5 \times 10^{-3}$), and *SLC12A5* (beta = 7.50 kg/m^2, $p = 2.7 \times 10^{-7}$, n = 11 PTV carriers, $P_{sex\text{-}heterogeneity} = 5.8 \times 10^{-4}$) (Figures 1C, 2A, 2B, and S2A–S2E, Tables S2 and S3). We performed a number of sensitivity analyses to evaluate how robust these signals were to different analytical approaches (Table S4, STAR Methods). Test statistics were highly concordant for all reported genes, with the exception of *SLC12A5*. Plots along with association results for individual variants in the highlighted genes are shown in Figure S2 and Table S3.

To identify potential mechanisms underlying these observed female-specific effects, we further explored rare variant sex-stratified associations for *DIDO1*, *PTPRG,* and *SLC12A5* with free testosterone, sex-hormone binding globulin (SHBG), and waist-hip-ratio adjusted for BMI (WHRadjBMI). Female carriers of PTVs in *DIDO1* have a stronger association with circulating free

CellPress
OPEN ACCESS

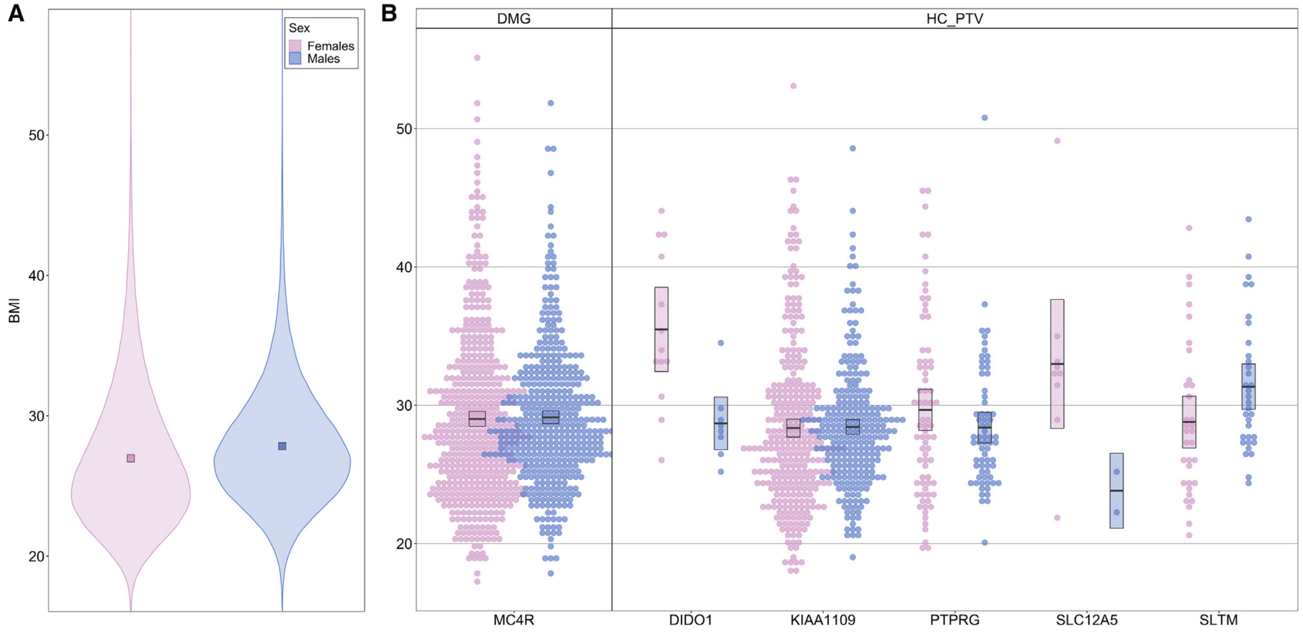

Figure 2. Distributions of adult BMI by sex

(A) In all UK Biobank participants; (B) among carriers of rare variants (DMG, damaging; PTV, protein truncating) in genes associated with sex-stratified BMI. Mean and 95% CI for each group are indicated by horizontal bars and boxes. Summarized group data can be found in Table S22.

testosterone concentrations (beta = 0.51, p = 9.8×10^{-3}) than their male counterparts (beta = 0.001, p = 0.99, $P_{sex\text{-}heterogeneity}$ = 2.7×10^{-2}) as well as with WHRadjBMI (females: beta = −0.04, p = 1.3×10^{-2}; males: beta = 0.02, p = 0.29; $P_{sex\text{-}heterogeneity}$ = 3.0×10^{-4}). Conversely, male carriers of PTVs in *PTPRG* have a stronger association with WHRadjBMI (beta = −0.02, p = 2.4×10^{-3}; $P_{sex\text{-}heterogeneity}$ = 8.8×10^{-3}) than their female counterparts (beta = −0.001, p = 0.92) (Table S5). Women carrying PTVs in *SLC12A5* had higher odds of T2D than non-carriers (odds ratio [OR] 17.1 [4.3–67.5], P_{glm} = 5.2×10^{-5}) with four of nine having T2D (UK Biobank T2D prevalence in females = 5.6% [12,675/227,363], P_{Exact} = 7.9×10^{-4}, Table S5). In contrast, we identified only two males (both non-obese and non-diabetic) carrying a PTV in *SLC12A5*. Unlike the *SLC12A5* BMI association, test statistics for this T2D association were consistent across sensitivity analyses (Table S5). None of the female-specific BMI-associated genes showed an association with menopausal status (Table S6).

The prevalence of obesity (BMI >30 kg/m^2) among carriers of DMG variants in *MC4R* was 39% (228 of 591) in females and 38% (195 of 518) in males, with ORs of 2.01 [1.68–2.41] and 1.71 [1.41–2.08], respectively (Figures 3A and 3C, Table S7). This is substantially lower than previously reported penetrance of *MC4R* variants that cause partial or complete loss-of-function *in vitro*.[13] By contrast, the prevalence of obesity among female carriers of PTV variants in *DIDO1* and *SLC12A5* was more than 80%, albeit there were relatively fewer carriers (12 of 14 and 9 of 11 carriers were obese, respectively) ($P_{Heterogeneity}$ = 9.9×10^{-6} and $P_{Heterogeneity}$ = 2.6×10^{-4}, respectively) (Table S7).

In the absence of sufficiently large ExWAS replication cohorts, we sought supporting evidence for our identified genes by examining independent common (MAF >0.1%) genetic variant (GWAS) associations with BMI. Four of our six identified ExWAS genes (*DIDO1*, *MC4R*, *SLC12A5,* and *SLTM*) mapped to within 500 kb of a common GWAS signal for sex-combined BMI (Figure S3, Table S8) and *DIDO1* and *MC4R* were also supported by gene-level associations between common non-synonymous variants and BMI (p = 3.8×10^{-5} and p = 5.0×10^{-10}, respectively). Furthermore, the lead GWAS SNP at the *DIDO1* locus (rs6011457, p = 2.4×10^{-10}) is intronic in *DIDO1*, is correlated with known enhancers for *DIDO1*,[24] and exhibits a stronger association with BMI in women (p = 3.2×10^{-8}) than BMI in men (p = 4.3×10^{-3}, P_{het} = 0.029). At the *SLTM* locus, we observed colocalization between common variant associations for BMI and *SLTM* expression (H4 posterior probability = 0.975, see STAR Methods), where variants that decrease *SLTM* expression increase BMI, which is directionally concordant with the rare variant association (Table S8).

Rare variants associated with childhood adiposity

We next undertook an ExWAS for childhood adiposity in 414,032 European genetic-ancestry adult UK Biobank study participants using the variable "comparative body size at age 10" (SAC10), which comprises responses to the question: "*When you were 10 years old, compared to average would you describe yourself as thinner, plumper, or about average?*" Although this is a recalled and non-quantitative indicator of childhood adiposity, it is reported to show strong genetic correlation with objectively measured childhood BMI (r_g = 0.85).[20] We confirmed this in data from a larger childhood sample (r_g = 0.94, N = 35,668),[25] and thus consider it to represent a robust trait for genetic analysis of childhood adiposity.

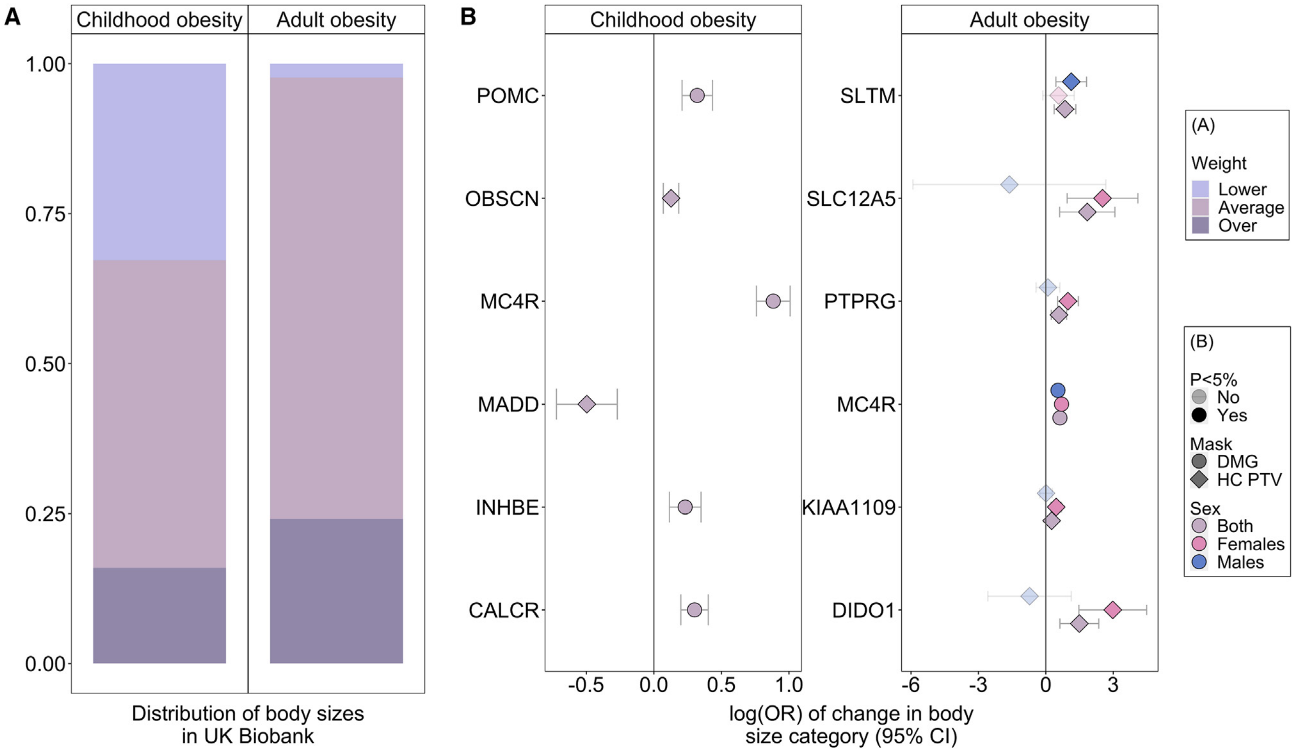

Figure 3. Adult and childhood obesity risk in carriers of rare damaging variants in the exome-identified genes

(A) Comparative size at age 10; "Thinner," "Average," or "Plumper" was treated as an ordered categorical outcome to indicate childhood obesity. Adult BMI was similarly split into three categories: <20, >20 but <30, and >30.

(B) These two categorical outcomes were tested in cumulative link models against carrier status for qualifying rare exome variants. Displayed log(OR) with 95% CIs and underlying data can be found in Table S7.

In a sex-combined ExWAS, six genes were associated with SAC10 (*CALCR*, *INHBE*, *MADD*, *MC4R*, *OBSCN,* and *POMC*) at exome-wide significance ($p < 1.47 \times 10^{-6}$, 0.05/34,127 tests) (Figures 4, 5, and S4, Tables S2 and S3). Two of these genes have been reported as disrupted in individuals with severe early-onset obesity[13,14]: *MC4R* (beta = 0.32, $p = 3.7 \times 10^{-57}$, N = 1,102 DMG carriers; OR 2.42 [2.14–2.74]) and *POMC* (beta = 0.12, $p = 5.6 \times 10^{-11}$, n = 1,303 DMG carriers (OR 1.38 [1.23–1.54]) (Figures 3A, 3B, 4C, and 5). Overall gene-level associations appeared to be driven by variants within specific subdomains, for *POMC* by variants that encode the α-MSH peptide, and for *MC4R* by variants within its intramembrane domains and particularly helix 1 and 4 (Figure 5, Table S9). We also observed concordant associations with previously reported gain- and loss-of-function variants in *MC4R*[26] as well as with gain-of-function variants in *POMC*[27] (Tables S9 and S10).

Two further genes have previously been implicated in adiposity phenotypes: *CALCR* (beta = 0.11, $p = 6.7 \times 10^{-11}$, n = 1,636 DMG carriers; OR 1.35 [1.22–1.50]) was reported in an ExWAS for adult BMI[9] and *INHBE* (beta = 0.10, $p = 5.0 \times 10^{-7}$, n = 1,199 DMG carriers; OR 1.26 [1.12–1.42]) was reported in an ExWAS for WHRadjBMI[28](Figures 3A, 3B, and 4C).

Rare variants in the two remaining genes associated with SAC10 have not previously been implicated in childhood adiposity or body size: *MADD* (beta = −0.18, $p = 5.9 \times 10^{-7}$, n = 327 PTV carriers) and *OBSCN* (beta = 0.05, $p = 1.4 \times 10^{-7}$, n = 4954 PTV carriers) (Figure 4C). Of the 4,954 individuals with a PTV in *OBSCN*, we identified one homozygous and 25 putative compound heterozygous individuals, who together had higher odds of being plumper as a child compared with non-carriers (OR = 2.45 [1.20–4.97], p = 0.013), which is substantially higher than the odds of heterozygous carriers compared with non-carriers (OR = 1.13 [1.07–1.20], $p = 3.0 \times 10^{-5}$) (Tables S11 and S12). *OBSCN* encodes one of three giant sarcomeric signaling proteins and is predominantly expressed in skeletal muscle[29] where it plays a role in the organization of myofibrils during assembly.[30] Biallelic loss-of-function variants have been identified in young and predominantly physically active individuals with rhabdomyolysis.[31] We additionally observed an association for heterozygous *OBSCN* mutations with greater measured hand-grip strength (0.58 kg ± 0.01, $p = 3.2 \times 10^{-9}$, n = 5,006 PTV carriers, Table S5), which might suggest a predominant effect on early muscle fiber development rather than adiposity.

We sought supporting evidence for our identified SAC10 ExWAS genes by assessing common genetic variant associations with SAC10 in the UK Biobank. Five of the six genes identified by ExWAS (*CALCR*, *INHBE*, *MADD*, *MC4R,* and *POMC*) map to within 500 kb of a common GWAS signal for SAC10 (Figure S5, Table S8). Furthermore, common non-synonymous

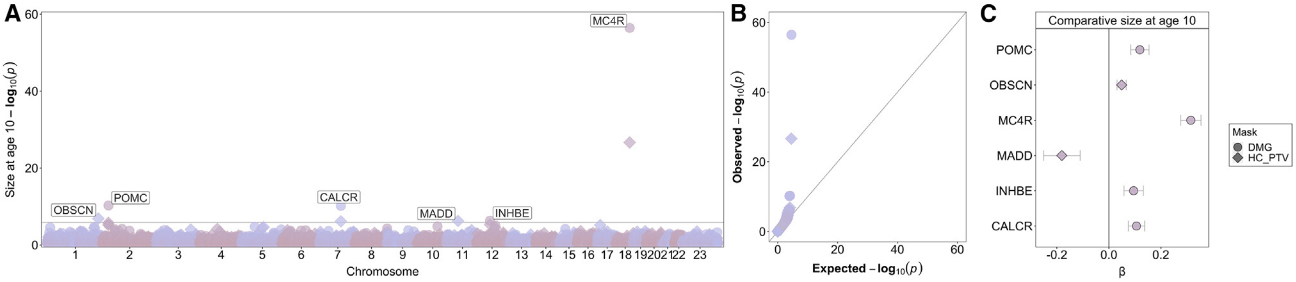

Figure 4. Gene burden associations of rare variants with comparative size at age 10

(A) Manhattan plot showing significantly associated genes (Bonferroni corrected $p < 1.47 \times 10^{-6}$).

(B) QQ plot of the same data.

(C) Effect estimates and 95% confidence intervals for each identified gene. For further details, see Tables S2 and S3.

variants in four of these genes (*CALCR*, *MADD*, *MC4R,* and *POMC*) showed gene-level associations with SAC10 (Table S8).

Comparison of rare variant associations between childhood adiposity and adult BMI

Previous work reported substantial overlap in common variant associations between childhood and adult BMI,[12,25,32] consistent with the strong tracking of childhood overweight into adulthood[10] with all monogenic forms of obesity reported to date already manifesting in early childhood and persisting to adult life.[33] We observed that rare variants in eight genes show concordant effects between SAC10 and adult BMI: two genes (*MC4R* and *CALCR*) are associated at exome-wide significance with both traits; and six genes (*INHBE*, *POMC, PTPRG, KIAA1109, OBSCN,* and *DIDO1*) show concordant effects across childhood and adult phenotypes with at least nominal significance (Table S2). Four of these genes (*CALCR, INHBE, MC4R,* and *POMC*) show apparent stronger effects on childhood adiposity (despite its weaker mode of assessment) than on adult BMI (Figure 6, Table S13).

One gene, *MADD*, identified for SAC10, appears to have specific effects on childhood adiposity with not even nominal association with adult BMI in either sex (Figure 6, Tables S2 and S13). *MADD* is also the only gene we identified in which loss-of-function confers lower adiposity. *MADD* is proximal to a reported common variant signal for fasting glucose[34]; that lead GWAS variant (rs7944584-A) is moderately correlated ($R^2 = 0.28$) with the genome-wide significant common variant for SAC10 in our analysis (Figure S5C, Table S8) and is also an expression quantitative trait loci (eQTL) for *MADD* in several tissues.[35] This eQTL association is consistent with the PTV association—the allele associated with lower *MADD* expression is associated with lower SAC10 and lower fasting glucose levels.

Conversely, two genes identified for adult BMI (*SLC12A5* and *SLTM*) appear to have adult-specific effects on adiposity (Figure 6, Tables S2 and S13). In a further subgroup analysis, their effects on adult BMI were not further modified by age at BMI measurement (Table S14).

Overall, apart from *OBSCN*, we observed no more than one or two individuals with homozygous or possible compound heterozygous rare PTV or DMG variants in any identified genes (Table S11). Therefore, the observed effect estimates reflect the effects in heterozygous variant carriers.

Exploring DNA damage response processes in adiposity regulation

Several of the genes identified above (*MADD*, *DIDO1,* and *SLTM*) have been implicated in apoptosis,[36–38] with *DIDO1* and *SLTM* also being linked to DNA damage.[39,40] We explored further evidence for DNA damage response (DDR) processes in susceptibility to obesity by performing common variant genome-wide pathway enrichment analyses for SAC10 and adult BMI (STAR Methods). We observed enrichment for adult BMI ($P_{min} = 3.0 \times 10^{-3}$), but not SAC10, for two established DDR gene sets (“*Gene Ontology DNA repair*” and “*Gene Ontology Cellular response to DDR stimulus*”) and with a third custom-curated DDR gene set (Table S15). Furthermore, 38 genes in these DDR gene sets could be annotated as the nearest gene to a common variant signal for adult BMI (Table S16). Notable examples include *BRCA1* and *TP53,* which encode key DNA damage repair and checkpoint proteins[41,42]; *ALKBH3*, *ASCC3*, *FTO,* and *MGMT,* which are involved in the repair of DNA alkylation damage[43–45]; and *PRMT6*, *HUWE1,* and *NTHL1,* which are involved base excision repair.[46–48] Genes encoding components of the Fanconi anemia pathway (such as *FANCD2*) have also been shown as critical for the regulation of adiposity, as well as genes involved in the cellular response to DNA damage via programmed cell death mechanisms (*BAD*, *BCL2*, and *RBBP6*).[49–52]

As DDR is implicated in biological aging,[53] we tested whether DDR processes might be specific, or more relevant, to adult rather than childhood adiposity. To test this, we identified 843 common variant genome-wide significant signals for adult BMI and 349 GWAS signals for SAC10 in the UK Biobank. Of these, 114 signals were categorized as “adult-specific” (no effect on childhood adiposity-related traits) and 15 signals as “childhood-specific” (no association with adult BMI). The remaining 753 of 882 (85%) independent signals with complete look-up data were classified as “life-course-acting” (both childhood and adult effects) (Tables S16 and S17, STAR Methods).

We next mapped each GWAS signal to its closest gene, linking the 114 adult-acting signals to 112 genes, the 15

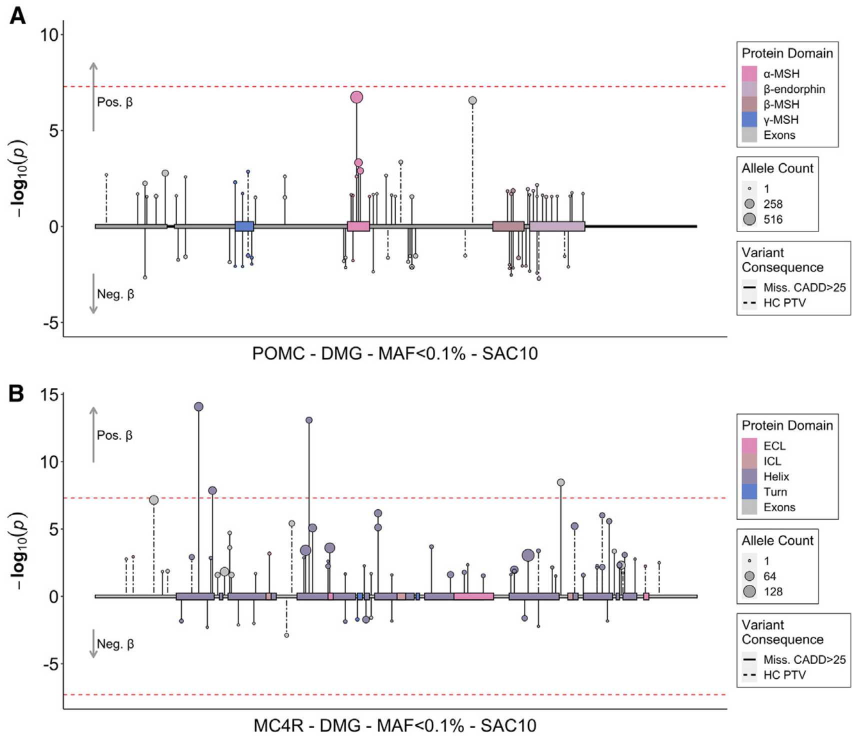

Figure 5. Exome associations between the functional domains of *POMC, MC4R* and SAC10 in the UK Biobank

Included variants in the *POMC* (A) and *MC4R* (B) genes from our discovery analyses had a minor allele frequency (MAF) smaller than 0.1% and were annotated to be either high-confidence protein truncating variants or missense variants with a high CADD score (≥25). Each variant is presented as an individual line extending to its association p value (−log10), in the direction indicating the direction of effect on SAC10 in carriers of the alternate allele, while the point size indicates the comparative number of carriers of each variant (i.e., allele count), as indicated in the figure legend. Domain-level association statistics can be found in Table S9.

childhood-specific signals to 16 genes and the 753 life-course-acting signals to 708 genes (Tables S16 and S17, STAR Methods). We used these gene lists to perform gene-centric pathway analyses using STRING.[54] No DDR pathway was significantly enriched among either the "adult-specific" or "childhood-specific" gene sets, whereas the "life-course-acting" genes showed enrichment for DDR and apoptosis processes, especially neuron death (*Wiki: "DNA damage response (only ATM dependent)"* (false discovery rate [FDR] = 0.011); *GO:BP: "Apoptotic process"* (FDR = 0.022) *GO:BP "Regulation of neuron death"* [FDR = 0.003]) (Table S18). The observed DDR effect could therefore not be attributable to a metabolic senescence phenotype that only begins in later life.

DISCUSSION

Here, we identify several genes in which rare, heterozygous loss-of-function confers a large effect on adult BMI either in men or women separately or affects recalled childhood adiposity. These findings highlight putative roles for DDR mechanisms in the etiology of obesity across the life-course, in addition to highlighting an intriguing pattern of adult-onset effects for some common and rare variants.

Our sex-stratified analysis of adult BMI identified rare loss-of-function variants in *DIDO1* and *SLC12A5*, which in this study confer higher risks of obesity than variants in the known monogenic causes of obesity, *MC4R* and *POMC*. However, it is unclear why their effects are specific to females. While rare variants in *DIDO1* also influenced free testosterone concentrations and/or WHRadjBMI specifically in females, these associations were weaker than those with BMI. *SLC12A5* encodes the potassium-chloride co-transporter, KCC2, which is highly expressed in the brain and moderately expressed in the pancreas,[55] where it modulates calcium-dependent insulin secretion.[56] Consistent with our observed sex-specific associations, female (but not male) mice heterozygous for *Slc12a5* gene deletions are reported to display impaired glucose tolerance[57] (Table S19). However, the very low carrier count in males, which could be explained by strong selective constraint at *SLC12A5* (pLI = 1, o/e = 0.05 [0.02–0.14]) as assessed by gnomAD[58] and which could indicate a deleterious effect on early life survival, makes it difficult to confidently conclude on an effect of rare loss-of-function variants in *SLC12A5* in males. We note that although the mouse model and common variant association at this locus are supportive for the *SLC12A5* rare variant association, strength of significance was inconsistent across a range of sensitivity analyses.

In our age-stratified analyses of SAC10 and adult BMI, most rare and common variants appear to influence obesity risk across the life-course. Eight of the 11 genes highlighted by ExWAS and 85% of the common genetic signals showed associations with both child and adult adiposity traits. Rare variants in only one gene, *MADD*, showed childhood-specific associations. *MADD* encodes an MAPK-activating protein[59] with highest expression in the brain.[60] Homozygous or compound heterozygous mutations in *MADD* underlie a multisystemic disorder (developmental delay with endocrine, exocrine, autonomic, and hematologic abnormalities [DEEAH syndrome]), characterized by poor weight gain, hypoglycaemia, and growth retardation.[61,62] We found no association between *MADD* rare variant carrier status with any adult trait.

Rare loss-of-function variants in *MC4R* and *POMC* appear to have larger effects on adiposity in childhood than in adulthood. Rare functionally disrupting mutations in these genes are monogenic causes of severe early-onset obesity associated with uncontrolled appetite. Some case reports describe some attenuation with age in the hyperphagia that is typical of *MC4R* carriers.[63] This could be explained by the previously reported

A

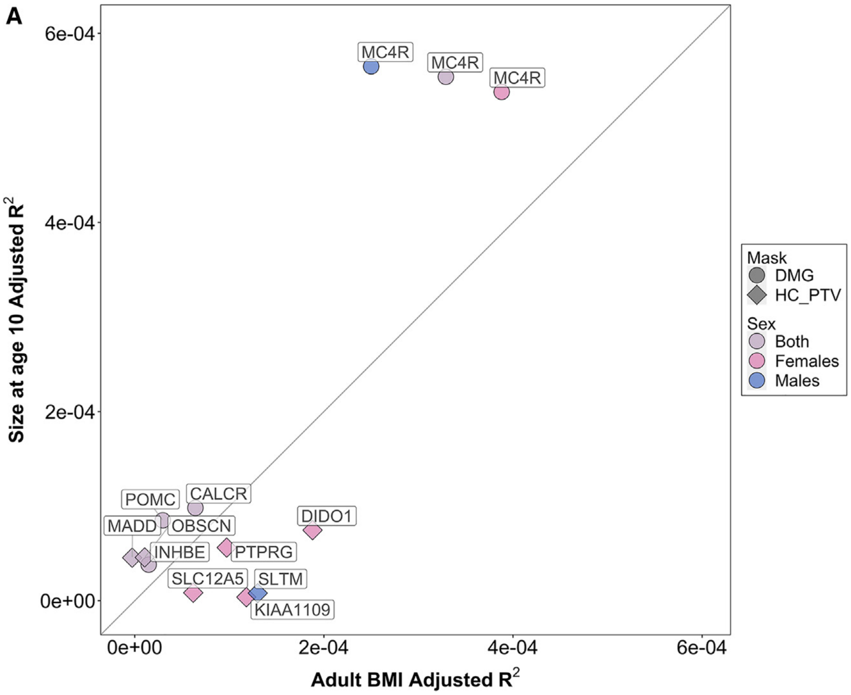

Figure 6. Comparison of rare variant gene-level effects on adult BMI and comparative size at age 10

For each identified exome gene, the adjusted R^2 for carrier status of qualifying rare exome variants against residual variance in the outcome phenotype after adjusting for covariates. For each gene, the "discovery" trait-sex combinations are shown. Underlying data can be found in Table S13.

physiological reduction in *POMC* expression with age,[64,65] which might weaken the effect of loss-of-function variants. Alternatively, affected individuals might gradually develop more effective strategies to resist their appetitive drive to excess food intake and weight gain.

Emerging evidence indicates that the accretion of senescent cells is linked to metabolic disorders. Several cross-sectional studies have consistently related higher BMI to greater levels of DNA damage, chromosomal instability, and reduced DDR capacity,[66–69] but with the hypothesis that obesity may induce DNA damage and limit DDR processes causing inflammation and oxidative stress. For example, previous research identified genetic determinants that predispose to obesity and also promote DNA damage.[70,71] By contrast, our findings of selected genes highlighted by rare variants and of biological pathways enriched for common variant associations highlight neuron death, apoptosis, and DDR in the susceptibility to obesity risk across the life-course, rather than only being a downstream consequence.

DNA repair has been recognized as important in the regulation of adipocyte metabolism and senescence,[72,73] with DNA damage in obese adipocytes thought to trigger p53-dependent signals, altering of adipocyte metabolism, and secretory function leading to adipose tissue senescence, inflammation, dysfunction, and insulin resistance. The elimination of these senescent adipocytes has been shown to alleviate adipose tissue inflammation and improve insulin resistance.[73] Our findings suggest that disturbed DDR capacity previously associated with aging-related health outcomes could represent a potential marker of broader genomic instability and disease susceptibility, including obesity-related health outcomes. We found that DDR processes influence adiposity across the life-course, from childhood to adults, rather than increasing with age or being specific to late-onset adiposity. However, we acknowledge that most common variant signals for adiposity were categorized as "life-course acting" and we were likely underpowered to show effects on adult-onset adiposity.

One mechanism by which *DIDO1* variants may increase adiposity is by influencing cell cycle progression, and thus in enabling neuronal cell proliferation. The hypothalamus integrates signals from the periphery, and cells continue to proliferate in the adult hypothalamus to maintain energy homeostasis and enable metabolic flexibility.[74] Local mitotic blockade in rodents leads to increased food intake, body weight, and adiposity.[75] Furthermore, neurogenesis in the mouse hypothalamic arcuate nucleus is blocked in diet-induced obesity,[76] suggesting that reduced cell proliferation might contribute to the impaired control of energy balance that leads to obesity. *DIDO1* has anti-apoptotic functions and is necessary for cell proliferation and survival in many types of cancer cells.[77,78] Furthermore, *Dido1* regulates self-renewal of mouse embryonic stem cells.[79] N-terminal truncation of DIDO3, the most widely expressed DIDO1 isoform, leads to aneuploidy, centrosome amplification, centromere-localized breaks, and chromosomal instability.[80,81] Similarly, homozygous deletion of exon 16 of DIDO3 induces defects in RNA transcriptional termination, which contributes to genomic instability, DNA damage, and replication stress.[39] Another gene product, SLTM, has been reported to localize to sites of DNA damage[40] and has closely related family members with known DNA repair functions,[82] suggesting it might also function in DDR and DNA repair pathways.

MADD acts as both an RAB3 guanine nucleotide exchange factor (GEF), and an RAB3 effector playing a role in formation and trafficking of synaptic vesicles. MADD-deficient fibroblasts display impaired exocytosis and increased susceptibility to activation of apoptosis pathways.[62] As seen for *MADD*, *Dido* loss-of-function mice have neuro-developmental alterations.[83] Previous studies have shown that genetic alterations leading to disrupted development in key regions of the brain required for energy homeostasis, such as the hypothalamus, are causative of obesity in humans.[84] The neuro-developmental abnormalities reported in *Dido1* mutant mice may be related to the reported role of *Dido1* in regulating cilium length.[83] Defects in genes required for ciliary function have been shown to cause obesity

in humans and rodents.[85] Interestingly, compound heterozygous mutations in *KIAA1109,* highlighted in our analysis for adult BMI in both sexes, have also been reported to affect cilia structural dynamics.[86]

Limitations of study

We acknowledge several limitations of our study. Independent replication was restricted by the limited availability of similar large WES studies, although common variant associations at *CALCR, DIDO1, INHBE, MADD MC4R, POMC, SLC12A5,* and *SLTM* provide some confirmation that these genes are involved in adiposity etiology. Furthermore, these analyses were restricted to individuals of European ancestry, so their relevance to other populations is unclear. Last, our observation regarding a potential role of DNA damage in obesity etiology should be viewed as hypothesis generating, and we recognize that experimental studies will be required to confirm its biological relevance.

In conclusion, these findings highlight the importance of considering sex-specific and life-course effects in the genetic regulation of obesity. Our findings suggest that apoptosis and DDR, possibly through reduced neuron proliferation and greater neuron death, may contribute to obesity risk across the life-course. Further studies examining the roles of *MADD* and *DIDO1* in neuronal cells, both neurons and glial cells, may help to understand these mechanisms.

STAR★METHODS

Detailed methods are provided in the online version of this paper and include the following:

- KEY RESOURCES TABLE
- RESOURCE AVAILABILITY
  - Lead contact
  - Materials availability
  - Data and code availability
- METHOD DETAILS
  - Exome-wide gene burden associations with BMI and SAC10
  - Common variant GWAS

SUPPLEMENTAL INFORMATION

Supplemental information can be found online at https://doi.org/10.1016/j.xgen.2023.100362.

ACKNOWLEDGMENTS

This work was funded by the Medical Research Council (Unit programs: MC_UU_12015/2, MC_UU_00006/2, MC_UU_00014/4). L.D. is a Royal Society Dorothy Hodgkin Fellow. Research in the S.P.J. lab is funded by Cancer Research UK Discovery Grant (DRCPGM\100005), CRUK RadNet Cambridge (C17918/A28870), ERC Synergy grant DDREAMM (855741), and core funding was provided by CRUK Cambridge Institute (A:29580). This project has received funding from CRUK DRCPGM\100005 and C6/A18796 (C.C. and F.P.) and Wellcome Investigator Award 206388/Z/17/Z (G.Z.V.). Gurdon Institute core infrastructure funding was provided by Cancer Research UK (C6946/A24843) and Wellcome (WT203144).

For open access, the author has applied a Creative Commons Attribution (CC BY) public copyright license to any author accepted manuscript version arising from this submission. This research was conducted using the UK Biobank resource under application 9905.

AUTHOR CONTRIBUTIONS

L.R.K., K.A.K., S.S., E.J.G., F.R.D., Y.Z., and A.M. performed analyses. F.R.D., L.P., C.J.C., I.S.F., S.P.J., G.Z.V., F.P., L.D., S.O., and S.E.O. contributed to interpretation and feedback. K.K.O., J.R.B.P., S.P.J., S.O., and S.E.O. designed the study. L.R.K., K.A.K., S.S., L.D., K.K.O., and J.R.B.P. drafted the manuscript. All authors reviewed the manuscript.

DECLARATION OF INTERESTS

E.J.G., S.P.J., and J.R.B.P. are employees and shareholders of Adrestia Therapeutics Ltd.

Received: December 8, 2022
Revised: March 15, 2023
Accepted: July 3, 2023
Published: August 2, 2023

REFERENCES

1. WHO. Obesity and overweight. https://www.who.int/news-room/fact-sheets/detail/obesity-and-overweight.
2. Flegal, K.M., Kruszon-Moran, D., Carroll, M.D., Fryar, C.D., and Ogden, C.L. (2016). Trends in Obesity Among Adults in the United States, 2005 to 2014. JAMA *315*, 2284–2291. https://doi.org/10.1001/jama.2016.6458.
3. Karastergiou, K., Smith, S.R., Greenberg, A.S., and Fried, S.K. (2012). Sex differences in human adipose tissues - the biology of pear shape. Biol. Sex Differ. *3*, 13. https://doi.org/10.1186/2042-6410-3-13.
4. Goossens, G.H., Jocken, J.W.E., and Blaak, E.E. (2021). Sexual dimorphism in cardiometabolic health: the role of adipose tissue, muscle and liver. Nat. Rev. Endocrinol. *17*, 47–66. https://doi.org/10.1038/s41574-020-00431-8.
5. Silventoinen, K., Jelenkovic, A., Sund, R., Hur, Y.M., Yokoyama, Y., Honda, C., Hjelmborg, J.v., Möller, S., Ooki, S., Aaltonen, S., et al. (2016). Genetic and environmental effects on body mass index from infancy to the onset of adulthood: an individual-based pooled analysis of 45 twin cohorts participating in the COllaborative project of Development of Anthropometrical measures in Twins (CODATwins) study. Am. J. Clin. Nutr. *104*, 371–379. https://doi.org/10.3945/ajcn.116.130252.
6. Silventoinen, K., Jelenkovic, A., Sund, R., Yokoyama, Y., Hur, Y.M., Cozen, W., Hwang, A.E., Mack, T.M., Honda, C., Inui, F., et al. (2017). Differences in genetic and environmental variation in adult BMI by sex, age, time period, and region: an individual-based pooled analysis of 40 twin cohorts. Am. J. Clin. Nutr. *106*, 457–466. https://doi.org/10.3945/ajcn.117.153643.
7. Yengo, L., Sidorenko, J., Kemper, K.E., Zheng, Z., Wood, A.R., Weedon, M.N., Frayling, T.M., Hirschhorn, J., Yang, J., and Visscher, P.M.; GIANT Consortium (2018). Meta-analysis of genome-wide association studies for height and body mass index in approximately 700000 individuals of European ancestry. Hum. Mol. Genet. *27*, 3641–3649. https://doi.org/10.1093/hmg/ddy271.
8. Backman, J.D., Li, A.H., Marcketta, A., Sun, D., Mbatchou, J., Kessler, M.D., Benner, C., Liu, D., Locke, A.E., Balasubramanian, S., et al. (2021). Exome sequencing and analysis of 454,787 UK Biobank participants. Nature *599*, 628–634. https://doi.org/10.1038/s41586-021-04103-z.
9. Akbari, P., Gilani, A., Sosina, O., Kosmicki, J.A., Khrimian, L., Fang, Y.Y., Persaud, T., Garcia, V., Sun, D., Li, A., et al. (2021). Sequencing of 640,000 exomes identifies GPR75 variants associated with protection from obesity. Science *373*, eabf8683. https://doi.org/10.1126/science.abf8683.
10. Singh, A.S., Mulder, C., Twisk, J.W.R., van Mechelen, W., and Chinapaw, M.J.M. (2008). Tracking of childhood overweight into adulthood: a

systematic review of the literature. Obes. Rev. *9*, 474–488. https://doi.org/10.1111/j.1467-789X.2008.00475.x.

11. Helgeland, Ø., Vaudel, M., Sole-Navais, P., Flatley, C., Juodakis, J., Bacelis, J., Koløen, I.L., Knudsen, G.P., Johansson, B.B., Magnus, P., et al. (2022). Characterization of the genetic architecture of infant and early childhood body mass index. Nat. Metab. *4*, 344–358. https://doi.org/10.1038/s42255-022-00549-1.
12. Bradfield, J.P., Vogelezang, S., Felix, J.F., Chesi, A., Helgeland, Ø., Horikoshi, M., Karhunen, V., Lowry, E., Cousminer, D.L., Ahluwalia, T.S., et al. (2019). A trans-ancestral meta-analysis of genome-wide association studies reveals loci associated with childhood obesity. Hum. Mol. Genet. *28*, 3327–3338. https://doi.org/10.1093/hmg/ddz161.
13. Farooqi, I.S., Keogh, J.M., Yeo, G.S.H., Lank, E.J., Cheetham, T., and O'Rahilly, S. (2003). Clinical spectrum of obesity and mutations in the melanocortin 4 receptor gene. N. Engl. J. Med. *348*, 1085–1095. https://doi.org/10.1056/NEJMoa022050.
14. Krude, H., Biebermann, H., Luck, W., Horn, R., Brabant, G., and Grüters, A. (1998). Severe early-onset obesity, adrenal insufficiency and red hair pigmentation caused by POMC mutations in humans. Nat. Genet. *19*, 155–157. https://doi.org/10.1038/509.
15. Montague, C.T., Farooqi, I.S., Whitehead, J.P., Soos, M.A., Rau, H., Wareham, N.J., Sewter, C.P., Digby, J.E., Mohammed, S.N., Hurst, J.A., et al. (1997). Congenital leptin deficiency is associated with severe early-onset obesity in humans. Nature *387*, 903–908. https://doi.org/10.1038/43185.
16. Day, F.R., Thompson, D.J., Helgason, H., Chasman, D.I., Finucane, H., Sulem, P., Ruth, K.S., Whalen, S., Sarkar, A.K., Albrecht, E., et al. (2017). Genomic analyses identify hundreds of variants associated with age at menarche and support a role for puberty timing in cancer risk. Nat. Genet. *49*, 834–841. https://doi.org/10.1038/ng.3841.
17. Lunetta, K.L., Day, F.R., Sulem, P., Ruth, K.S., Tung, J.Y., Hinds, D.A., Esko, T., Elks, C.E., Altmaier, E., He, C., et al. (2015). Rare coding variants and X-linked loci associated with age at menarche. Nat. Commun. *6*, 7756. https://doi.org/10.1038/ncomms8756.
18. Perry, J.R., Day, F., Elks, C.E., Sulem, P., Thompson, D.J., Ferreira, T., He, C., Chasman, D.I., Esko, T., Thorleifsson, G., et al. (2014). Parent-of-origin-specific allelic associations among 106 genomic loci for age at menarche. Nature *514*, 92–97. https://doi.org/10.1038/nature13545.
19. Lam, B.Y.H., Williamson, A., Finer, S., Day, F.R., Tadross, J.A., Gonçalves Soares, A., Wade, K., Sweeney, P., Bedenbaugh, M.N., Porter, D.T., et al. (2021). MC3R links nutritional state to childhood growth and the timing of puberty. Nature *599*, 436–441. https://doi.org/10.1038/s41586-021-04088-9.
20. Richardson, T.G., Sanderson, E., Elsworth, B., Tilling, K., and Davey Smith, G. (2020). Use of genetic variation to separate the effects of early and later life adiposity on disease risk: mendelian randomisation study. BMJ *369*, m1203. https://doi.org/10.1136/bmj.m1203.
21. Randall, J.C., Winkler, T.W., Kutalik, Z., Berndt, S.I., Jackson, A.U., Monda, K.L., Kilpeläinen, T.O., Esko, T., Mägi, R., Li, S., et al. (2013). Sex-stratified genome-wide association studies including 270,000 individuals show sexual dimorphism in genetic loci for anthropometric traits. PLoS Genet. *9*, e1003500. https://doi.org/10.1371/journal.pgen.1003500.
22. Ruth, K.S., Day, F.R., Tyrrell, J., Thompson, D.J., Wood, A.R., Mahajan, A., Beaumont, R.N., Wittemans, L., Martin, S., Busch, A.S., et al. (2020). Using human genetics to understand the disease impacts of testosterone in men and women. Nat. Med. *26*, 252–258. https://doi.org/10.1038/s41591-020-0751-5.
23. Rentzsch, P., Witten, D., Cooper, G.M., Shendure, J., and Kircher, M. (2019). CADD: predicting the deleteriousness of variants throughout the human genome. Nucleic Acids Res. *47*, D886–D894. https://doi.org/10.1093/nar/gky1016.
24. Nasser, J., Bergman, D.T., Fulco, C.P., Guckelberger, P., Doughty, B.R., Patwardhan, T.A., Jones, T.R., Nguyen, T.H., Ulirsch, J.C., Lekschas, F., et al. (2021). Genome-wide enhancer maps link risk variants to disease genes. Nature *593*, 238–243. https://doi.org/10.1038/s41586-021-03446-x.
25. Felix, J.F., Bradfield, J.P., Monnereau, C., van der Valk, R.J.P., Stergiakouli, E., Chesi, A., Gaillard, R., Feenstra, B., Thiering, E., Kreiner-Møller, E., et al. (2016). Genome-wide association analysis identifies three new susceptibility loci for childhood body mass index. Hum. Mol. Genet. *25*, 389–403. https://doi.org/10.1093/hmg/ddv472.
26. Lotta, L.A., Mokrosiński, J., Mendes de Oliveira, E., Li, C., Sharp, S.J., Luan, J., Brouwers, B., Ayinampudi, V., Bowker, N., Kerrison, N., et al. (2019). Human Gain-of-Function MC4R Variants Show Signaling Bias and Protect against Obesity. Cell *177*, 597–607.e9. https://doi.org/10.1016/j.cell.2019.03.044.
27. Shah, B.P., Sleiman, P.M., Mc Donald, J., Moeller, I.H., and Kleyn, P. (2023). Functional characterization of all missense variants in LEPR, PCSK1, and POMC genes arising from single-nucleotide variants. Expet Rev. Endocrinol. Metabol. *18*, 209–219. https://doi.org/10.1080/17446651.2023.2179985.
28. Deaton, A.M., Dubey, A., Ward, L.D., Dornbos, P., Flannick, J., AMP-T2D-GENES Consortium; Yee, E., Ticau, S., Noetzli, L., Parker, M.M., et al. (2022). Rare loss of function variants in the hepatokine gene INHBE protect from abdominal obesity. Nat. Commun. *13*, 4319. https://doi.org/10.1038/s41467-022-31757-8.
29. Melé, M., Ferreira, P.G., Reverter, F., DeLuca, D.S., Monlong, J., Sammeth, M., Young, T.R., Goldmann, J.M., Pervouchine, D.D., Sullivan, T.J., et al. (2015). Human genomics. The human transcriptome across tissues and individuals. Science *348*, 660–665. https://doi.org/10.1126/science.aaa0355.
30. Young, P., Ehler, E., and Gautel, M. (2001). Obscurin, a giant sarcomeric Rho guanine nucleotide exchange factor protein involved in sarcomere assembly. J. Cell Biol. *154*, 123–136. https://doi.org/10.1083/jcb.200102110.
31. Cabrera-Serrano, M., Caccavelli, L., Savarese, M., Vihola, A., Jokela, M., Johari, M., Capiod, T., Madrange, M., Bugiardini, E., Brady, S., et al. (2022). Bi-allelic loss-of-function OBSCN variants predispose individuals to severe recurrent rhabdomyolysis. Brain *145*, 3985–3998. https://doi.org/10.1093/brain/awab484.
32. Vogelezang, S., Bradfield, J.P., Ahluwalia, T.S., Curtin, J.A., Lakka, T.A., Grarup, N., Scholz, M., van der Most, P.J., Monnereau, C., Stergiakouli, E., et al. (2020). Novel loci for childhood body mass index and shared heritability with adult cardiometabolic traits. PLoS Genet. *16*, e1008718. https://doi.org/10.1371/journal.pgen.1008718.
33. Theilade, S., Christensen, M.B., Vilsbøll, T., and Knop, F.K. (2021). An overview of obesity mechanisms in humans: Endocrine regulation of food intake, eating behaviour and common determinants of body weight. Diabetes Obes. Metabol. *23*, 17–35. https://doi.org/10.1111/dom.14270.
34. Dupuis, J., Langenberg, C., Prokopenko, I., Saxena, R., Soranzo, N., Jackson, A.U., Wheeler, E., Glazer, N.L., Bouatia-Naji, N., Gloyn, A.L., et al. (2010). New genetic loci implicated in fasting glucose homeostasis and their impact on type 2 diabetes risk. Nat. Genet. *42*, 105–116. https://doi.org/10.1038/ng.520.
35. GTEx Consortium (2013). The Genotype-Tissue Expression (GTEx) project. Nat. Genet. *45*, 580–585. https://doi.org/10.1038/ng.2653.
36. Jayarama, S., Li, L.C., Ganesh, L., Mardi, D., Kanteti, P., Hay, N., Li, P., and Prabhakar, B.S. (2014). MADD is a downstream target of PTEN in triggering apoptosis. J. Cell. Biochem. *115*, 261–270. https://doi.org/10.1002/jcb.24657.
37. García-Domingo, D., Ramírez, D., González de Buitrago, G., and Martínez-A, C. (2003). Death inducer-obliterator 1 triggers apoptosis after nuclear translocation and caspase upregulation. Mol. Cell Biol. *23*, 3216–3225. https://doi.org/10.1128/MCB.23.9.3216-3225.2003.
38. Chan, C.W., Lee, Y.B., Uney, J., Flynn, A., Tobias, J.H., and Norman, M. (2007). A novel member of the SAF (scaffold attachment factor)-box protein family inhibits gene expression and induces apoptosis. Biochem. J. *407*, 355–362. https://doi.org/10.1042/BJ20070170.

39. Fütterer, A., Talavera-Gutiérrez, A., Pons, T., de Celis, J., Gutiérrez, J., Domínguez Plaza, V., and Martínez-A, C. (2021). Impaired stem cell differentiation and somatic cell reprogramming in DIDO3 mutants with altered RNA processing and increased R-loop levels. Cell Death Dis. *12*, 637. https://doi.org/10.1038/s41419-021-03906-2.

40. Spegg, V., and Altmeyer, M. (2021). Biomolecular condensates at sites of DNA damage: More than just a phase. DNA Repair *106*, 103179. https://doi.org/10.1016/j.dnarep.2021.103179.

41. Yarden, R.I., Pardo-Reoyo, S., Sgagias, M., Cowan, K.H., and Brody, L.C. (2002). BRCA1 regulates the G2/M checkpoint by activating Chk1 kinase upon DNA damage. Nat. Genet. *30*, 285–289. https://doi.org/10.1038/ng837.

42. Hirao, A., Kong, Y.Y., Matsuoka, S., Wakeham, A., Ruland, J., Yoshida, H., Liu, D., Elledge, S.J., and Mak, T.W. (2000). DNA damage-induced activation of p53 by the checkpoint kinase Chk2. Science *287*, 1824–1827. https://doi.org/10.1126/science.287.5459.1824.

43. Dango, S., Mosammaparast, N., Sowa, M.E., Xiong, L.J., Wu, F., Park, K., Rubin, M., Gygi, S., Harper, J.W., and Shi, Y. (2011). DNA unwinding by ASCC3 helicase is coupled to ALKBH3-dependent DNA alkylation repair and cancer cell proliferation. Mol. Cell *44*, 373–384. https://doi.org/10.1016/j.molcel.2011.08.039.

44. Jia, G., Yang, C.G., Yang, S., Jian, X., Yi, C., Zhou, Z., and He, C. (2008). Oxidative demethylation of 3-methylthymine and 3-methyluracil in single-stranded DNA and RNA by mouse and human FTO. FEBS Lett. *582*, 3313–3319. https://doi.org/10.1016/j.febslet.2008.08.019.

45. Esteller, M., Garcia-Foncillas, J., Andion, E., Goodman, S.N., Hidalgo, O.F., Vanaclocha, V., Baylin, S.B., and Herman, J.G. (2000). Inactivation of the DNA-repair gene MGMT and the clinical response of gliomas to alkylating agents. N. Engl. J. Med. *343*, 1350–1354. https://doi.org/10.1056/NEJM200011093431901.

46. El-Andaloussi, N., Valovka, T., Toueille, M., Steinacher, R., Focke, F., Gehrig, P., Covic, M., Hassa, P.O., Schär, P., Hübscher, U., and Hottiger, M.O. (2006). Arginine methylation regulates DNA polymerase beta. Mol. Cell *22*, 51–62. https://doi.org/10.1016/j.molcel.2006.02.013.

47. Parsons, J.L., Tait, P.S., Finch, D., Dianova, I.I., Edelmann, M.J., Khoronenkova, S.V., Kessler, B.M., Sharma, R.A., McKenna, W.G., and Dianov, G.L. (2009). Ubiquitin ligase ARF-BP1/Mule modulates base excision repair. EMBO J. *28*, 3207–3215. https://doi.org/10.1038/emboj.2009.243.

48. Bessho, T. (1999). Nucleotide excision repair 3' endonuclease XPG stimulates the activity of base excision repairenzyme thymine glycol DNA glycosylase. Nucleic Acids Res. *27*, 979–983. https://doi.org/10.1093/nar/27.4.979.

49. Danial, N.N. (2008). BAD: undertaker by night, candyman by day. Oncogene *27* (*Suppl 1*), S53–S70. https://doi.org/10.1038/onc.2009.44.

50. Jiang, L., Luo, M., Liu, D., Chen, B., Zhang, W., Mai, L., Zeng, J., Huang, N., Huang, Y., Mo, X., and Li, W. (2013). BAD overexpression inhibits cell growth and induces apoptosis via mitochondrial-dependent pathway in non-small cell lung cancer. Cancer Cell Int. *13*, 53. https://doi.org/10.1186/1475-2867-13-53.

51. Czabotar, P.E., Lessene, G., Strasser, A., and Adams, J.M. (2014). Control of apoptosis by the BCL-2 protein family: implications for physiology and therapy. Nat. Rev. Mol. Cell Biol. *15*, 49–63. https://doi.org/10.1038/nrm3722.

52. Moela, P., and Motadi, L.R. (2016). RBBP6: a potential biomarker of apoptosis induction in human cervical cancer cell lines. OncoTargets Ther. *9*, 4721–4735. https://doi.org/10.2147/OTT.S100964.

53. Schumacher, B., Garinis, G.A., and Hoeijmakers, J.H.J. (2008). Age to survive: DNA damage and aging. Trends Genet. *24*, 77–85. https://doi.org/10.1016/j.tig.2007.11.004.

54. Szklarczyk, D., Gable, A.L., Nastou, K.C., Lyon, D., Kirsch, R., Pyysalo, S., Doncheva, N.T., Legeay, M., Fang, T., Bork, P., et al. (2021). The STRING database in 2021: customizable protein-protein networks, and functional characterization of user-uploaded gene/measurement sets. Nucleic Acids Res. *49*, D605–D612. https://doi.org/10.1093/nar/gkaa1074.

55. Hirosawa, M., Nagase, T., Ishikawa, K., Kikuno, R., Nomura, N., and Ohara, O. (1999). Characterization of cDNA clones selected by the GeneMark analysis from size-fractionated cDNA libraries from human brain. DNA Res. *6*, 329–336. https://doi.org/10.1093/dnares/6.5.329.

56. Kursan, S., McMillen, T.S., Beesetty, P., Dias-Junior, E., Almutairi, M.M., Sajib, A.A., Kozak, J.A., Aguilar-Bryan, L., and Di Fulvio, M. (2017). The neuronal K(+)Cl(-) co-transporter 2 (Slc12a5) modulates insulin secretion. Sci. Rep. *7*, 1732. https://doi.org/10.1038/s41598-017-01814-0.

57. Dickinson, M.E., Flenniken, A.M., Ji, X., Teboul, L., Wong, M.D., White, J.K., Meehan, T.F., Weninger, W.J., Westerberg, H., Adissu, H., et al. (2016). High-throughput discovery of novel developmental phenotypes. Nature *537*, 508–514. https://doi.org/10.1038/nature19356.

58. Karczewski, K.J., Francioli, L.C., Tiao, G., Cummings, B.B., Alföldi, J., Wang, Q., Collins, R.L., Laricchia, K.M., Ganna, A., Birnbaum, D.P., et al. (2020). The mutational constraint spectrum quantified from variation in 141,456 humans. Nature *581*, 434–443. https://doi.org/10.1038/s41586-020-2308-7.

59. Schievella, A.R., Chen, J.H., Graham, J.R., and Lin, L.L. (1997). MADD, a novel death domain protein that interacts with the type 1 tumor necrosis factor receptor and activates mitogen-activated protein kinase. J. Biol. Chem. *272*, 12069–12075. https://doi.org/10.1074/jbc.272.18.12069.

60. GTEx Consortium (2020). The GTEx Consortium atlas of genetic regulatory effects across human tissues. Science *369*, 1318–1330. https://doi.org/10.1126/science.aaz1776.

61. Anazi, S., Maddirevula, S., Salpietro, V., Asi, Y.T., Alsahli, S., Alhashem, A., Shamseldin, H.E., AlZahrani, F., Patel, N., Ibrahim, N., et al. (2017). Expanding the genetic heterogeneity of intellectual disability. Hum. Genet. *136*, 1419–1429. https://doi.org/10.1007/s00439-017-1843-2.

62. Schneeberger, P.E., Kortüm, F., Korenke, G.C., Alawi, M., Santer, R., Woidy, M., Buhas, D., Fox, S., Juusola, J., Alfadhel, M., et al. (2020). Biallelic MADD variants cause a phenotypic spectrum ranging from developmental delay to a multisystem disorder. Brain *143*, 2437–2453. https://doi.org/10.1093/brain/awaa204.

63. Thearle, M.S., Muller, Y.L., Hanson, R.L., Mullins, M., Abdussamad, M., Tran, J., Knowler, W.C., Bogardus, C., Krakoff, J., and Baier, L.J. (2012). Greater impact of melanocortin-4 receptor deficiency on rates of growth and risk of type 2 diabetes during childhood compared with adulthood in Pima Indians. Diabetes *61*, 250–257. https://doi.org/10.2337/db11-0708.

64. Abel, T.W., and Rance, N.E. (1999). Proopiomelanocortin gene expression is decreased in the infundibular nucleus of postmenopausal women. Brain Res. Mol. Brain Res. *69*, 202–208. https://doi.org/10.1016/s0169-328x(99)00111-4.

65. Lloyd, J.M., Scarbrough, K., Weiland, N.G., and Wise, P.M. (1991). Age-related changes in proopiomelanocortin (POMC) gene expression in the periarcuate region of ovariectomized rats. Endocrinology *129*, 1896–1902. https://doi.org/10.1210/endo-129-4-1896.

66. Włodarczyk, M., Jabłonowska-Lietz, B., Olejarz, W., and Nowicka, G. (2018). Anthropometric and Dietary Factors as Predictors of DNA Damage in Obese Women. Nutrients *10*, 578. https://doi.org/10.3390/nu10050578.

67. Donmez-Altuntas, H., Sahin, F., Bayram, F., Bitgen, N., Mert, M., Guclu, K., Hamurcu, Z., Arıbas, S., Gundogan, K., and Diri, H. (2014). Evaluation of chromosomal damage, cytostasis, cytotoxicity, oxidative DNA damage and their association with body-mass index in obese subjects. Mutat. Res., Genet. Toxicol. Environ. Mutagen. *771*, 30–36. https://doi.org/10.1016/j.mrgentox.2014.06.006.

68. Jang, Y., Kim, O.Y., Ryu, H.J., Kim, J.Y., Song, S.H., Ordovas, J.M., and Lee, J.H. (2003). Visceral fat accumulation determines postprandial lipemic response, lipid peroxidation, DNA damage, and endothelial dysfunction in nonobese Korean men. J. Lipid Res. *44*, 2356–2364. https://doi.org/10.1194/jlr.M300233-JLR200.

69. Fieres, J., Fischer, M., Sauter, C., Moreno-Villanueva, M., Bürkle, A., and Wirtz, P.H. (2022). The burden of overweight: Higher body mass index, but not vital exhaustion, is associated with higher DNA damage and lower DNA repair capacity. DNA Repair *114*, 103323. https://doi.org/10.1016/j.dnarep.2022.103323.

70. McCullough, L.E., Eng, S.M., Bradshaw, P.T., Cleveland, R.J., Steck, S.E., Terry, M.B., Shen, J., Crew, K.D., Rossner, P., Jr., Ahn, J., et al. (2015). Genetic polymorphisms in DNA repair and oxidative stress pathways may modify the association between body size and postmenopausal breast cancer. Ann. Epidemiol. *25*, 263–269. https://doi.org/10.1016/j.annepidem.2015.01.009.

71. Rupérez, A.I., Gil, A., and Aguilera, C.M. (2014). Genetics of oxidative stress in obesity. Int. J. Mol. Sci. *15*, 3118–3144. https://doi.org/10.3390/ijms15023118.

72. Vergoni, B., Cornejo, P.J., Gilleron, J., Djedaini, M., Ceppo, F., Jacquel, A., Bouget, G., Ginet, C., Gonzalez, T., Maillet, J., et al. (2016). DNA Damage and the Activation of the p53 Pathway Mediate Alterations in Metabolic and Secretory Functions of Adipocytes. Diabetes *65*, 3062–3074. https://doi.org/10.2337/db16-0014.

73. Lee, G., Kim, Y.Y., Jang, H., Han, J.S., Nahmgoong, H., Park, Y.J., Han, S.M., Cho, C., Lim, S., Noh, J.R., et al. (2022). SREBP1c-PARP1 axis tunes anti-senescence activity of adipocytes and ameliorates metabolic imbalance in obesity. Cell Metab. *34*, 702–718.e5. https://doi.org/10.1016/j.cmet.2022.03.010.

74. Pierce, A.A., and Xu, A.W. (2010). De novo neurogenesis in adult hypothalamus as a compensatory mechanism to regulate energy balance. J. Neurosci. *30*, 723–730. https://doi.org/10.1523/JNEUROSCI.2479-09.2010.

75. Djogo, T., Robins, S.C., Schneider, S., Kryzskaya, D., Liu, X., Mingay, A., Gillon, C.J., Kim, J.H., Storch, K.F., Boehm, U., et al. (2016). Adult NG2-Glia Are Required for Median Eminence-Mediated Leptin Sensing and Body Weight Control. Cell Metab. *23*, 797–810. https://doi.org/10.1016/j.cmet.2016.04.013.

76. McNay, D.E.G., Briançon, N., Kokoeva, M.V., Maratos-Flier, E., and Flier, J.S. (2012). Remodeling of the arcuate nucleus energy-balance circuit is inhibited in obese mice. J. Clin. Invest. *122*, 142–152. https://doi.org/10.1172/JCI43134.

77. Li, P., Jayarama, S., Ganesh, L., Mordi, D., Carr, R., Kanteti, P., Hay, N., and Prabhakar, B.S. (2010). Akt-phosphorylated mitogen-activated kinase-activating death domain protein (MADD) inhibits TRAIL-induced apoptosis by blocking Fas-associated death domain (FADD) association with death receptor 4. J. Biol. Chem. *285*, 22713–22722. https://doi.org/10.1074/jbc.M110.105692.

78. Braig, S., and Bosserhoff, A.K. (2013). Death inducer-obliterator 1 (Dido1) is a BMP target gene and promotes BMP-induced melanoma progression. Oncogene *32*, 837–848. https://doi.org/10.1038/onc.2012.115.

79. Liu, Y., Kim, H., Liang, J., Lu, W., Ouyang, B., Liu, D., and Songyang, Z. (2014). The death-inducer obliterator 1 (Dido1) gene regulates embryonic stem cell self-renewal. J. Biol. Chem. *289*, 4778–4786. https://doi.org/10.1074/jbc.M113.486290.

80. Guerrero, A.A., Gamero, M.C., Trachana, V., Fütterer, A., Pacios-Bras, C., Díaz-Concha, N.P., Cigudosa, J.C., Martínez-A, C., and van Wely, K.H.M. (2010). Centromere-localized breaks indicate the generation of DNA damage by the mitotic spindle. Proc. Natl. Acad. Sci. USA *107*, 4159–4164. https://doi.org/10.1073/pnas.0912143106.

81. Trachana, V., van Wely, K.H.M., Guerrero, A.A., Fütterer, A., and Martínez-A, C. (2007). Dido disruption leads to centrosome amplification and mitotic checkpoint defects compromising chromosome stability. Proc. Natl. Acad. Sci. USA *104*, 2691–2696. https://doi.org/10.1073/pnas.0611132104.

82. Altmeyer, M., Toledo, L., Gudjonsson, T., Grøfte, M., Rask, M.B., Lukas, C., Akimov, V., Blagoev, B., Bartek, J., and Lukas, J. (2013). The chromatin scaffold protein SAFB1 renders chromatin permissive for DNA damage signaling. Mol. Cell *52*, 206–220. https://doi.org/10.1016/j.molcel.2013.08.025.

83. Villares, R., Gutiérrez, J., Fütterer, A., Trachana, V., Gutiérrez del Burgo, F., and Martínez-A, C. (2015). Dido mutations trigger perinatal death and generate brain abnormalities and behavioral alterations in surviving adult mice. Proc. Natl. Acad. Sci. USA *112*, 4803–4808. https://doi.org/10.1073/pnas.1419300112.

84. van der Klaauw, A.A., Croizier, S., Mendes de Oliveira, E., Stadler, L.K.J., Park, S., Kong, Y., Banton, M.C., Tandon, P., Hendricks, A.E., Keogh, J.M., et al. (2019). Human Semaphorin 3 Variants Link Melanocortin Circuit Development and Energy Balance. Cell *176*, 729–742.e18. https://doi.org/10.1016/j.cell.2018.12.009.

85. Yang, D.J., Hong, J., and Kim, K.W. (2021). Hypothalamic primary cilium: A hub for metabolic homeostasis. Exp. Mol. Med. *53*, 1109–1115. https://doi.org/10.1038/s12276-021-00644-5.

86. Kane, M.S., Diamonstein, C.J., Hauser, N., Deeken, J.F., Niederhuber, J.E., and Vilboux, T. (2019). Endosomal trafficking defects in patient cells with KIAA1109 biallelic variants. Genes Dis. *6*, 56–67. https://doi.org/10.1016/j.gendis.2018.12.004.

87. Bycroft, C., Freeman, C., Petkova, D., Band, G., Elliott, L.T., Sharp, K., Motyer, A., Vukcevic, D., Delaneau, O., O'Connell, J., et al. (2018). The UK Biobank resource with deep phenotyping and genomic data. Nature *562*, 203–209. https://doi.org/10.1038/s41586-018-0579-z.

88. Bulik-Sullivan, B.K., Loh, P.R., Finucane, H.K., Ripke, S., Yang, J., Schizophrenia Working Group of the Psychiatric Genomics, C., Patterson, N., Daly, M.J., Price, A.L., and Neale, B.M. (2015). LD Score regression distinguishes confounding from polygenicity in genome-wide association studies. Nat. Genet. *47*, 291–295. https://doi.org/10.1038/ng.3211.

89. Gardner, E.J., Kentistou, K.A., Stankovic, S., Lockhart, S., Wheeler, E., Day, F.R., Kerrison, N.D., Wareham, N.J., Langenberg, C., O'Rahilly, S., et al. (2022). Damaging missense variants in IGF1R implicate a role for IGF-1 resistance in the etiology of type 2 diabetes. Cell Genom. *2*, 100208. https://doi.org/10.1016/j.xgen.2022.100208.

90. Loh, P.R., Tucker, G., Bulik-Sullivan, B.K., Vilhjálmsson, B.J., Finucane, H.K., Salem, R.M., Chasman, D.I., Ridker, P.M., Neale, B.M., Berger, B., et al. (2015). Efficient Bayesian mixed-model analysis increases association power in large cohorts. Nat. Genet. *47*, 284–290. https://doi.org/10.1038/ng.3190.

91. Clogg, C.C., Petkova, E., and Haritou, A. (1995). Statistical Methods for Comparing Regression Coefficients Between Models. Am. J. Sociol. *100*, 1261–1293. https://doi.org/10.1086/230638.

92. Li, X., Li, Z., Zhou, H., Gaynor, S.M., Liu, Y., Chen, H., Sun, R., Dey, R., Arnett, D.K., Aslibekyan, S., et al. (2020). Dynamic incorporation of multiple in silico functional annotations empowers rare variant association analysis of large whole-genome sequencing studies at scale. Nat. Genet. *52*, 969–983. https://doi.org/10.1038/s41588-020-0676-4.

93. UniProt Consortium (2023). UniProt: the Universal Protein Knowledgebase in 2023. Nucleic Acids Res. *51*, D523–D531. https://doi.org/10.1093/nar/gkac1052.

94. Pándy-Szekeres, G., Munk, C., Tsonkov, T.M., Mordalski, S., Harpsøe, K., Hauser, A.S., Bojarski, A.J., and Gloriam, D.E. (2018). GPCRdb in 2018: adding GPCR structure models and ligands. Nucleic Acids Res. *46*, D440–D446. https://doi.org/10.1093/nar/gkx1109.

95. Stankovic, S., Shekari, S., Huang, Q.Q., Gardner, E.J., Owens, N.D.L., Azad, A., Hawkes, G., Kentistou, K.A., Beaumont, R.N., Day, F.R., et al. (2022). Genetic susceptibility to earlier ovarian ageing increases de novo mutation rate in offspring. medRxiv. https://doi.org/10.1101/2022.06.23.22276698.

96. Yang, J., Lee, S.H., Goddard, M.E., and Visscher, P.M. (2011). GCTA: a tool for genome-wide complex trait analysis. Am. J. Hum. Genet. *88*, 76–82. https://doi.org/10.1016/j.ajhg.2010.11.011.

97. Purcell, S., Neale, B., Todd-Brown, K., Thomas, L., Ferreira, M.A.R., Bender, D., Maller, J., Sklar, P., de Bakker, P.I.W., Daly, M.J., and

Sham, P.C. (2007). PLINK: a tool set for whole-genome association and population-based linkage analyses. Am. J. Hum. Genet. *81*, 559–575. https://doi.org/10.1086/519795.

98. Boughton, A.P., Welch, R.P., Flickinger, M., VandeHaar, P., Taliun, D., Abecasis, G.R., and Boehnke, M. (2021). LocusZoom.js: Interactive and embeddable visualization of genetic association study results. Bioinformatics *37*, 3017–3018. https://doi.org/10.1093/bioinformatics/btab186.

99. Mahajan, A., Taliun, D., Thurner, M., Robertson, N.R., Torres, J.M., Rayner, N.W., Payne, A.J., Steinthorsdottir, V., Scott, R.A., Grarup, N., et al. (2018). Fine-mapping type 2 diabetes loci to single-variant resolution using high-density imputation and islet-specific epigenome maps. Nat. Genet. *50*, 1505–1513. https://doi.org/10.1038/s41588-018-0241-6.

100. Giambartolomei, C., Vukcevic, D., Schadt, E.E., Franke, L., Hingorani, A.D., Wallace, C., and Plagnol, V. (2014). Bayesian test for colocalisation between pairs of genetic association studies using summary statistics. PLoS Genet. *10*, e1004383. https://doi.org/10.1371/journal.pgen.1004383.

101. de Leeuw, C.A., Mooij, J.M., Heskes, T., and Posthuma, D. (2015). MAGMA: generalized gene-set analysis of GWAS data. PLoS Comput. Biol. *11*, e1004219. https://doi.org/10.1371/journal.pcbi.1004219.

102. Locke, A.E., Kahali, B., Berndt, S.I., Justice, A.E., Pers, T.H., Day, F.R., Powell, C., Vedantam, S., Buchkovich, M.L., Yang, J., et al. (2015). Genetic studies of body mass index yield new insights for obesity biology. Nature *518*, 197–206. https://doi.org/10.1038/nature14177.

103. Aksglaede, L., Juul, A., Olsen, L.W., and Sørensen, T.I.A. (2009). Age at puberty and the emerging obesity epidemic. PLoS One *4*, e8450. https://doi.org/10.1371/journal.pone.0008450.

104. Kamat, M.A., Blackshaw, J.A., Young, R., Surendran, P., Burgess, S., Danesh, J., Butterworth, A.S., and Staley, J.R. (2019). PhenoScanner V2: an expanded tool for searching human genotype-phenotype associations. Bioinformatics *35*, 4851–4853. https://doi.org/10.1093/bioinformatics/btz469.

105. Staley, J.R., Blackshaw, J., Kamat, M.A., Ellis, S., Surendran, P., Sun, B.B., Paul, D.S., Freitag, D., Burgess, S., Danesh, J., et al. (2016). PhenoScanner: a database of human genotype-phenotype associations. Bioinformatics *32*, 3207–3209. https://doi.org/10.1093/bioinformatics/btw373.

106. Ghoussaini, M., Mountjoy, E., Carmona, M., Peat, G., Schmidt, E.M., Hercules, A., Fumis, L., Miranda, A., Carvalho-Silva, D., Buniello, A., et al. (2021). Open Targets Genetics: systematic identification of trait-associated genes using large-scale genetics and functional genomics. Nucleic Acids Res. *49*, D1311–D1320. https://doi.org/10.1093/nar/gkaa840.

107. Mountjoy, E., Schmidt, E.M., Carmona, M., Schwartzentruber, J., Peat, G., Miranda, A., Fumis, L., Hayhurst, J., Buniello, A., Karim, M.A., et al. (2021). An open approach to systematically prioritize causal variants and genes at all published human GWAS trait-associated loci. Nat. Genet. *53*, 1527–1533. https://doi.org/10.1038/s41588-021-00945-5.

STAR★METHODS

KEY RESOURCES TABLE

REAGENT or RESOURCE	SOURCE	IDENTIFIER
Deposited data		
UK Biobank Data returns (to be submitted on publication)	UK Biobank	Application: 9905
UK Biobank phenotypic data	UK Biobank	Application: 9905
UK Biobank whole exome sequence data (450,000 release)	UK Biobank	Data field: 23148
Open Targets Genetics Platform	https://genetics.opentargets.org/	N/A
PhenoScanner	http://www.phenoscanner.medschl.cam.ac.uk/	N/A
UniProt	https://www.uniprot.org/	N/A
GPCRdb	https://gpcrdb.org/	N/A
IMPC (detailed in Table S19)	https://www.mousephenotype.org/	Accessed November 2022
Software and algorithms		
BOLT-LMM	https://alkesgroup.broadinstitute.org/BOLT-LMM/BOLT-LMM_manual.html	v2.3.6
STAAR	https://github.com/xihaoli/STAAR	v0.9.6
bcftools	https://github.com/samtools/bcftools	v1.14
MRC-Epid WES pipeline	https://github.com/mrcepid-rap/	N/A
plink	https://www.cog-genomics.org/plink/	v1.90b6.18
Variant Effect Predictor (VEP)	https://www.ensembl.org/info/docs/tools/vep/index.html	v104
LDSC	https://github.com/bulik/ldsc	v1.0.1
R	https://www.r-project.org/	v4.2.1
coloc R package	https://cran.r-project.org/web/packages/coloc/index.html	v5.1.0
sandwich R package	https://cran.r-project.org/web/packages/sandwich/index.html	v3.0-2
ordinal R package	https://cran.r-project.org/web/packages/ordinal/index.html	v2019.12–10
ggplot2 R package	https://cran.r-project.org/web/packages/ggplot2/index.html	v3.3.6
MAGMA	https://ctg.cncr.nl/software/magma	v1.09
GCTA	https://yanglab.westlake.edu.cn/software/gcta/#Overview	N/A
Locus zoom	https://locuszoom.org/	v1.4
plink	https://zzz.bwh.harvard.edu/plink/	v1.90.b6.18
STRING	https://string-db.org/	v11.5

RESOURCE AVAILABILITY

Lead contact
Further information and requests for resources should be directed to and will be fulfilled by the lead contact, John R.B. Perry (john.perry@mrc-epid.cam.ac.uk).

Materials availability
No materials were generated in this study.

Data and code availability

Rare variant burden testing summary statistics are included in the supplemental information of this paper. Protected UK Biobank participant data will be returned to the UK Biobank resource and be accessible via application number 9905. This paper does not report original code. Any additional information required to reanalyse the data reported in this paper is available from the lead contact upon request.

METHOD DETAILS

Exome-wide gene burden associations with BMI and SAC10

To identify genes associated with sex-stratified adult adiposity, we performed an ExWAS using WES data derived from 419,692 European genetic-ancestry UK Biobank participants (191,864 males and 227,828 females).[8] As our outcome, we used adult BMI (kg/m^2) from field 21001. Sex in our study was defined using the 'genetic sex' parameter by Bycroft et al.,[87] and provided on UK Biobank field 22001. To identify genes associated with sex-combined childhood adiposity, we performed an ExWAS using WES data derived from 414,032 European genetic-ancestry UK Biobank participants (188,777 males and 225,255 females).[8] As our outcome, we used SAC10 from field 1687, which is based on the question, "When you were 10 years old, compared to average would you describe yourself as thinner, plumper or about average?" and treated it as a continuous variable (0 = thinner, 1 = average, 2 = plumper). Although this phenotype is a proxy measure of childhood adiposity based on recalled data, it shows a strong genetic correlation with childhood BMI (r_g = 0.94)[25] and only a moderate correlation with adult BMI (r_g = 0.55) as calculated with LDSC.[88]

Data processing and quality control were performed as described in Gardner et al.[89] Individual gene burden tests were performed by collapsing exome variants according to their predicted functional consequence. We defined two functional categories of exome variants with a MAF<0.1% 1) high-confidence protein truncating variants (HC_PTV) and 2) damaging variants (DMG) which contain both high-confidence PTVs and missense variants as defined by a CADD score threshold of $\geq$25[23]. We defined Protein Truncating Variants (PTVs) as Variant Effect Predictor consequence of stop gained, frameshift, or splice acceptor/donor. To define 'high-confidence', we used the LOFTEE algorithm.[58] We analyzed a maximum of 18,107 protein-coding genes with a minimum of >10 rare allele carriers in any of the tested categories. The burden association tests were conducted using BOLT-LMM.[90] Our results are statistically well-calibrated as indicated by the absence of significant associations with synonymous variant burden (Figure S1, Table S1).

Sexual dimorphism was ascertained by comparing the association effect sizes between the male- and female-only analyses, as outlined below (where f denotes the female association summary statistics and m denotes the male ones)[91]:

$$z = \frac{\beta_f - \beta_m}{\sqrt{se_f^2 + se_m^2}}$$

Associations were deemed dimorphic if their Bonferroni-corrected P value for the above z-statistic was <0.05 and the association did not reach nominal significance ($\geq$0.05) in the opposite sex.

Carriers of genes highlighted in ExWAS were classified as homozygous carriers if they carried two copies of the same mutation and compound heterozygous if they carried two mutations in the same gene >5 basepairs apart (Table S11).

For all exome-wide significantly associated genes, the following further models were conducted.

Sensitivity analyses

Several sensitivity analyses were conducted to corroborate the identified associations. To validate our BOLT-LMM results, we additionally conducted burden association tests using STAAR[92] as described in Gardner et al.,[89] testing the same protein-coding transcripts as in our primary analyses. We also used an inverse-rank normalised BMI variable in the above-described BOLT-LMM framework to reduce the positive skew. We validated our associations by using linear models in R in the White-European unrelated subsample of UK Biobank for the equivalent discovery phenotypes and for T2D. To these models, we also applied heteroscedasticity-robust standard error calculations using the sandwich R package (3.0–2), to address case-control imbalances (Table S4). Furthermore, to test whether age at recruitment (field 21022) influenced BMI, we calculated the mean BMI of carriers of genes identified in the BMI ExWAS stratified by age ($\geq$58 years and <58 years, with 58 years being the median age at recruitment for all UK Biobank participants). To determine a difference in means, we used the same formula as above[91] and used a P value threshold of 0.05 (Table S14).

Finally, to ascertain whether the gene-level associations with DMG variants in *POMC* and MC4R might be driven by variants in known functional domains, we conducted domain-level burden tests (Table S9). To do this, variants were separated into the different functional domains within *POMC* using information from UniProt,[93] while *MC4R* domains were also annotated using GPCRdb.[94] Domain-level burden tests with sex-combined SAC10 were then performed using linear models, for domains that included at least 2 variants. We also tested known functionally implicated variants within these two genes (Table S10). To do this we used functionally validated loss- or gain-of function variants in *MC4R* reported by Lotta et al.,[26] where 31/61 described variants were found in UK Biobank and in *POMC* by Shah et al.,[27] where 15/1576 variants were found in UK Biobank.

Exome lookup in related metabolic traits

The exome-wide significantly associated genes were further tested for associations toward T2D risk, SHBG and free testosterone levels and WHRadjBMI within UK Biobank using BOLT-LMM, as described above (Table S5). For WHRadjBMI, waist-hip ratio was calculated using fields 48 and 49 and BMI from field 21001 from the first available instance where they were all available. For T2D, the phenotype was derived as described in Gardner et al..[89] Using this trait, we performed logistic regressions in the unrelated white European subsample of UK Biobank to derive odds ratios (in R, v4.2.1). For SHBG, hormone levels were extracted from the first instance data of field 30830 and log-transformed, after removing participants taking hormone-influencing medications, including current reported use of HRT or oral contraception. For free testosterone, testosterone levels were extracted from the first instance data of field 30850 and the Vermeulen method was used in conjunction with data on SHBG, total testosterone levels and albumin (from field 30600) to calculate free testosterone levels. These were then log-transformed, after removing participants taking hormone-influencing medications. Finally, we tested for associations between genes identified in the female-only BMI analysis and a derived binary menopausal status phenotype, as described in Stankovic et al.,[95] using linear models in the white-European unrelated subsample of the discovery cohort (Table S6). Interactions between menopause- and carrier-status for qualifying variants in these genes were also tested for BMI, using R.

Comparison of variance explained in childhood versus adult body size

To understand whether any of the exome-wide significantly associated genes may exert stronger effects in childhood than in adulthood or vice versa, we compared the variance explained across the two traits (BMI and SAC10) by being a carrier of qualifying mutations in any of the identified genes. Using R, BMI and SAC10 were first adjusted for the standard covariates (sex, age, age,[2] exome-sequencing batch and the first 10 principal components) and the residual trait variance was tested against binary carrier status for each gene. The resulting model adjusted R^2 was used as a scaled and comparable indication of the effect magnitude across the two outcomes.

Ordered logit models of obesity outcomesand carrier status of OBSCN

We conducted cumulative link models using childhood and adult obesity as ordered categorical outcomes, to quantify the relative risk of obesity conferred by carrying qualifying variants in any of the exome-wide significantly associated genes. To do this, we used the three levels of comparative size at age ten; “Thinner”, “Average”, “Plumper” and we similarly split adult BMI into three categories; BMI less than 20, BMI between 20 and 30, and BMI over 30. To estimate the effect of carrier status of *OBSCN* PTVs on SAC10, we used four levels; “homozygous”, “compound heterozygous”, “heterozygous”, and “non-carriers” (Table S12). Analyses were conducted using the “clm” function in the “ordinal” R package (v2019.12–10).

All data manipulations were conducted in R (v4.2.1) and plots were generated using ggplot2 (v3.3.6).

Common variant GWAS

GWAS signals proximal to the exome-identified genes

Common variant associations at the exome-identified genes were queried using the equivalent common variant GWAS (MAF>0.1%) in UK Biobank (adult BMI, N = 450,706, or SAC10, N = 444,345). Signal selection was performed as follows: genome-wide significant signals ($p < 5 \times 10^{-8}$) were initially selected based on proximity, in 1Mb windows. Secondary signals within these windows were then identified using the approximate conditional analysis in GCTA,[96] using an LD reference panel derived from 25,000 participants of the UK Biobank study. Only secondary signals that were uncorrelated (R^2<5%) with each other and did not exhibit an overt change in their association statistics between the baseline and conditional models (β changed by less than 20% or p value changed by less than four orders of magnitude) were kept. The lists of primary and secondary signals were further checked for pairwise LD within 10Mb windows, using plink (v1.90b6.18)[97] and only independent signals (R^2<5%) were kept, prioritising the distance-based ones in the case of linkage. The subsequent regions were plotted using LocusZoom (v1.4)[98] and any identified GWAS signals were also queried in a GWAS meta-analysis of T2D.[99]

Signals were then annotated with their closest gene (within 500kb up- or downstream of the signal), using the NCBI RefSeq gene map for GRCh37 (via http://hgdownload.soe.ucsc.edu/goldenPath/hg19/database/). As most GWAS signals are intronic or intergenic, we overlayed these associations with other datasets to understand whether the GWAS variants can be causally linked to changes in the exome-identified genes’ regulation. For genes with proximal GWAS signals, we calculated genomic windows of high linkage disequilibrium (LD; $R^2 > 0.8$) for each given signal using plink and mapped these to the locations of known enhancers for the target genes, using the activity-by-contact (ABC) enhancer maps.[24] Any seen overlaps indicate whether the genomic variants associating with the traits of interest directly changed the sequence of enhancers for the genes in question. We also performed co-localization analyses between the GWAS and eQTL data using the ABF function within the R package “coloc” (v5.1.0)[100] and the cross-tissue meta-analysed GTEx eQTL data (V7, available via https://gtexportal.org and using the fixed-effects summary statistics).[35] For this, variants within a 500kb window of each gene that were common between the GWAS and eQTL data were used and an H4 posterior probability (the probability of a single, shared causal variant) $\geq$0.75 was used as a colocalization threshold. Finally, outwith transcriptional changes, we performed a gene-level Multi-marker Analysis of GenoMic Annotation (MAGMA, v1.09) analysis,[101] to collapse all observed genomic variants within each of the identified genes and calculate aggregate gene-level associations to the phenotypic traits. To do this, we specifically used common (MAF>0.1%) exonic variants within each gene (Table S8).

DDR pathway enrichment analyses

To ascertain the signal enrichment in genes related to DDR processes at the genome-wide level, we used the MAGMA gene-level associations as described above. We then collapsed this gene-level data into three pathways; GO cellular response to DNA damage stimulus (GO:0006974), GO DNA repair (GO:0006281) and an expert-curated broad DDR pathway (Table S20) and tested for enrichment against them under the MAGMA gene-set analysis functionality (Table S15).

Definition of GWAS signal trajectories

'Adult-specific' signals were defined as associated with adult BMI in UKBB with independent confirmation ($p < 0.05$) in GIANT consortium data[102] but not associated ($p>=0.05$) with SAC10 and female pubertal timing (as measured by recalled age at menarche in UK Biobank) (which is sensitive to childhood adiposity[103]) and without a reported stronger association with a related lifestyle (e.g., alcohol consumption) or mental health trait (in PhenoScanner[104,105] or Open Target Genetics[106,107]) (Tables S16 and S21). 'Childhood-specific' signals were defined as being associated with SAC10 in UK Biobank with independent confirmation ($p < 0.05$) in EGG consortium childhood BMI data[25] and female pubertal timing (as measured by recalled age at menarche in UK Biobank) but not associated with adult BMI in UK Biobank ($p>=0.05$) (Table S17). Life-course-acting signals were defined as influencing both adult and childhood adiposity as measured adult BMI and SAC10 ($p < 0.05$). Furthermore, since a large number of BMI and SAC10 signals are expected to be the at the same locus, we only considered SAC10 signals that were independent of any BMI signal ($R^2 < 0.05$) calculated as described above. For signals with missing data in the look-up GWAS, we identified proxies using an LD reference panel derived from 25,000 participants of the UK Biobank study (within 1 megabase of the reported signal and $R^2 > 0.6$), choosing the variant with the highest R^2 value.

We performed a gene-centric pathway analyses based on the closest gene for the 'adult-specific', 'childhood-specific' and 'life-course-acting' SNPs using STRING (https://string-db.org/).[54] We tested for enrichment against all 'Gene Ontology Biological Process (GO:BP)' terms as well as KEGG, REACTOME and WikiPathway pathways. Any term with an adjusted p value <5% (Benjamini-Hochberg method) was considered to be statistically significantly (Table S18).

Cell Genomics, Volume *3*

Supplemental information

Large-scale exome sequence analysis identifies sex- and age-specific determinants of obesity

Lena R. Kaisinger, Katherine A. Kentistou, Stasa Stankovic, Eugene J. Gardner, Felix R. Day, Yajie Zhao, Alexander Mörseburg, Christopher J. Carnie, Guido Zagnoli-Vieira, Fabio Puddu, Stephen P. Jackson, Stephen O'Rahilly, I. Sadaf Farooqi, Laura Dearden, Lucas C. Pantaleão, Susan E. Ozanne, Ken K. Ong, and John R.B. Perry

SUPPLEMENTAL INFORMATION

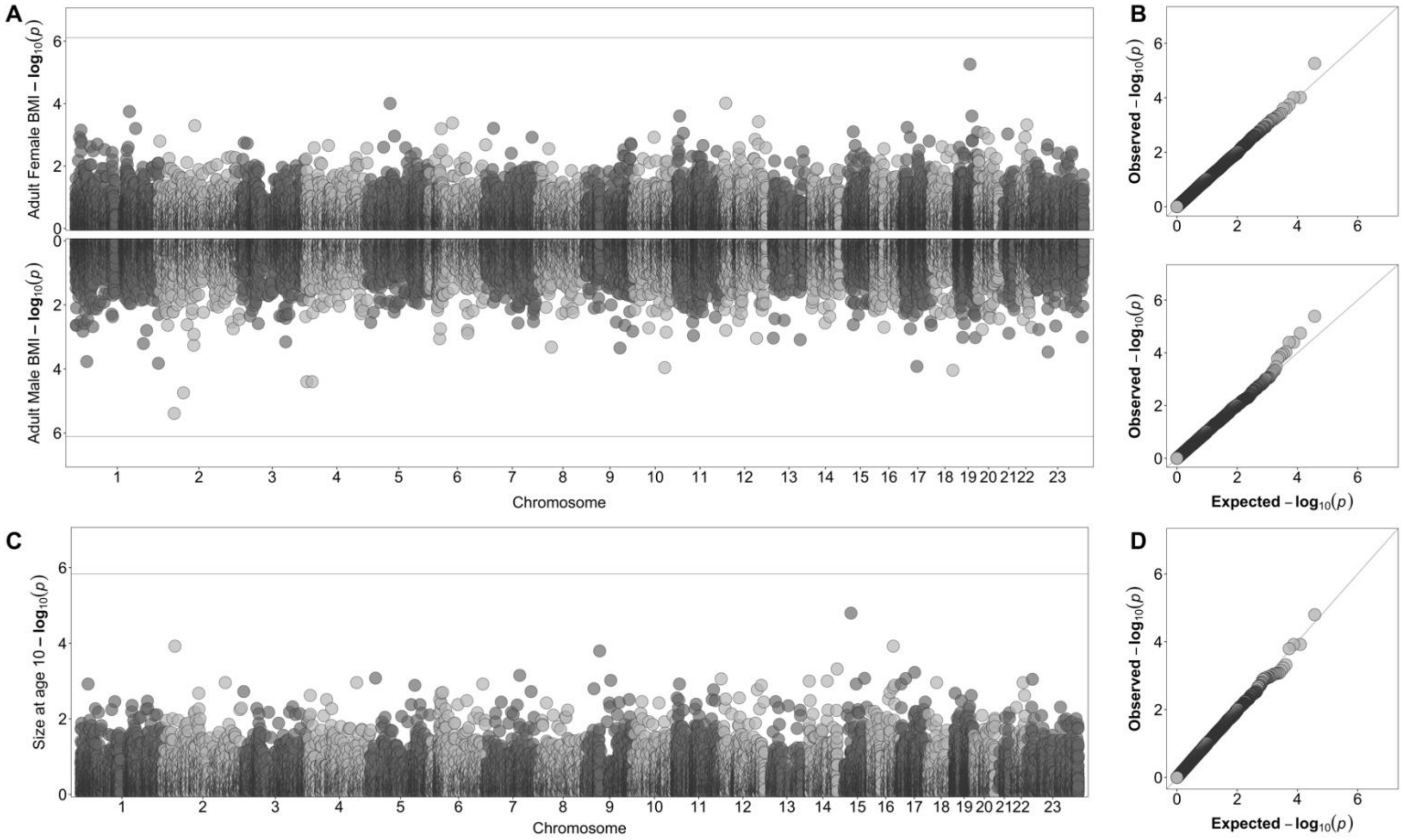

Figure S1 | Gene burden associations for synonymous rare variants with adult BMI and comparative body size at age 10, related to Figure 1 & 4. (A/C) Manhattan plot showing synonymous gene burden results for BMI and SAC10, respectively. The exome-wide significance thresholds used during discovery are indicated by the horizontal lines. (B/D) QQ plot of corresponding data.

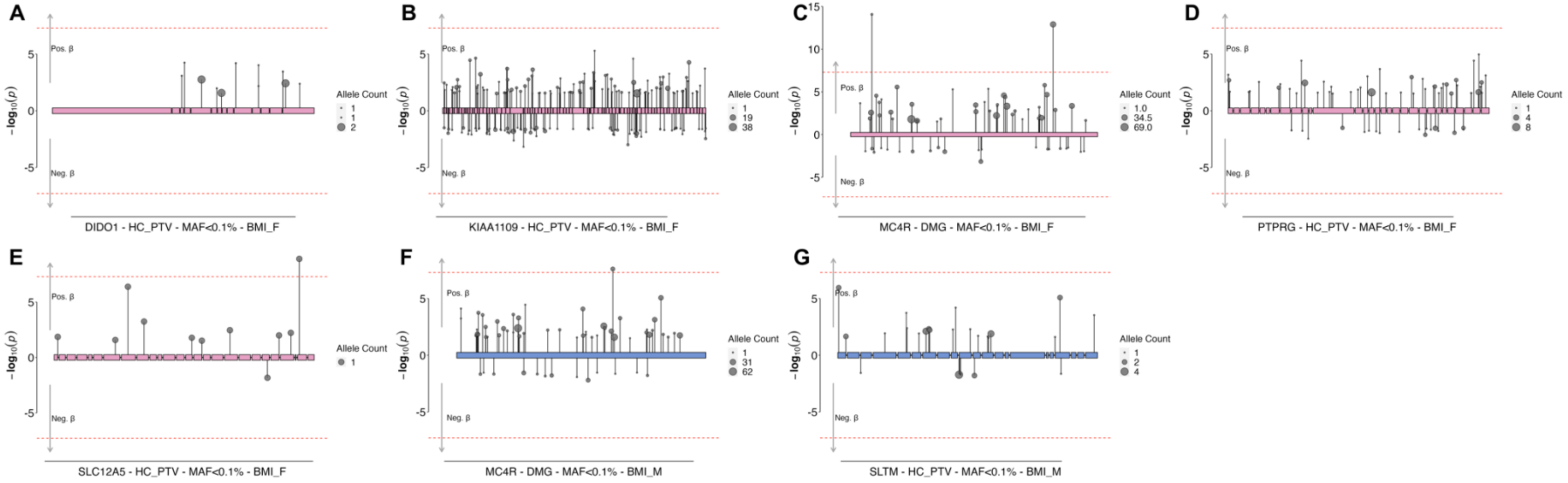

Figure S2 | Exome associations between the identified genes and female or male BMI in UK Biobank, related to Figure 1 & 2. Variant-level associations between variants within *DIDO1* (A), *KIAA1109* (B), *MC4R* (C), *PTPRG* (D), *SLC12A5* (E) and BMI in the women-only analysis and *MC4R* (F), *SLTM* (G) and BMI in the men-only analysis. Included variants from our discovery analyses had a minor allele frequency (MAF) <0.1% and were annotated to either be high-confidence protein truncating variants or missense variants with a high CADD score (≥25). Each variant is presented as an individual line extending to its association p-value (-log10), in the direction indicating the direction of effect on BMI in carriers of the alternate allele, while the point size indicates the number of carriers of each variant (i.e. allele count), as indicated in the figure legend. Extended data can be found in Table S3.

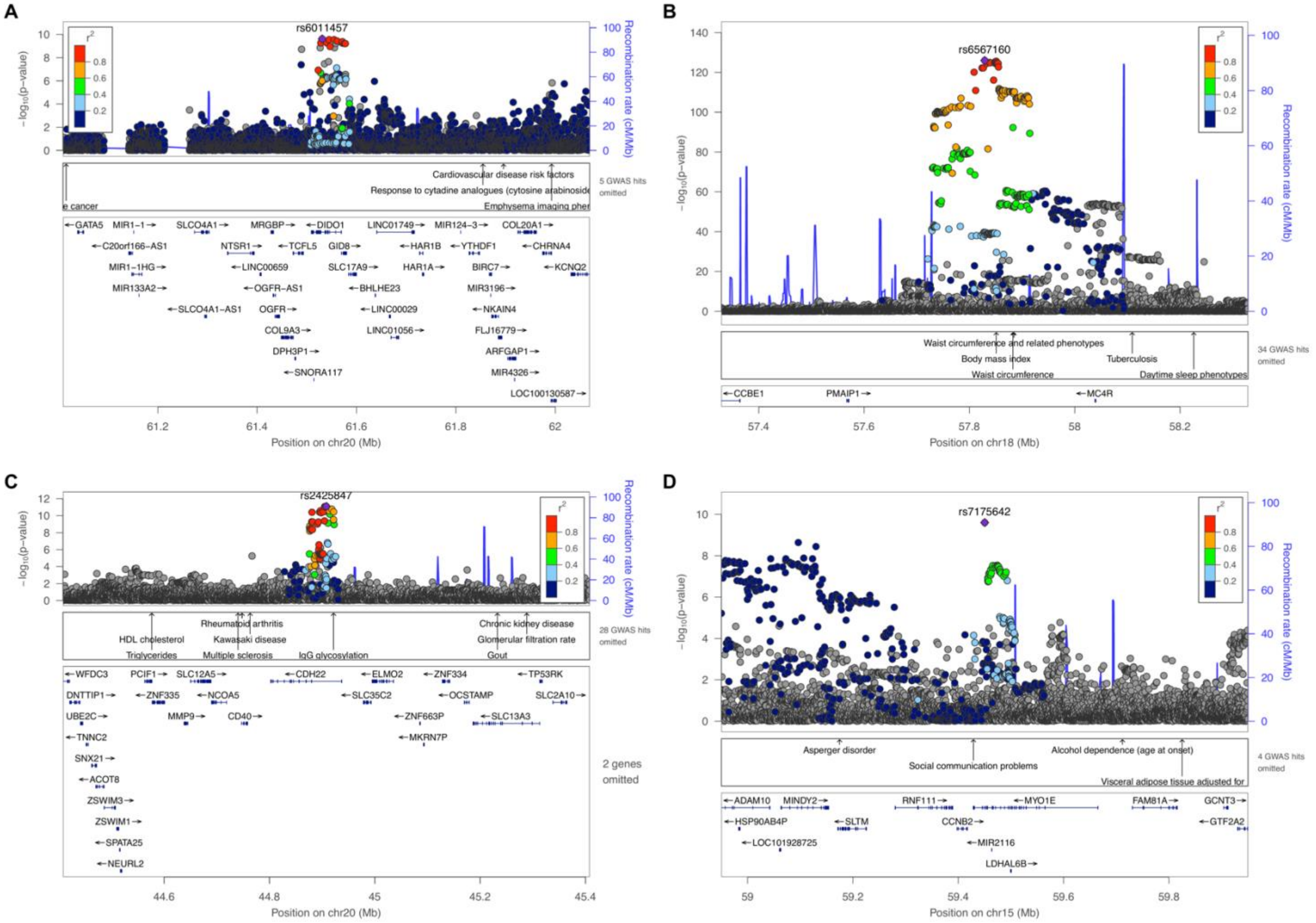

Figure S3 | Common variant associations with adult BMI, related to Figure 1. Locus zoom plots of BMI GWAS signals at the *DIDO1* (A), *MC4R* (B), *SLC12A5* (C) and *SLTM* (D) loci. SNPs are coloured in regard to their level of LD (R^2) towards the lead SNP, with SNPs in grey having an R^2<0.05. Underlying information can be found in Table S8.

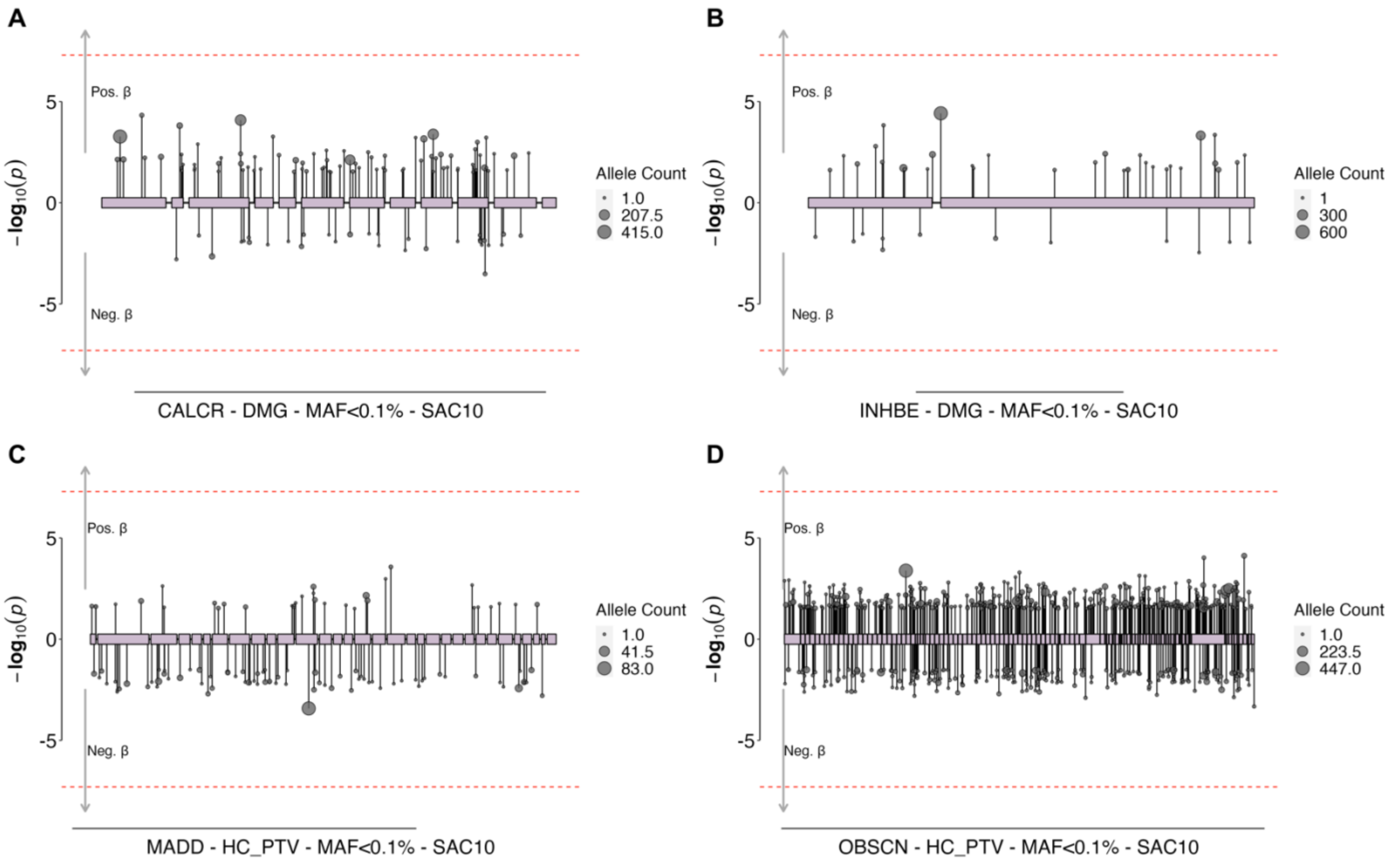

Figure S4 | Exome associations between the identified genes and sex-combined SAC10 in UK Biobank, related to Figure 4. Variant-level associations between variants within *CALCR* (A), *INHBE* (B), *MADD* (C), *OBSCN* (D) and SAC10. Included variants from our discovery analyses had a minor allele frequency (MAF) <0.1% and were annotated to either be high-confidence protein truncating variants or missense variants with a high CADD score (≥25). Each variant is presented as an individual line extending to its association p-value (-log10), in the direction indicating the direction of effect on SAC10 in carriers of the alternate allele, while the point size indicates the number of carriers of each variant (i.e. allele count), as indicated in the figure legend. Extended data can be found in Table S3.

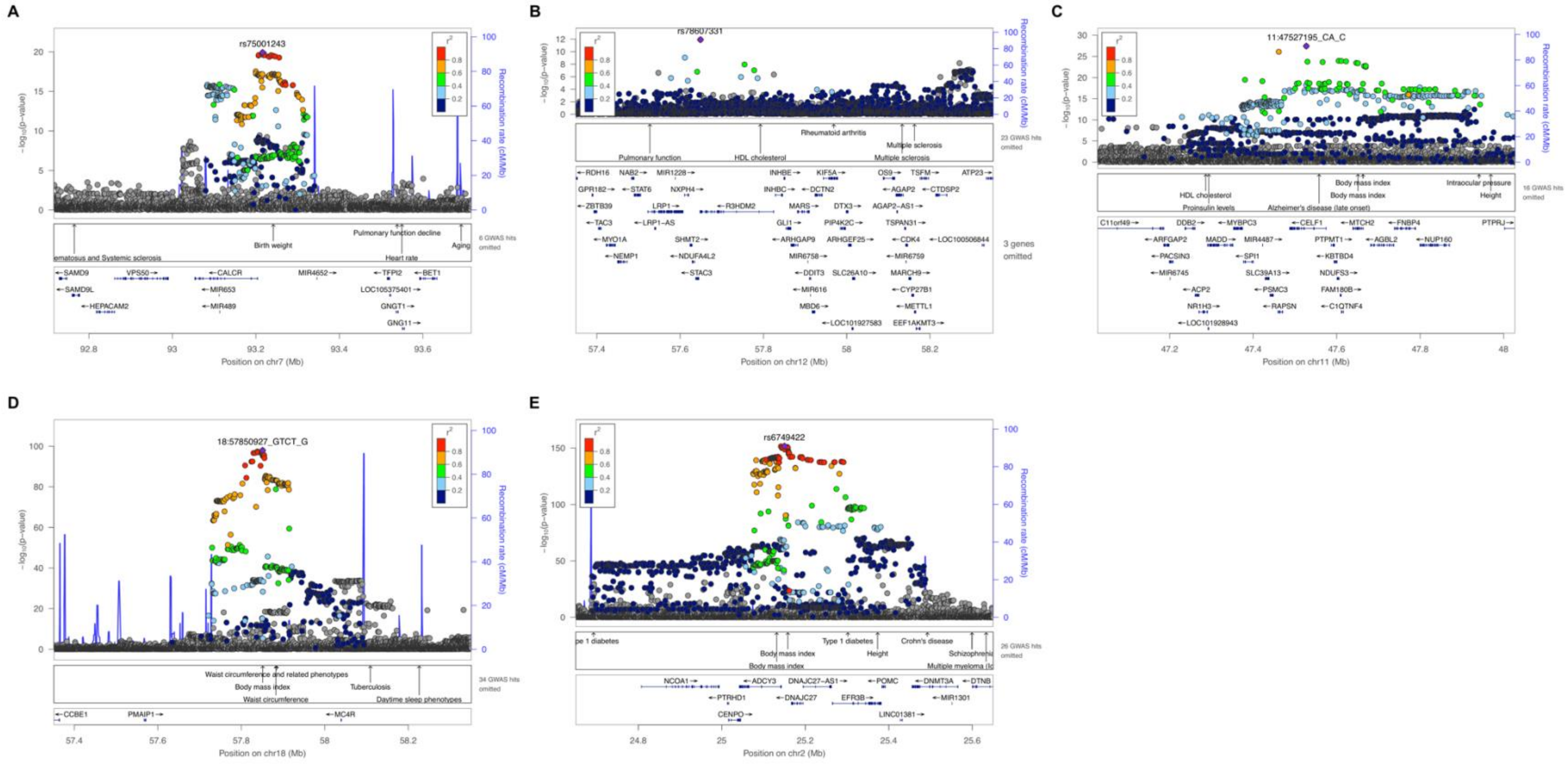

Figure S5 | Common variant associations with comparative body size at age 10, related to Figure 4. Locus zoom plots of the *CALCR* (A), *INHBE* (B), *MADD* (C), *MC4R* (D) and *POMC* (E) loci in the common variant SAC10 GWAS. Underlying data can be found in Table S8.
